# Stathmin regulates mutant p53 stability and transcriptional activity in ovarian cancer

**DOI:** 10.1002/emmm.201201504

**Published:** 2013-04-22

**Authors:** Maura Sonego, Monica Schiappacassi, Sara Lovisa, Alessandra Dall'Acqua, Marina Bagnoli, Francesca Lovat, Massimo Libra, Sara D'Andrea, Vincenzo Canzonieri, Loredana Militello, Marco Napoli, Giorgio Giorda, Barbara Pivetta, Delia Mezzanzanica, Mattia Barbareschi, Barbara Valeri, Silvana Canevari, Alfonso Colombatti, Barbara Belletti, Giannino Del Sal, Gustavo Baldassarre

**Affiliations:** 1 Division of Experimental Oncology 2, Centro di Riferimento Oncologico, National Cancer InstituteAviano, Italy; 2 Department of Experimental Oncology and Molecular Medicine, Fondazione IRCCS Istituto Nazionale dei TumoriMilan, Italy; 3 Department of Biomedical Sciences, University of CataniaCatania, Italy; 4 Division of Pathology, Centro di Riferimento Oncologico, National Cancer InstituteAviano, Italy; 5 LNCIB – Area Science ParkItaly; 6 Dipartimento Scienze della Vita University of TriesteItaly; 7 Surgical Gynecology–Oncology, Centro di Riferimento Oncologico, National Cancer InstituteAviano, Italy; 8 Department of Medical Laboratory, Genetics Section, Pordenone HospitalPordenone, Italy; 9 Department of Pathology, Ospedale Santa ChiaraTrento, Italy; 10 Pathology Fondazione IRCCS Istituto Nazionale dei TumoriMilan, Italy; 11 Department of Scienze e Tecnologie Biomediche, MATI Center of Excellence, University of UdineUdine, Italy; 12 Present address: Department of Molecular Virology, Immunology & Medical Genetics Comprehensive Cancer Center, Ohio State UniversityColumbus, OH, USA; 13 Present address: Department of Biochemistry and Molecular Biology, UT MD Anderson Cancer Center HoustonTX, USA

**Keywords:** carboplatinum, DNA-PK, mutant p53, ovarian cancer, stathmin

## Abstract

Stathmin is a p53-target gene, frequently overexpressed in late stages of human cancer progression. Type II High Grade Epithelial Ovarian Carcinomas (HG-EOC) represents the only clear exception to this observation. Here, we show that stathmin expression is necessary for the survival of HG-EOC cells carrying a p53 mutant (p53^MUT^) gene. At molecular level, stathmin favours the binding and the phosphorylation of p53^MUT^ by DNA-PK_CS_, eventually modulating p53^MUT^ stability and transcriptional activity. Inhibition of stathmin or DNA-PK_CS_ impaired p53^MUT^–dependent transcription of several M phase regulators, resulting in M phase failure and EOC cell death, both *in vitro* and *in vivo*. In primary human EOC a strong correlation exists between stathmin, DNA-PK_CS_, p53^MUT^ overexpression and its transcriptional targets, further strengthening the relevance of the new pathway here described. Overall our data support the hypothesis that the expression of stathmin and p53 could be useful for the identification of high risk patients that will benefit from a therapy specifically acting on mitotic cancer cells.

## INTRODUCTION

The microtubule (MT) destabilizing protein stathmin 1 (also known as oncoprotein 18, OP18, or metablastin; Cassimeris, 2002), is a p53 target gene (Murphy et al, 1999; Singer et al, 2007) and has been associated with increasing metastatic potential and drug resistance (Belletti & Baldassarre, 2011). Accordingly, in human cancer high levels of stathmin 1 (hereafter referred to as stathmin) are almost invariably associated with increased malignancy, metastasis formation and decreased patient survival, suggesting that it could serve as a prognostic marker to identify patients with a more aggressive disease (Belletti & Baldassarre, 2011).

One exception to this scenario is represented by high-grade serous epithelial ovarian carcinomas (EOC) where stathmin overexpression represents a very early event already observable in premalignant lesions (Karst et al, 2011).

Based on clinical behaviour, morphological characteristic and molecular alterations EOCs have been recently divided in two types (type I and type II; Kurman & Shih, 2011), with type II EOC representing a lethal neoplasia with the highest death-to-incidence ratios (Jemal et al, 2011). Type II EOC comprises serous, endometriod and undifferentiated High-Grade EOC (HG-EOC), characterized by the presence of mutations in the tumour suppressor protein p53 in more than 75% of the cases (Kurman & Shih, 2011; Landen et al, 2008). Recent evidence suggests that accumulation of mutant p53 (p53^MUT^) represents the first event in the development of type II HG-EOC (Karst & Drapkin, 2010). These observations make type II HG-EOC an extremely interesting model to clarify how p53^MUT^ is stabilized in human tumours and which are its downstream effectors during the early stages of carcinogenesis. In fact, both in human and mouse models, mutations in p53 do not account *per se* for the accumulation of the protein and it is well established that high expression of p53^MUT^ is mostly found in advanced cancers (Goh et al, 2011). One interesting clinical characteristic of HG-EOCs is that these tumours frequently respond to the Carboplatinum (CBDCA) + Taxol (TAX) chemotherapy, although they eventually develop resistance in a very high percentage of cases (Jemal et al, 2011). Clinical evidences also suggest that overexpression of p53^MUT^ is linked to EOCs chemosensitivity, especially when TAX is used (Cassinelli et al, 2001; Lavarino et al, 2000).

It is now well established that mutations in p53 not only disrupt the functions of the wild type protein but also confer brand new tumour promoting activities, likely influencing the transcription of specific downstream target genes (Girardini et al, 2011; Muller et al, 2011; Oren & Rotter, 2010). Interestingly, in type II HG-EOC increased stathmin expression immediately follows p53^MUT^ accumulation (Karst et al, 2011), suggesting that it could play a specific role acting downstream p53^MUT^.

Here, we investigate the significance of stathmin overexpression in HG-EOC and its relationship with p53^MUT^ expression, demonstrating that stathmin expression is necessary to increase p53^MUT^ protein stability and transcriptional activity. Our results highlight a new regulatory loop that involves stathmin and DNA-PK_CS_ and that is necessary to sustain the gain-of-function activities of p53^MUT^.

## RESULTS

### Stathmin knockdown decreases EOC cell growth and survival, only in the presence of p53^MUT^

**Figure 1:**
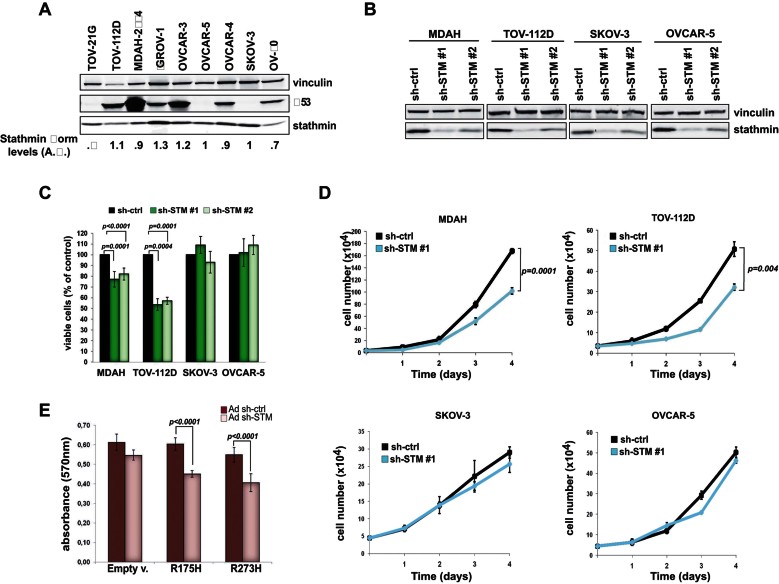
Stathmin silencing affects viability of HG-EOC cells harboring mutations of p53. Source data is available for this figure in the Supporting Information. A. Stathmin and p53 protein expression in total protein extracts from nine EOC cell lines. B. Expression of stathmin in EOC cell lines transduced with control shRNA (sh-ctrl) or two different stathmin shRNAs (sh-STM #1 and sh-STM #2). Vinculin was used as loading control. C. MTS assay comparing cell viability of the indicated EOC cell lines 72 h after shRNAs transduction. Results are expressed as percentage of viable cells respect to sh-ctrl treated cells. D. Growth curves of EOC cells transduced with sh-ctrl or sh-STM #1. E. MTS assay comparing cell viability of SKOV-3 cells expressing p53^R175H^ or p53^R273H^ mutants transduced or not with stathmin shRNA. Results (C–E) represent the mean ± SD of three independent experiments performed in triplicate (growth curve) or quadruplicate (MTS assays). Statistically significant differences are reported. *p* values were calculated using *t*-test.

It has been reported that stathmin and p53 are overexpressed in the very early stages of HG-EOC progression (Karst et al, 2011) and that stathmin is a p53^MUT^ target gene (Singer et al, 2007). We thus decided to evaluate whether these two events were functionally related. To this aim, we first used a panel of nine EOC cell lines for which the p53 status was known (Supporting Information Table S1). Western blot analyses demonstrated that stathmin was expressed at high levels in all tested cell lines (Fig 1A). A loss of function approach, by the interference technology, was then used to evaluate the role of stathmin in EOC cells. Stathmin silencing resulted in decreased cell survival in cell lines harbouring a p53^MUT^ protein (MDAH-2774, TOV112D and OVCAR-8), while had no effect on p53^NULL^ and p53^WT^ expressing cells (Supporting Information Table S1). This result suggested that expression of stathmin and p53^MUT^ could be functionally related in HG-EOC.

Then, we selected five cell lines for further studies; two cell lines presenting hot-spot mutations of p53 (R175H in TOV-112D and R273H in MDAH-2774) both confirmed in homozygosity by direct sequencing (not shown), two cell lines in which p53 was not expressed for homozygous deletion (SKOV-3) or homozygous insertion resulting in a truncated protein (OVCAR-5) and a fifth cell line carrying a wild type p53 protein (TOV-21G; Supporting Information Table S1). Data confirmed that stathmin knockdown impaired cell viability (Fig 1B and C; Supporting Information Fig S1) and inhibited cell growth (Fig 1D; Supporting Information Fig S1C) only in TOV-112D and MDAH-2774 cells (hereafter MDAH), the ones expressing a p53^MUT^ protein. Again, stathmin silencing had no effect on the viability of p53^WT^ expressing cells, TOV-21G (Supporting Information Fig S1F and G). To further validate these observations, we transfected hot-spot mutations of p53, namely p53^R175H^ and p53^R273H^, in p53^NULL^ SKOV-3 cells. Subsequent silencing of stathmin in p53^R175H^ and p53^R273H^ transfected cells, but not in controls, led to decreased cell survival (Fig 1E). Thus, our *in vitro* data established that stathmin is necessary for the survival of p53^MUT^ expressing HG-EOC cells.

### Stathmin is necessary for survival of mitotic cells following DNA damage

**Figure 2:**
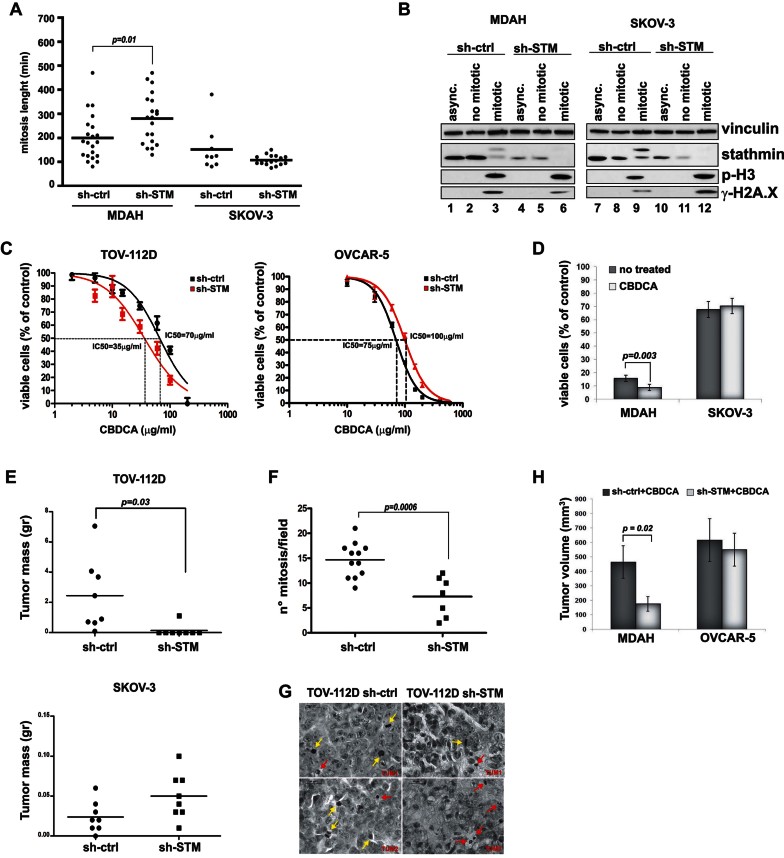
Stathmin expression is necessary for survival of p53^MUT^ HG-EOC cells following DNA damage, *in vitro* and *in vivo* Source data is available for this figure in the Supporting Information. A. M phase length in MDAH and SKOV-3 cells transduced with STM- or ctrl-shRNAs and analysed by time lapse video microscopy. In the scattered dot plot (*n* = 20 mitosis, in each condition) the median value of mitosis lenght (black bar) is reported. B. Western blot analyses of EOC cells harvested by mitotic shake off probed for stathmin, p-Histone H3 (p-H3) and p-Histone H2.X (γH2A.X) expression. Stathmin phosphorylation (higher MW band) and p-H3 confirmed that shaken-off cells were in M phase (lanes 3, 6, 9, 12). C. Evaluation of IC50 in the indicated cell lines treated with increasing doses of CBDCA, in the presence or not of stathmin shRNA. Curves were fitted by nonlinear regression using GraphPad Prism. Results represent the mean (±SD) of three independent experiments performed in quadruplicate. D. Cell survival of stathmin silenced mitotic cells treated or not with CBDCA for 2 h. Results are expressed as percentage of viable cells respect to controls and represent mean ± SD of four independent experiments performed in quadruplicate. E. Subcutaneous growth of TOV-112D (upper panel) and SKOV-3 (lower panel) cells stably transduced with control (sh-ctrl) or stathmin (sh-STM) shRNAs. Results are expressed as scattered dot plot (*n* = 8) with median (black bar) tumour weight. F. Mitotic index of tumours formed by TOV-112D cells described in E. Data are expressed as number of mitoses/field (40× objective) in at least seven randomly selected fields. G. Representative H&E staining of tissue sections described in F and collected using a 100× objective (Bar = 20 µm). Yellow arrows indicate normal or aberrant mitosis, red arrows indicate condensed and fragmented nuclei resembling mitotic catastrophes figures. H. Growth of MDAH and OVCAR-5 xenografts in nude mice treated with intratumoural injection of control (sh-ctrl) or stathmin (sh-STM) sh-RNAs and with intraperitoneal treatment of CBDCA. Results represent the mean (±SD) volume of explanted tumours (*n* = 5). *p* values were calculated using *t*-test using or Mann–Whitney test. Statistically significant differences are reported in each panel.

Next, we asked how stathmin silencing could induce cell death in p53^MUT^ HG-EOC cells. We first used video time-lapse microscopy on MDAH (p53^MUT^) and SKOV-3 (p53^NULL^) cells transduced with control- or stathmin specific-shRNA and recorded cells for 48 h. Using this approach we could appreciate that stathmin-silenced MDAH, but not SKOV-3, cells stalled in mitosis and eventually died (not shown). Accurate evaluation of the progression through the M phase in single cells revealed that stathmin silencing significantly affected the length of M phase in MDAH cells, while having no effect in SKOV-3 cells (Fig 2A). Moreover, we observed that HG-EOC mitotic cells expressing p53^MUT^ (MDAH and TOV-112D), displayed an unusually high presence of damaged DNA, as evaluated by immunofluorescence analyses of phosphorylated histone H2A.X on S139 (γH2A.X), used as marker of DNA damage, and of phospho-S10-histone-H3, used as marker of mitosis (Supporting Information Fig S2A and B). This finding was confirmed by Western blot analysis of γH2A.X in mitotic MDAH and SKOV-3 cells, displaying a significantly higher level of damaged DNA in MDAH respect to SKOV-3 cells (compare lanes 3 and 9 in Fig 2B). This increase of DNA damage was specifically due to the presence of mutant p53, since could be recapitulated in mitotic SKOV-3 cells transfected with p53^R175H^ or p53^R273H^ (Supporting Information Fig S2C and D). Altogether these data indicated that during mitosis HG-EOC cells expressing p53^MUT^ accumulate a significantly higher amount of damaged DNA and that, in this setting, stathmin expression is necessary for the proper completion of M phase.

### Stathmin expression influences p53^MUT^ HG-EOC cell response to chemotherapy

Given the above data, we hypothesized that stathmin could be also implicated in the survival of p53^MUT^ HG-EOC cells exposed to the CBDCA-TAX therapy, since these drugs act either by inducing DNA adducts followed by S phase and G2/M cell cycle block (known to be impaired in p53^MUT^ expressing cells) or by directly targeting the mitotic spindle. In order to test this hypothesis, we silenced stathmin expression and treated EOC cells with CBDCA or TAX, to induce cell death. Treatment with increasing doses of the above drugs demonstrated that stathmin silencing sensitized EOC cells to both TAX (Supporting Information Fig S3A), as expected given stathmin's role in the formation and disruption of the mitotic spindle (Rubin & Atweh, 2004), and CBDCA (Fig 2C and Supporting Information Fig S3B). Interestingly, in both cases stathmin protected from drug-induced cell death only in p53^MUT^ expressing HG-EOC cells (Fig 2C and Supporting Information Fig S3A and B).

These results suggested that HG-EOC cells expressing p53^MUT^ would be particularly sensitive to stathmin silencing during mitosis, either in presence or absence of CBDCA. To test this possibility MDAH and SKOV-3 cells were exposed to low doses of CBDCA for 2 h in order to increase DNA damage, isolated by mitotic shake-off and then assayed for their viability. In the absence of stathmin, MDAH (p53^MUT^) cells did not properly recover from M phase, an effect further enhanced by the CBDCA-induced DNA damage (Fig 2D). On the contrary, stathmin silencing in mitotic SKOV-3 (p53^NULL^) cells slightly decreased cell survival and this effect was not influenced by CBDCA treatment (Fig 2D). These data strongly indicated that stathmin is necessary for the survival of mitotic DNA-damaged HG-EOC cells and suggested that stathmin could be implicated in the resistance of HG-EOC cells to CBDCA when p53^MUT^ is expressed.

### Stathmin is necessary for the *in vivo* survival of HG-EOC cells expressing p53^MUT^

Next we injected subcutaneously TOV-112D cells (p53^MUT^) or SKOV-3 cells (p53^NULL^) in nude mice, silenced or not for stathmin expression. Stathmin silencing almost completely abrogated the ability of TOV-112D to grow in mice, (Fig 2E), while having no effect on the *in vivo* growth of SKOV-3 cells (Fig 2E). Similar results were observed when TOV-112D cells were injected intraperitoneally (Supporting Information Fig S3C). Pathological analyses of explanted tumours demonstrated that in stathmin silenced TOV-112D tumours the number of mitosis was significantly reduced respect to controls (Fig 2F). Along with normal mitotic figures, control tumours displayed some mitotic aberrations (Fig 2G, yellow arrows) and some figures with condensed chromatin resembling cells died for mitotic catastrophe (Fig 2G, red arrows). Conversely, tumours from stathmin silenced cells showed only few mitoses (Fig 2G, yellow arrows), with very rare mitotic aberrations and a net preponderance of mitotic catastrophes (Fig 2G, red arrows). These data support the possibility that, also *in vivo*, stathmin is necessary for the survival of p53^MUT^ mitotic cells.

Moreover, intratumoural delivery of stathmin shRNAs in MDAH (p53^MUT^) and OVCAR-5 (p53^NULL^) cells confirmed that, also in an *in vivo* setting, stathmin silencing resulted in decreased cell survival in a p53^MUT^-dependent manner (Fig 2H). Consistently with *in vitro* results, the inhibition of tumour growth was especially evident when mice were treated with CBDCA (Fig 2H). Overall, these findings pointed to stathmin as a critical mediator of growth and drug resistance of p53^MUT^ HG-EOC, both *in vitro* and *in vivo*.

### Stathmin controls p53^MUT^ protein stability

**Figure 3:**
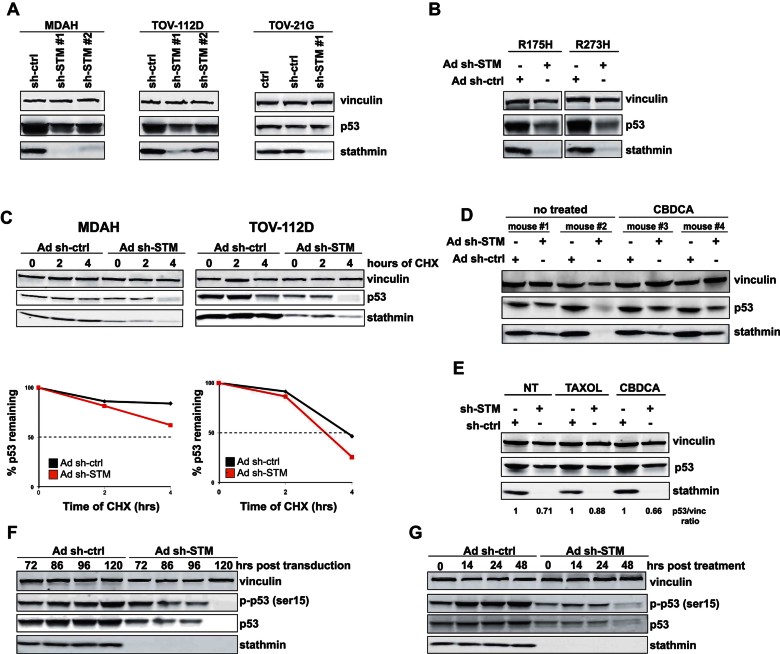
Stathmin knockdown controls p53^MUT^ stability. Source data is available for this figure in the Supporting Information. A. Western blot analysis of p53 and stathmin expression in MDAH, TOV-112D and TOV-21G cells, transduced or not with stathmin shRNAs. B. Western blot analysis of stathmin and p53 expression in SKOV-3 cells expressing p53^R175H^ or p53^R273H^ mutants, transduced or not with stathmin shRNA. C. Western blot analysis of p53 and stathmin expression in MDAH and TOV-112D cells transduced with Ad sh-ctrl or Ad sh-STM and treated with 10 µg/ml CHX for 2 or 4 h, as indicated. Densitometric analysis of p53 expression (normalized respect to vinculin expression) is reported in the bottom graphs. D. Western blot analysis of p53 and stathmin expression in MDAH xenograft tumours after transduction with control (Ad sh-ctrl) or stathmin shRNAs (Ad sh-STM) and treated or not with CBDCA. E. Western blot analysis of p53 and stathmin expression in MDAH cells transduced with sh-ctrl or sh-STM and treated with TAXOL or CBDCA, as indicated. Densitometric analysis of normalized p53 expression is reported. F,G. Time course analysis of p53 and pS15-p53 protein expression in MDAH cells after stathmin knockdown (F) and after CBDCA treatment (G). Vinculin was used as loading control in all Western blots.

To understand the molecular mechanism connecting stathmin and p53^MUT^, we first tested whether p53 expression changed in response to variation in the levels of stathmin in EOC cells expressing p53^MUT^ or p53^WT^. Results showed that stathmin knock-down resulted in concomitant down-regulation of mutant p53 protein, while it had no effect on the levels of p53^WT^ (Fig 3A). Importantly, stathmin knock-down was associated to reduced p53^R175H^ and p53^R273H^ ectopically expressed in SKOV-3 cells (Fig 3B), suggesting that stathmin controlled p53^MUT^ mRNA or protein stability. Accordingly, the use of the protein synthesis inhibitor cycloheximide (CHX) demonstrated that stathmin knockdown reduced p53^MUT^ half-life in both TOV-112D and MDAH cells (Fig 3C).

Taking advantage of the previously described *in vivo* experiments (Fig 2), we analysed tumours from MDAH cells intratumourally injected with stathmin shRNA and verified that stathmin knockdown was associated with a marked downregulation of p53, also when animals were co-treated with CBDCA (Fig 3D). Accordingly, *in vitro* exposure of MDAH cells to CBDCA resulted in increased expression of p53^MUT^ protein that was completely prevented by stathmin silencing (Fig 3E). The net reduction of p53^MUT^ expression by stathmin was 30% in control and 40% in CBDCA treated cells (Fig 3E), suggesting that stathmin is important for p53^MUT^ protein stabilization, particularly after DNA damage.

### Stathmin modulates p53^MUT^ phosphorylation

It is well accepted that both wild type and mutant p53 are stabilized following DNA damage (Terzian et al, 2008). Phosphorylation of p53 on serine 15 plays a major role in the control of damage-induced p53 stabilization (Kruse & Gu, 2009; Sengupta & Harris, 2005). Using a time course analysis, we looked at the expression of p53 and its phosphorylation on serine 15 (pS15) in cells transduced with control- or stathmin specific-shRNA. Silencing of stathmin led to a pronounced decrease in both S15 phosphorylation and total level of p53 protein (Fig 3F). This experiment was then performed treating cells with CBDCA followed by release in complete medium for the indicated time. After 14 h, control cells displayed a marked induction of pS15 that remained stable up to 48 h and was accompanied by a twofold increase in the levels of total p53. Increase of both S15 phosphorylation and total level of p53 protein was almost completely prevented when stathmin was silenced (Fig 3G). Overall, these data demonstrated that stathmin is necessary for the basal and the CBDCA induced p53^MUT^ stabilization, likely through the modulation of S15 phosphorylation.

### Stathmin modulates p53^MUT^ activity via the regulation of DNA-PK expression

To get more insights on the pathway implicated in the observed stathmin-induced stabilization of p53^MUT^, we performed a proteomic screening in CBDCA-treated MDAH cells, silenced or not for stathmin. Statistical analyses of the results indicated that stathmin knockdown heavily affected the DNA-PK pathway (Supporting Information Table S2). Not only the expression of DNA-PK catalytic subunit (hereafter DNA-PK) was reduced, but also the levels of several DNA-PK co-regulators, such as PP6C (Mi et al, 2009), PP1 (Wong et al, 2009), HSP90 (Dote et al, 2006) and PKAR2α (Huston et al, 2008), were altered in stathmin silenced cells compared to controls. These data were in complete agreement with the observation that both pS15 of p53 (Fig 3F and G) and γH2A.X (compare lanes 3 and 6 in Fig 2B), two known DNA-PK substrates, were significantly reduced in stathmin silenced cells.

**Figure 4:**
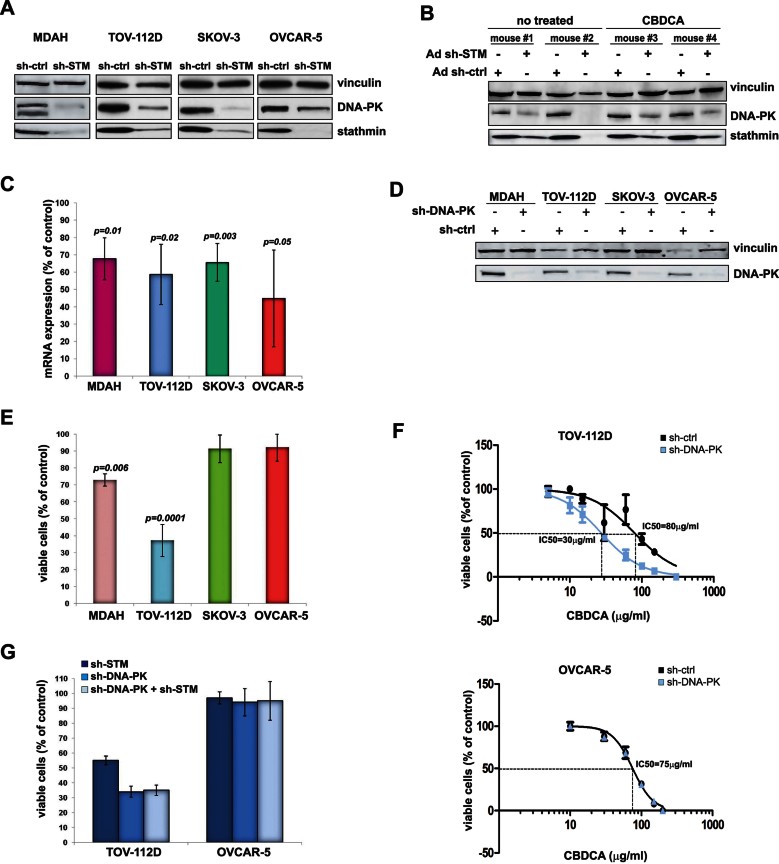
Stathmin regulates survival of p53^MUT^ HG-EOC cells by controlling DNA-PK expression. Source data is available for this figure in the Supporting Information. A,B. DNA-PK expression in EOC cells (A) and in MDAH xenograft tumours (B) after transduction with control (sh-ctrl) or stathmin shRNAs (sh-STM) in the presence or not of CBDCA treatment, as indicated. C. qRT-PCR analyses of DNA-PK expression in stathmin silenced cells. Data are expressed as percent of mRNA levels with respect to control (sh-ctrl) transduced cells and represent the mean (±SD) of three independent experiments performed in duplicate. D. Western blot analysis of DNA-PK expression in cells stably transduced with sh-ctrl or sh-DNA-PK, as indicated. E. MTS assay comparing cell viability of the EOC cell lines described in D. Data are expressed as percent of viable cells with respect to control transduced cells and represent the mean of three independent experiments performed in quadruplicate. F. Evaluation of IC50 in the indicated EOC cell lines for increasing doses of CBDCA. Data represent the mean of three independent experiments performed in quadruplicate. G. MTS assay comparing cell viability of TOV-112D and OVCAR-5 cell lines stable transduced with sh-DNA-PKcs or sh-ctrl in combination or not with stathmin silencing. In E and G results are expressed as percentage of viable cells with respect to sh-ctrl transduced cells and represent mean (±SD) of three independent experiments performed in quadruplicate. Significant *p* values are reported in the figure and were calculated using *t*-test.

Western blot analyses confirmed that stathmin knockdown downregulated DNA-PK expression in control and CBDCA treated cells, both *in vitro* (Fig 4A and Supporting Information Fig S4A) and *in vivo* (Fig 4B). However, this effect was not dependent on the presence of p53^MUT^ expression since it was observed also in cells p53^NULL^ (Fig 4A). Quantitative RT-PCR analysis demonstrated that stathmin knockdown significantly decreased DNA-PK mRNA expression (Fig 4C), suggesting that it controlled DNA-PK transcriptional regulation and/or mRNA stability. Conversely, the expression of ATM and ATR, two other kinases involved in the regulation of p53 S15 phosphorylation following DNA damage (Kruse & Gu, 2009), were not altered by stathmin silencing (Supporting Information Fig S4B), confirming a specific role of DNA-PK in this process.

As previously observed for stathmin (Fig 1), impairment of DNA-PK activity by means of specific sh-RNAs or of the specific inhibitor NU7441 (Leahy et al, 2004) decreased EOC cell survival (Fig 4D and E, and Supporting Information Fig S4C) and increased the sensitivity to CBDCA (Fig 4F), only when p53^MUT^ was present. Simultaneous knock-down of stathmin and DNA-PK did not result in enhanced cell death respect to the silencing of DNA-PK alone (Fig 4G), thus supporting the hypothesis that stathmin and DNA-PK activities lie along the same molecular axis.

**Figure 5:**
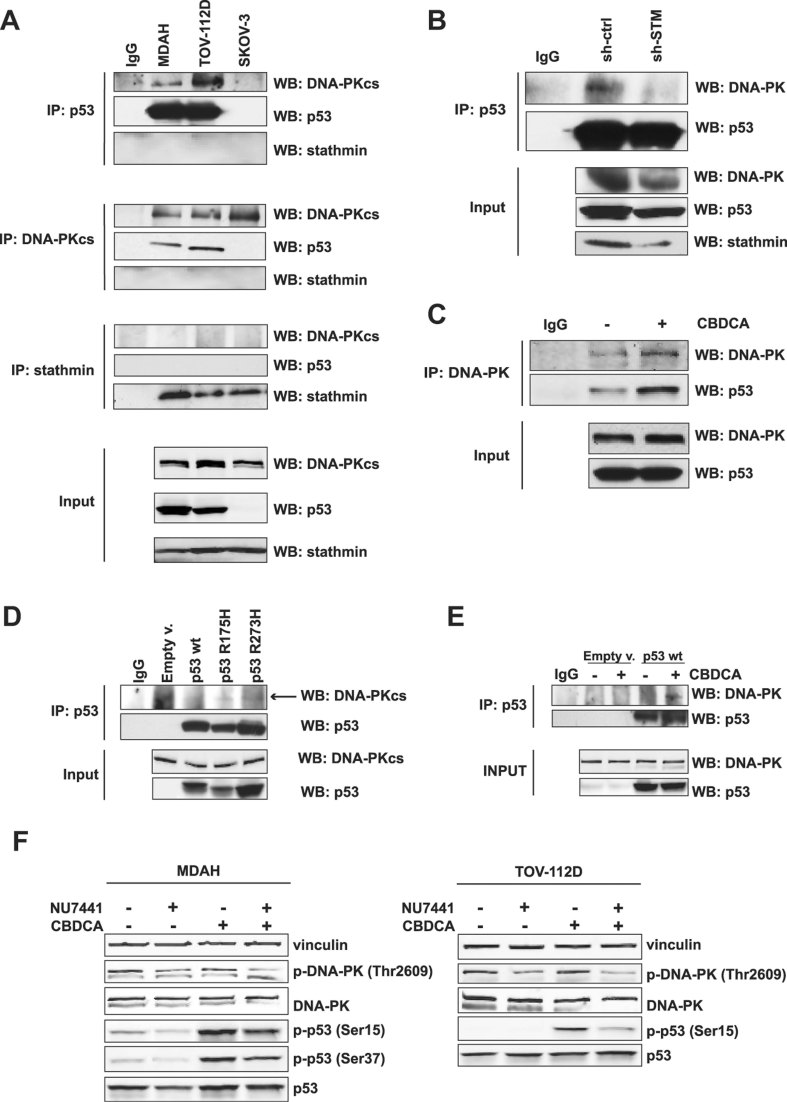
DNA-PK binding to p53^MUT^ is regulated by DNA damage and stathmin expression. Source data is available for this figure in the Supporting Information. A. Co-immunoprecipitation analyses in protein lysates derived from the indicated cell lines. Total cell lysates (input, lower panels) were immunoprecipitated with the indicated antibodies (or control IgG) and probed for DNA-PK, stathmin and p53 expression. B. Western blot analysis of DNA-PK and p53 expression in p53-immunoprecipitated lysates (input, lower panels) from MDAH-sh-STM, or sh-ctrl. IgG indicates a negative control in which lysate was immunoprecipitated using an unrelated antibody. C. Western blot analysis of DNA-PK and p53 expression in lysates (input, lower panels) derived from MDAH cells treated or not with CBDCA and immunoprecipitated with specific anti-DNA-PK antibody or control IgG, as indicated. D. Co-immunoprecipitation analyses of protein lysates derived from SKOV-3 cells transfected with the indicated p53 cDNAs, immunoprecipitated with anti-p53 specific antibodies (or controls IgG), as indicated, and probed for DNA-PK, and p53 expression. The expression levels of p53 and DNA-PK in total cell lysates are reported in the lower panels (input). E. Co-immunoprecipitation analyses of protein lysates derived from SKOV-3 cells transfected with p53^WT^ cDNA and treated with CBDCA for 3 h. Lysates (input, lower panels) were immunoprecipitated and probed with the indicated antibodies (or control IgG). F. Western blot analysis of phosphorylated DNA-PK (pThr 2609) and p53 (S15 and S37) in MDAH and TOV-112D cells, treated for 14 h with NU7441 alone or in combination with CBDCA and analysed 6 h after treatment. Vinculin was used as loading control.

The ability of DNA-PK to bind and phosphorylate wild type p53 protein on serine 15 and 37 has been described (Lees-Miller et al, 1992; Shieh et al, 1997, 1999; Woo et al, 2002) and represent modifications able to regulate p53 function and stability, especially in response to DNA damage (Kruse & Gu, 2009; Sengupta & Harris, 2005; Shieh et al, 1999). First, we assessed whether DNA-PK was able to bind and phosphorylate p53^MUT^ protein in HG-EOC cells. We used MDAH (p53^R273H^), TOV-112D (p53^R175H^) and SKOV-3 (p53^NULL^, used as negative control) cells to verify the association between DNA-PK and p53^MUT^ endogenous proteins. The two endogenous proteins readily and specifically co-immunoprecipitated in both cell lines expressing the mutant forms of p53 (Fig 5A). Yet, in the same condition we were not able to see any association between stathmin and DNA-PK or stathmin and p53 (Fig 5A), indicating that stathmin was not a direct partner of this complex. Importantly, stathmin silencing in MDAH cells significantly impaired the DNA-PK/p53^MUT^ interaction, likely acting on the expression DNA-PK (Fig 5B).

Since we observed an enhancement of stathmin-dependent phosphorylation of S15 p53^MUT^ after CBDCA treatment (Fig 3) we asked whether the platinum-induced damage had an effect on DNA-PK-p53^MUT^ interaction. Data reported in Fig 5C demonstrate that 3 h of CBDCA treatment of MDAH cells was sufficient to increase by twofold the amount of p53 bound to DNA-PK, reinforcing the hypothesis that DNA-PK is at least partially responsible for the basal and platinum-induced p53^MUT^ S15 phosphorylation in HG-EOC cells. As a further control, we transiently overexpressed p53^WT^, p53^R175H^ or p53^R273H^ in p53^NULL^ SKOV-3 cells and observe no binding between endogenous DNA-PK and wild type p53 protein while, in the same conditions, both mutants were able to bind DNA-PK (Fig 5D). However, an interaction between DNA-PK and overexpressed p53^WT^ could be observed following CBDCA treatment (Fig 5E), confirming previous observations (Shieh et al, 1997; Woo et al, 2002).

We then asked whether the interaction between p53^MUT^ and DNA-PK effectively led to p53 phosphorylation. In EOC cells p53-S15 phosphorylation was at least partially dependent on DNA-PK activity, as judged by the effects of the specific DNA-PK inhibitor, NU7441 (Fig 5F). In both p53^MUT^ cell lines basal levels and CBDCA-induced phosphorylation of p53^MUT^ on S15 and S37 were strongly inhibited by impairment of DNA-PK activity with NU7441 (Fig 5F).

**Figure 6:**
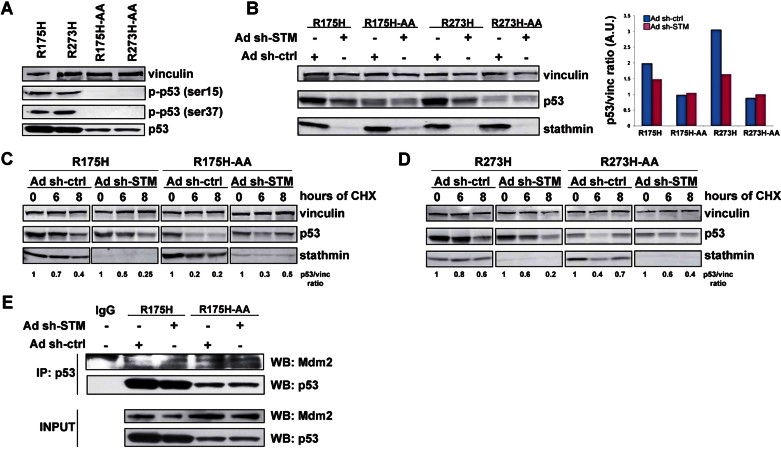
Stathmin regulates p53^MUT^ stability modulating Ser15 and 37 phosphorylations. Source data is available for this figure in the Supporting Information. A. Western blot analysis of total p53, pS15-p53 and pS37-p53 in SKOV-3 transfected with the indicated mutants. B. Western blot analysis of p53 and stathmin expression in SKOV-3 cells transfected with the indicated p53 mutants and then transduced with ctrl- or STM-shRNAs. Vinculin was used as loading control. On the right, the densitometric analysis of the blots is reported, expressed as normalized p53 expression (p53/vinculin ratio). C,D. Western blot analyses of p53 and stathmin expression in SKOV-3 cells transfected with p53^R175H^ and p53^R175H-AA^ (C) or p53^R273H^ and p53^R273H-AA^ (D), transduced with ctrl- or STM-shRNAs and then treated with cycloheximide for 6 and 8 h. Vinculin was used as loading control. E. Co-immunoprecipitation analyses of protein lysates derived from SKOV-3 cells stable transfected with p53^R175H^ and p53^R175H-AA^, transduced with ctrl- or STM-shRNAs, immunoprecipitated with anti-p53 specific antibody (or controls IgG), as indicated, and probed for Mdm2 and p53 expression. The expression levels of p53 and Mdm2 in total cell lysates are reported in the lower panels (input).

Data collected so far suggested that stathmin impinged on p53^MUT^ stability by interfering with its phosphorylation on S15 (and S37), through the down modulation of DNA-PK expression. We thus reasoned that a p53^MUT^ protein unphosphorylable on S15 and S37 would become insensitive to stathmin silencing. To test this possibility we generated p53^R175H^ and p53^R273H^ proteins in which S15 and S37 were substituted with alanine (p53^R175H-AA^ and p53^R273H-AA^; Fig 6A) and expressed them in SKOV-3 cells to analyse their expression and stability, in the presence or not of stathmin silencing. In basal conditions, the expression of both p53^R175H-AA^ and p53^R273H-AA^ was twofolds and threefold lower than the corresponding mutant (Fig 6B), respectively, although their mRNA level was equally expressed, as evaluated by qRT-PCR (data not shown), suggesting again that phosphorylation of S15 and S37 of p53^MUT^ proteins is important for p53 stability, also in basal conditions. As hypothesized, silencing of stathmin strongly reduced p53^R175H^ and p53^R273H^ expression while it had no effect on the p53^R175H-AA^ and p53^R273H-AA^ mutants levels (Fig 6B). Treatment with CHX demonstrated that the half-life of the unphosphorylable mutants was significantly reduced respect to the p53^MUT^ proteins and silencing of stathmin significantly affected only p53^R175H^ and p53^R273H^ stability, but not that of p53^R175H-AA^ and p53^R273H-AA^ mutants (Fig 6C and D). These results demonstrate that stathmin concurs to p53^MUT^ stability by acting on its S15 and S37 phosphorylation. It has been reported that p53^MUT^ could be ubiquitinated and degraded at least in part via the binding to the E3 ligase MDM2 (Walerych et al, 2012). We thus tested whether phosphorylation of S15 and 37 could be implicated in the binding between MDM2 and p53^MUT^ in HG-EOC cells. Strikingly, in SKOV-3 cells expressing p53^R175H^ stathmin silencing increased of about twofolds the amount of MDM2 bound to p53. Moreover, when the p53^R175H-AA^ mutant was used the interaction between MDM2 and p53^MUT^ was readily observed and was independent on the expression of stathmin (Fig 6E). Altogether our data indicated that regulation of p53^MUT^ stability by stathmin is dependent on the phosphorylation of the protein on S15 and 37, which in turn prevented the interaction of the mutant protein with the E3 ligase MDM2.

### Stathmin modulates p53^MUT^ transcriptional activity

**Figure 7:**
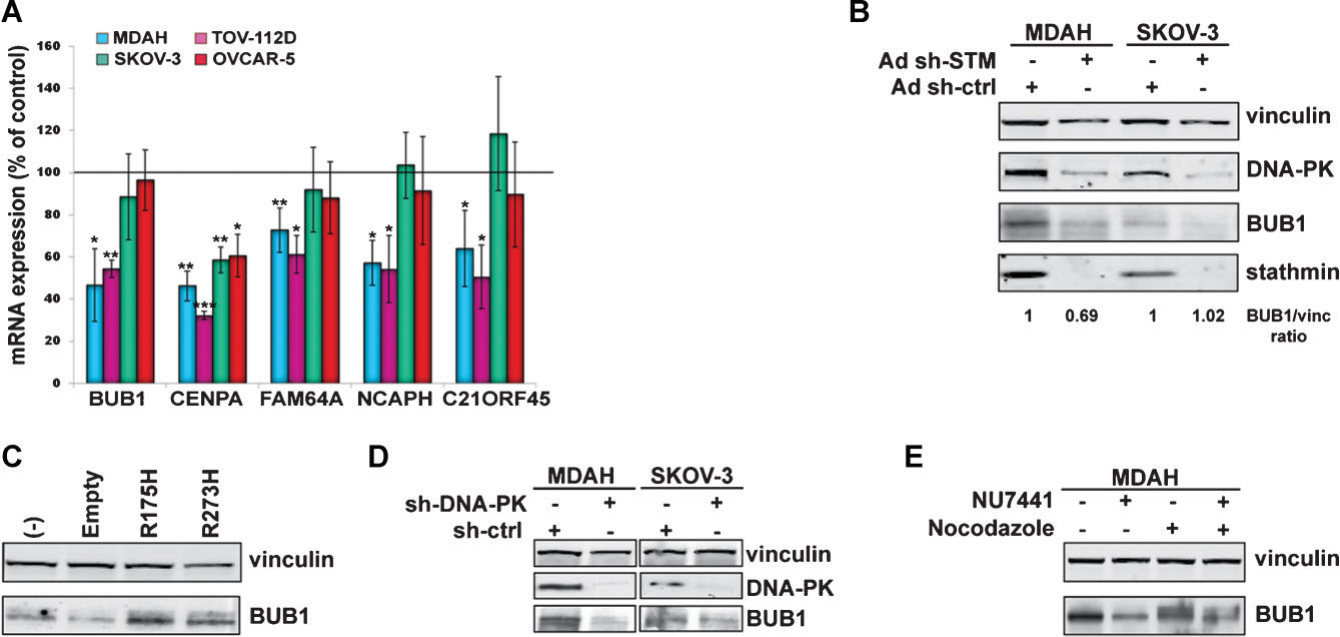
Stathmin modulates p53^MUT^ transcriptional activity. Source data is available for this figure in the Supporting Information. A. qRT-PCR analyses of the indicated genes in EOC cell lines, transduced with Ad sh-STM or Ad sh-ctrl. Data are expressed as percentage of mRNA expression in sh-STM cells respect to sh-ctrl cells and represent the mean (±SD) of at least of three independent experiments. * *p* ≤ 0.05, ** *p* ≤ 0.001 and *** *p* ≤ 0.0001. B. Western blot analysis of DNA-PK, BUB1 and stathmin expression in MDAH and SKOV-3 cells, transduced with Ad sh-STM or Ad sh-ctrl. Numbers at the bottom of the panel report the densitometric analysis of normalized BUB1 expression and represent the mean of two experiments. C. BUB1 protein expression in SKOV-3 cells expressing p53^R175H^ or p53^R273H^ mutants. D. BUB1 protein expression in MDAH and SKOV-3 transduced with DNA-PK or ctrl shRNA. E. BUB1 protein expression in MDAH cells treated for 18 h with 10 µM of NU7441 alone or in combination with 200 nM of Nocodazole. Vinculin was used as loading control in all western blots.

Phosphorylation of p53 on S15 by DNA-PK is necessary for its binding to the co-activator p300/CBP (Lambert et al, 1998). Moreover, it has been reported that phosphorylated p53^MUT^ binds more efficiently to p300 and this, in turn, increases its transcriptional activity (Valenti et al, 2011). We then asked whether the phosphorylation of p53 on S15/37 by DNA-PK could be implicated in the modulation of p53^MUT^ transcriptional activity also in our context. To prove this hypothesis we took advantage of a recent report showing that p53^MUT^ is able to specifically induce the transcription of a panel of genes involved in the control of cell growth and invasion in breast cancer (Girardini et al, 2011). A ten-genes p53^MUT^ signature, comprising 5 genes involved in the M phase progression, is proposed (Girardini et al, 2011). These genes include FAM64A, a protein involved in the regulation of the metaphase to anaphase transition (Zhao et al, 2008), CENP-A, a centromere protein involved in the control of chromosome segregation (Verdaasdonk & Bloom, 2011), NCAPH, a regulatory subunit of the condensin complex (Cabello et al, 2001), C21ORF45, a protein essential for centromere/kinetochore structure and function (Fujita et al, 2007) and BUB1 kinase, a master regulator of the spindle checkpoint (Elowe, 2011). Based on the biological data indicating a major role of stathmin in the survival of M phase HG-EOC cells expressing p53^MUT^ (Fig 2), we decided to examine if p53^MUT^ was able to modulate the transcription of these selected genes, in a stathmin dependent manner. Strikingly, all analysed transcripts, with the exception of CENP-A, were specifically and significantly downregulated when stathmin was silenced and only in p53^MUT^ HG-EOC cells (Fig 7A). We validated the qRT-PCR data by Western blot using BUB1 as readout, and confirmed that it is expressed at higher levels in p53^MUT^ EOC cells both at protein and mRNA levels (Supporting Information Fig S5). Moreover, in accord with qRT-PCR data, stathmin silencing was able to reduce BUB1 protein expression only in p53^MUT^ cells (Fig 7B). Noteworthy, the expression of BUB1 was specifically induced in SKOV-3 overexpressing either the p53^R175H^ or p53^R273H^ mutant (Fig 7C). Next, we evaluated the relevance of p53 S15/37 phosphorylation by DNA-PK in modulating its transcription efficiency, using either DNA-PK specific shRNAs or the NU7441 inhibitor. In both cases, impairment of DNA-PK expression (Fig 7D) or activity (Fig 7E) strongly reduced BUB1 levels in a p53^MUT^-dependent manner. Finally, we increased the M phase population by treating cells with Nocodazole and demonstrated that DNA-PK activity is necessary for proper expression of BUB1 in p53^MUT^ mitotic cells (Fig 7E).

### Stathmin overexpression is linked to p53 mutation in type II EOC

**Figure 8:**
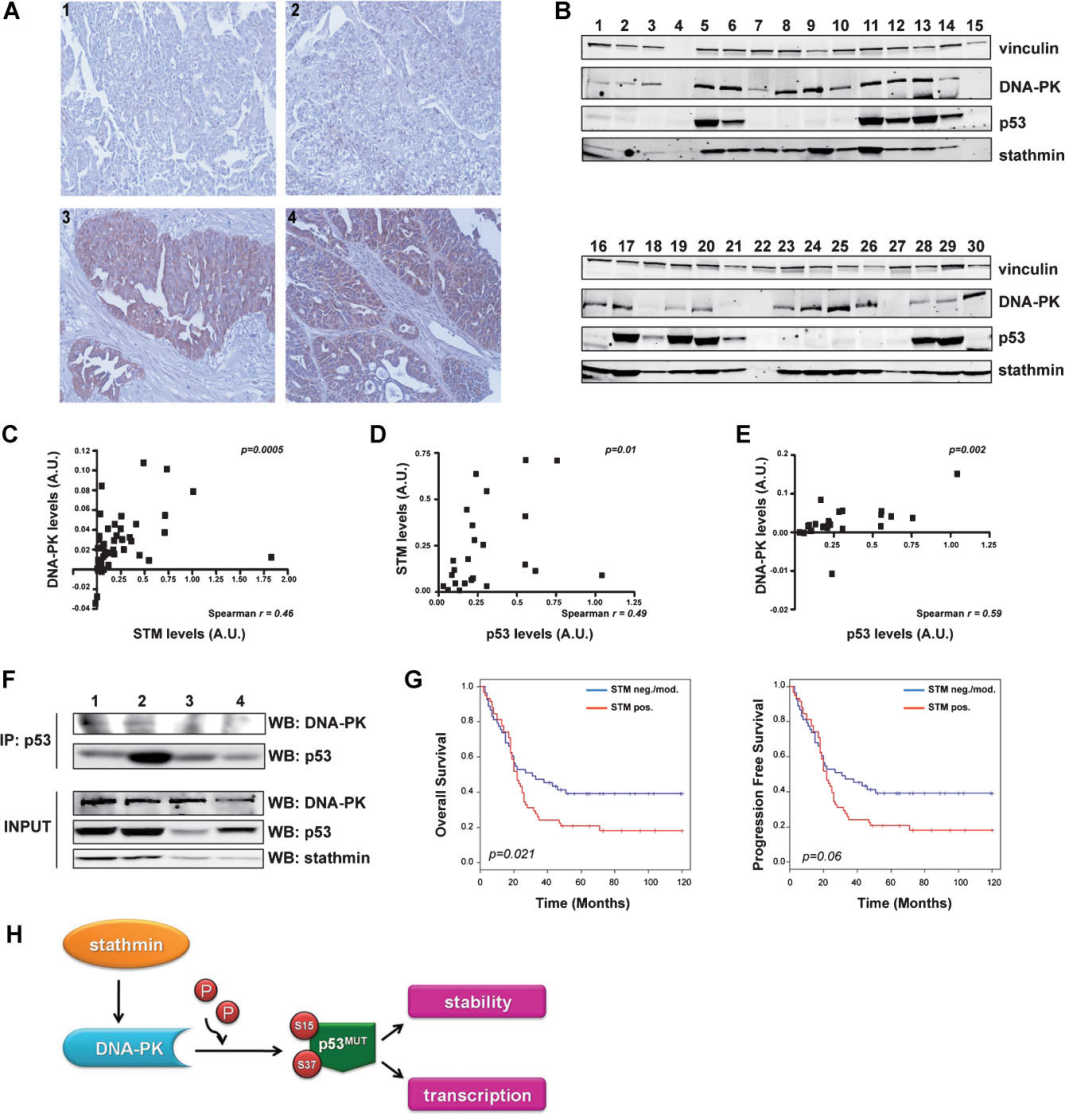
Stathmin overexpression identifies low-surviving ovarian cancer patients. Source data is available for this figure in the Supporting Information. A. Representative immunohistochemical staining of formalin-fixed, paraffin-embedded EOC tissue sections negative/moderate (panel 1: serous G3; panel 2: endometrioid G2) and high (panel 3: serous G2; panel 4: serous G3) for stathmin expression. Original magnification 200×. B. Representative Western blot analyses of p53, stathmin and DNA-PK expression in total protein extracts from primary high-grade serous carcinoma samples. C–E. Spearman's correlation analysis between stathmin and DNA-PK (C), p53 and stathmin (D) or p53 and DNA-PK (E) expression in primary serous carcinomas. F. Co-immunoprecipitation of p53 and DNA-PK in primary high-grade serous carcinoma samples. Total protein lysates from tumours expressing high (lanes 1, 2) or low (lanes 3,4) levels of stathmin expression (input, lower panels) were immunoprecipitated using anti-p53 antibody and then immunoblotted using anti-DNA-PK and anti-p53 antibodies, as indicated. G. Kaplan Meyer estimate of overall (left) and progression free (right) survival, following stratification for stathmin expression. *p* values were calculated using the log-rank test. H. Schematic representation depicting the role of stathmin in EOC carrying a p53^MUT^ protein. High stathmin levels in EOC are necessary for proper expression of DNA-PK that, in turn, positively regulates p53^MUT^ stability and transcriptional activity through S15/S37 phosphorylation.

We next asked whether this newly discovered regulatory loop characterized *in vitro* had significance in human pathology. To test our findings we analysed a series of 72 HG-EOC samples collected at the National Cancer Institute of Milan, between 1999 and 2001 that comprised only type II HG-EOCs for which the p53 mutational status was known (case material B, in Supporting Information Table S3). The immunohistochemical analysis demonstrated that stathmin displayed a specific cytoplasmic pattern of expression (Fig 8A) and that its overexpression was significantly associated with p53 mutation (Supporting Information Table S4). Using a second set of primary EOC for which p53 expression was known, (case material A, in Supporting Information Table S3) we confirmed that p53 overexpression (usually associated to the presence of missense mutations, The Cancer Genome Atlas Research Network, 2011) and high stathmin levels were highly significantly associated (Supporting Information Table S5).

We then analysed by Western blot the expression of stathmin, p53 and DNA-PK (Fig 8B) in a panel of 51 Serous Epithelial Carcinomas (48/51 High Grade) consecutively collected in our Institute between 2010 and 2012 (case material C, in Supporting Information Table S3). Densitometric analyses of the blots was performed to quantify the normalized expression levels of proteins and showed that high levels of stathmin, p53 and DNA-PK were present in 49, 47 and 49% of the cases, respectively. More importantly, stathmin and DNA-PK levels significantly correlated (Spearman *r* = 0.46; 95% C.I. 0.21–0.66 *p* = 0.0005; Fig 8C). Among the 24 tumours overexpressing p53 a positive correlation was found between stathmin and p53 (Spearman *r* = 0.49; 95% C.I. 0.10–0.75 *p* = 0.01; Fig 8D) and between DNA-PK and p53 (Spearman *r* = 0.59; 95% C.I. 0.24–0.80 *p* = 0.002; Fig 8E). Conversely, among the samples that did not overexpress p53 no correlation existed between p53 and stathmin (Spearman *r* = 0.29; *p* = 0.14) or between DNA-PK and p53 (Spearman *r* = 0.09; *p* = 0.65). Finally, using co-immunoprecipitation experiments in four HG-EOC samples, we observed that, in the presence of high stathmin levels, DNA-PK and p53 proteins co-precipitated (Fig 8F), thus confirming in pathological samples from human HG-EOC what we previously observed in *in vitro* and *in vivo* models.

### Combined overexpression of stathmin and p53 represents a marker of poor prognosis in ovarian cancer

The relevance of our findings resided in the possible transferability of their implications to the human patient. To this aim, we verified whether the expression of stathmin and p53 could be used as prognostic biomarker in EOC cancer. We performed immunohistochemical analysis of stathmin in a panel of 128 cases of EOC consecutively collected at the Ospedale Maggiore of Trento, Italy, from 1999 and 2001 (case material A, in Supporting Information Table S3). About 50% of the patients expressed stathmin at high levels. Patients were then stratified based on both intensity of stathmin staining and percentage of positive cells/sample. Cox regression analysis demonstrated that high stathmin expression was significantly associated with decreased overall survival (*p* = 0.021 log-rank test; Fig 8G). In multivariate analysis stathmin represented a significant independent marker of worse prognosis (Supporting Information Table S6).

Finally, we interrogated the TCGA Ovarian Cancer Dataset in which the mRNA expression of 12.624 genes in 517 high grade serous EOC is reported (The Cancer Genome Atlas Research Network, 2011). In this panel stathmin ranks 459 in the top 4% overexpressed genes in serous EOC, compared to normal ovary (mean fold increase 3.5, *p* = 5 × 10^−7^) and DNA-PK, BUB1, CENPA, C21Orf45, FAM64A and NCAPH were similarly overexpressed (Supporting Information Fig S6A). Accordingly, in this dataset NCAPH and CENPA are among the 20 genes most significantly associated with BUB1 expression (Supporting Information Fig S6B). Moreover, stathmin, FAM64A, NCAPH and BUB1 are among the 100 genes most significantly co-expressed with DNA-PK (Supporting Information Fig S6C). Finally, in the TCGA (Supporting Information Fig S7A), in the Anglesio dataset (Supporting Information Fig S7B), that comprises 30 borderline serous ovarian neoplasms and 60 serous adenocarcinomas (Anglesio et al, 2008) and in the Meyniel dataset (Supporting Information Fig S7C), that comprises 44 breast carcinomas and 106 ovarian cancers of different origin (Meyniel et al, 2010), stathmin was co-expressed with BUB1, CENPA, FAM64A and NCAPH, thus further implying that the new axis identified here has a concrete relevance in human EOC.

## DISCUSSION

The present study unveils a new regulatory loop that in HG-EOC is essential for cancer cell survival, especially at work with damaged DNA during mitosis. We provide compelling evidences demonstrating that in HG-EOC cells stathmin is necessary for the proper stability and transcriptional activity of p53^MUT^ by favouring the interaction and the phosphorylation of p53^MUT^ by DNA-PK (Fig 8H). The phosphorylation of p53^MUT^ by DNA-PK not only prevented the binding with MDM2 (Fig 6E) but also could likely favour the binding between p53^MUT^ and its co-transcriptional activators. The clinical relevance of this pathway in human EOC has been clearly demonstrated in three independent sets of EOC samples where high stathmin expression significantly correlated with mutant p53 (Supporting Information Tables S4 and S5 and Fig 8). This correlation has been independently confirmed by a recent report demonstrating that stathmin was overexpressed in 84% of 131 high-grade serous ovarian carcinomas and that its expression was already associated with the p53 proliferative signature, characteristic of premalignant type II EOC (Karst et al, 2011). Thus, while in several types of human cancer stathmin overexpression is typically found in advanced/metastatic disease (reviewed in Belletti & Baldassarre, 2011) and in mice is not required for the onset of sarcoma, skin and bladder carcinomas (D'Andrea et al, 2012), our and others' data pointed to type II HG-EOC as a neoplasia in which stathmin expression is required at early stages of tumour onset, downstream of p53 mutation. This observation is in line with the finding that stathmin knockdown affects the viability of HG-EOC cells expressing the p53^MUT^ protein (Fig 1 and Supporting Information Fig S1) and that stathmin expression is necessary for the survival of mitotic HG-EOC cells (Fig 2).

From a molecular point of view, our data indicated that HG-EOC cell survival required the modulation of a set of p53^MUT^ regulated genes involved in M phase progression and chromosome segregation (Fig 7; Girardini et al, 2011). Therefore, we can depict a scenario in which HG-EOC cells carrying p53^MUT^ are prone to mitotic DNA damage (Fig 2B and Supporting Information Fig S3), likely due to a defective G2 checkpoint (Rausch et al, 2012) and, on the other side, protect themselves from mitotic cell death via the stathmin-DNA-PK-p53^MUT^ axis, necessary for the upregulation of BUB1, CENPA, C21orf45, FAM64A and NCAPH. Indeed this scenario could also explain the fact that p53^MUT^ cells are prone to genomic instability (Oren & Rotter, 2010; Walerych et al, 2012) likely due to alteration of the spindle checkpoint (Gualberto et al, 1998).

The binding of DNA-PK to p53^WT^ is highly controversial (Lakin & Jackson, 1999). Using both overexpressed and endogenous proteins under basal conditions we were not able to detect any significant interaction between p53^WT^ and DNA-PK (Fig 5D and data not shown). These negative results could be either due to the low expression levels of p53^WT^ (TOV21G model in our case) or to post-transcriptional modifications specifically occurring in p53^WT^ protein when expressed in p53 null cells (SKOV-3 model). However, we could observe a direct binding between overexpressed p53^WT^ and DNA-PK, following DNA damage induced by CBDCA (Fig 5E), thus confirming previous observations indicating that this binding is dependent on the induction of DNA damage (Shieh et al, 1999; Woo et al, 2002). In sharp contrast, we showed here that DNA-PK directly binds and phosphorylates two different p53 mutants, thereby modulating their stability and transcriptional activity. Our studies clearly indicated that both p53^R175H^ and p53^R273H^ are able to directly bind DNA-PK and that this interaction relies on stathmin expression, a finding also proved in primary HG-EOC tumours (Fig 8). These conclusions were independently corroborated by the observation that endogenous p53^MUT^ proteins are phosphorylated on S15 after DNA damage and this event does not require the p53-DNA binding, rather is dependent on the tetramerization of the protein (Shieh et al, 1999).

Altogether, our data strongly support that stathmin overexpression is required in HG-EOC cells to overcome M phase failure and drug-induced cell death, particularly following exposure to alkylating agents. These findings imply that high stathmin levels represent a fundamental requisite for HG-EOC cell survival when the genome is prone to accumulate DNA damages, as when p53 is mutated (Muller et al, 2011; Murphy et al, 1999; Oren & Rotter, 2010; Singer et al, 2007).

A recent comprehensive measurement of genomic and epigenomic alterations, annotated in more than 500 serous HG-EOC, demonstrated that p53 is mutated in 95% of the cases and that one third of p53 mutations results in protein truncation (The Cancer Genome Atlas Research Network, 2011). In this panel of samples stathmin ranks 459 in the top 4% overexpressed genes in serous HG-EOC compared to normal ovary (mean fold increase 3.5, *p* = 5 × 10^−7^) and is co-expressed with the p53^MUT^-dependent M phase signature (Supporting Information Figs S6 and S7). These findings strengthen the clinical transferability of our current study. In fact, our data support the hypothesis that in the presence of missense p53 mutations the use of specific drugs that interfere with p53^MUT^ proteins, such as small molecules (Selivanova & Wiman, 2007) or peptide aptamers (Guida et al, 2008), used in combination with platinum compounds and/or taxanes would result in a better disease control. Since p53 truncated protein is usually expressed at very low levels in EOC (The Cancer Genome Atlas Research Network, 2011), we anticipate that the routine evaluation of stathmin and p53 by immunohistochemistry would be sufficient to allow the identification of EOC patients that could benefit from the above mentioned types of treatment. Finally, it is worth mentioning that this type of study could highlight the availability, for selected patients, of new and more appropriate therapeutic options. For instance, based on our results, exploring the activity of the small molecule CC-115, a double inhibitor of mTOR and DNA-PK, currently tested in a phase I clinical trial, could represent a promising strategy for the treatment of selected EOC patients expressing high levels of stathmin and p53 proteins.

## MATERIALS AND METHODS

Detailed experimental procedures and associated references are available online in the Supporting Information section.

### Patient samples

We collected primary ovarian cancer specimens from women at Ospedale Maggiore in Trento and at National Cancer Institute of Milan and at the CRO National Cancer Institute of Aviano. Patients' baseline characteristics are shown in Supporting Information Table S3. High-grade carcinoma patients were classified according to Gilks & Prat (2009).

**The paper explained**

**PROBLEM**

High Grade Epithelial Ovarian Carcinomas (HG-EOCs) represent the most lethal gynaecological cancer due to the late stage at diagnosis and to the onset of drug-resistant recurrences. Type II tumours are characterized by the presence of mutations in the tumour suppressor gene p53, which may as a result be overexpressed. p53 mutations not only disrupt the functions of the wild type protein (loss-of-function) but also confer to the mutant protein new activities (gain-of-function) able to promote tumour progression. In HG-EOCs p53 mutation seems to be the driving oncogenic event. However, several issues on the role(s) of p53 mutation in HG-EOCs onset and progression are still unclear: (1) what is/are the molecular pathway(s) able to stabilize the mutant proteins in transformed cells; (2) what is the function of mutant p53 (p53^MUT^) proteins; (3) how can we explain the clinical evidence suggesting that the overexpression of p53^MUT^ is linked to increased EOCs chemosensitivity.

**RESULTS**

By studying the role of the microtubules destabilizing protein stathmin in EOC, we showed that stathmin co-overexpressed with mutant p53 in primary EOC and that overexpression correlates with shorter patients' survival. We showed that in EOC cells, stathmin is required to sustain the expression and activity of mutant p53 by favouring mutant p53 and DNA-dependent protein kinase (DNA-PK_CS_) interaction and subsequent phosphorylation of S15 and S34 on p53 by DNAPK_CS_. This phosphorylation, in turn, regulates mutant p53 protein stability and transcriptional activity, necessary for progression through mitosis and survival of EOC cells especially in the presence of damaged DNA, which is frequently found in p53^MUT^ bearing cells.

**IMPACT**

This study highlights a crucial role for stathmin in the control of mutant p53 protein stability and transcriptional activity in EOC. Accordingly, stathmin is required for the growth and survival of EOC cells carrying a mutant p53 protein via the modulation of p53^MUT^/DNA-PK_CS_ interaction.

From a clinical point of view, our results could have an immediate translational relevance since the evaluation of stathmin and p53 expression could be used to identify EOC patients with poor prognosis who may benefit from more aggressive/alternative therapies acting in M phase. Moreover, our data suggest that the stathmin/DNA-PK_CS_/p53^MUT^ axis represents a new promising target for more active therapies in EOC.

### Statistical analysis

Statistical significance of the results was determined by using the paired and unpaired Student's *t*-test and the GraphPad PRISM software, as indicated. Additional details are provided in Supporting information.

### Cell culture

MDAH 2774 (CRL-10303), TOV-112D (CRL-11731), SKOV-3 (HTB-77), OVCAR-5 (NIH) and TOV-21G (CRL-11730) cells were obtained from ATCC and maintained in RPMI-1640 medium (Sigma–Aldrich Co.) supplemented with 10% heat-inactivated FBS. HEK 293 (ATCC CRL-1573), used to produce adenoviral particles, and 293FT cells (Invitrogen, Inc.), used for lentivirus production, were grown in DMEM supplemented with 10% heat-inactivated FBS (Sigma–Aldrich Co.). All cell lines were authenticated by BMR Genomics srl, Padova, Italia, on January 2012 according Cell ID™ System (Promega) protocol and using Genemapper ID Ver 3.2.1 to identify DNA STR profiles.

### Compounds and drug treatments

Paclitaxel (TAXOL®) and Carboplatin (CBDCA) (TEVA Italia) were used for *in vitro* and/or *in vivo* experiments. The specific DNA-PK inhibitor NU7441 (Leahy et al, 2004; TOCRIS Biosciences) was dissolved in DMSO as 5 mM stock and stored at −20°C.

### Vectors, transfections and viruses

pCMV empty, pCMV/Neo-p53WT, pCMV/Neo-p53R175H or pCMV/Neo-p53R273H vectors were obtained from Addgene, Inc. (Cambridge, Massachusetts) and transfected in SKOV-3 cells using FuGENE HD Transfection Reagent (Roche Applied Science, Indianapolis, Indiana). Stable p53^MUT^ expressing clones were selected by culturing cells with G418 (0.5 mg/mL). For lentiviral production, 293FT cells (Invitrogen, Carlsbad, California) were co-transfected, using a standard calcium phosphate precipitation, with the lentiviral based shRNA constructs (pLKO) and lentiviral vectors pLP1, pLP2 and pVSV-G (Invitrogen). Seventy-two hours after transfection, medium containing viral particles was used to transduce EOC cell lines. pLKO vectors encoding for control, stathmin and DNA-PK shRNAs (Moffat et al, 2006) were purchased from Sigma–Aldrich Co. (St. Louis, Missouri). Stable knockdown clones were selected by culturing cells in puromycin (1 µg/mL) containing medium.

Adenovirus production, titration and characterization were performed as previously described (Schiappacassi et al, 2008). For *in vivo* experiments, adenoviruses were purified according to manufacturer's indications (Adeno-X Virus purification kit, BD Biosciences, San Jose, California).

### *In vitro* cell proliferation assay and clonogenic assay

Cells (1000/well), transduced with control, stathmin or DNA-PK sh-RNAs, were seeded in quadruplicate in 96-well plates and treated or not with increasing concentrations of indicated compounds, for designated times. Cell proliferation was determined 48 h after treatment, by MTS assay using the CellTiter 96 AQueous cell proliferation assay kit (Promega).

For growth curve analyses, EOC cells stable transduced with sh-ctrl or sh-STM were cultured in 6-well plates. Viable cells were counted daily in triplicate for 5 days, by trypan-blue dye exclusion method. All experiments were repeated at least three times.

For colony assay, cells transduced with control or stathmin sh-RNAs were trypsinized, counted and seeded at density 1000 or 2000 cell/100 mm dish and incubated in complete growth medium. Two weeks later plates were stained with crystal violet and colonies were counted.

### RNA extraction, RT-PCR and real-time PCR

Total RNA from EOC transduced with Ad sh-STM or Ad sh-ctrl cells was extracted using the RNeasy kit (Qiagen). Two micrograms of total RNA was retrotranscripted using AMV Reverse Transcriptase and random hexamers according to the provider's instruction (Promega); 1/20 of the obtained cDNAs were then amplified using primers for the human FAM64A, CENPA, BUB1, NCAPH, C21ORF45 (Girardini et al, 2011), DNA-PK sequence or with primers for human GAPDH and SDAH mRNA. Primers used were: human DNA-PK (forward 5′-CATGGAAGAAGATCCCCAGA-3′, reverse 5′-TGGGCACACCACTTTAACAA-3′); human GAPDH (forward 5′-GAAGGTGAAGGTCGGAGTC-3′, reverse 5′-GAAGATGGTGATGGGATTTC-3′); human SDHA (forward 5′-AGAAGCCCTTTGAGGAGCA-3′, reverse 5′-CGATTACGGGTCTATATTCCAG-3′). Quantitative Real-Time PCR analyses were performed using the SoFast EvaGreen Supermix (BioRad Laboratories).

### Tumour xenograft studies

All animal experiments were performed in strict accordance with institutional guidelines and were approved by CRO Ethical Committee for Animal Experimentation (CESA). For pathological analyses of xenograft, explanted tumours were fixed in formalin and after paraffin inclusion stained with Hematoxylin & Eosin. To evaluate morphology and mitotic index of the different tumour specimens, an expert pathologist examined all samples in a double blind fashion.

## Author contributions

GB conceived and design the project; MSo, MSc, BB and GB analysed the data and wrote the paper; MSo, MSc, SL, ADA, FL, ML, BP, SDA performed the experiments; GDS, MN contributed new reagents/analytic tools; BB, GDS, DM, SC, AC analysed the data and provided important intellectual content and advices in drafting and/or critically revising the article; GG, MBag, DM, MBar, BV, SC, LM, VC provided patient's data and IHC analysis.

## For more information

ONCOMINE:

https://www.oncomine.org/resource/login.html

Allow comparing gene expression profiles between normal and cancer samples, or between different molecular, pathological and clinical subtypes of cancer.

Oncomine Research Edition is free to the academic and nonprofit cancer research communities.

Addgene:

http://www.addgene.org/

Addgene is a non-profit plasmid repository dedicated to helping scientists around the world share high-quality plasmids.

p53 Databases:

http://p53.free.fr/Database/p53_database.html

http://p53.fr/

Provide updated information of the p53 tumour suppressor genes and a complete database of p53 mutations in human cancer

## Notes

As a service to our authors and readers, this journal provides supporting information supplied by the authors. Such materials are peer reviewed and may be re-organized for online delivery, but are not copy-edited or typeset. Technical support issues arising from supporting information (other than missing files) should be addressed to the authors.
