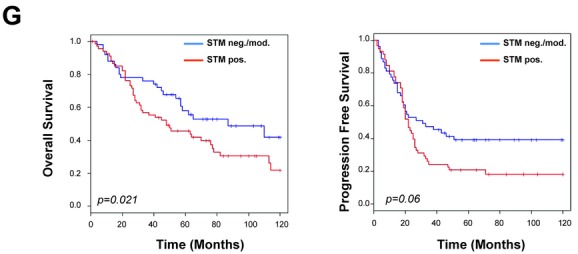# Stathmin regulates mutant p53 stability and transcriptional activity in ovarian cancer

**DOI:** 10.1002/emmm.201470020

**Published:** 2014-02-07

**Authors:** Maura Sonego, Monica Schiappacassi, Sara Lovisa, Alessandra Dall'Acqua, Marina Bagnoli, Francesca Lovat, Massimo Libra, Sara D'Andrea, Vincenzo Canzonieri, Loredana Militello, Marco Napoli, Giorgio Giorda, Barbara Pivetta, Delia Mezzanzanica, Mattia Barbareschi, Barbara Valeri, Silvana Canevari, Alfonso Colombatti, Barbara Belletti, Giannino Del Sal, Gustavo Baldassarre

The authors of the above research article have informed the journal that an error occurred during assembly of the graphs shown in Figure 8G. The new Figure 8G below contains the correct graphs and replaces panel G of the original figure. The original statistical analyses and the *P*-values obtained remain valid. In any case, this mistake does not affect the results and conclusions of the paper.

**Figure 8G:**