# Supplementary material for: Stathmin regulates mutant p53 stability and transcriptional activity in ovarian cancer
Source: EMBO Mol Med. 2013 Apr 22;5(5):707–22. doi: 10.1002/emmm.201201504 (PMC3662314; doi:10.1002/emmm.201201504)
Supplement: Supplementary file 1 [file emmm0005-0707-sd1.pdf]

Stathmin regulates mutant p53 stability and transcriptional activity in ovarian cancer

Maura Sonego, Monica Schiappacassi, Sara Lovisa, Alessandra Dall'Acqua, Marina Bagnoli, Francesca Lovat Massimo Libra, Sara D'Andrea, Vincenzo Canzonieri, Loredana Militello, Marco Napoli, Giorgio Giorda, Barbara Pivetta, Delia Mezzanzanica, Mattia Barbareschi, Barbara Valeri, Silvana Canevari, Alfonso Colombatti, Barbara Belletti, Giannino Del Sal and Gustavo Baldassarre

Corresponding author: Gustavo Baldassarre, Centro di Riferimento Oncologico

Review timeline:

Submission date:	24 April 2012
Editorial Decision:	06 June 2012
Appeal:	21 December 2012
Editorial Decision:	08 February 2013
Revision received:	18 February 2013
Accepted:	20 February 2013

Transaction Report:

(Note: With the exception of the correction of typographical or spelling errors that could be a source of ambiguity, letters and reports are not edited. The original formatting of letters and referee reports may not be reflected in this compilation.)

Editor: Céline Carret

1st Editorial Decision 06 June 2012

Thank you for the submission of your manuscript to our editorial office. We have now received feedback from 2 out of 3 reviewers whom we asked to evaluate your manuscript. Given that both reviewers provide very similar concerns, we prefer to make a decision now in order to avoid further delay in the process.

As you will see from the enclosed reports, neither referee provide enthusiastic support for publication of the manuscript in EMBO Molecular Medicine. Although they both seem to agree that the study provides some clinical relevance, Referee #1 deplores the limited novelty restricted only to the positive regulation of mutant p53 by stathmin. Yet, despite Referee #2 being more moderate, both referees feel that the mechanistic insights required to substantiate this part of the work are not sufficient for publication.

Given these negative opinions and the fact that the EMBO Molecular Medicine can only afford to accept papers which receive enthusiastic support from a majority of referees, I am afraid that I see little choice but to return the manuscript to you at this point with the decision that we cannot offer to publish it.

I am sorry that I could not bring better news this time and hope that the referees' comments are helpful in your continued work in this area.

***** Reviewer's comments *****

Referee #1 (Comments on Novelty/Model System):

No model organism - just cultured cells (+ human tumor data)

Referee #1 (Other Remarks):

In this paper, the authors report that stathmin expression is positively regulated by mutant p53 in type II ovarian cancer cells, that silencing of stathmin by siRNA inhibits the proliferation and promotes the death of those cells, and that this death is further increased by a variety of chemotherapy agents. They further report that stathmin expression positively regulates mutant p53 protein stability, and this is mediated via the ability of stathmin to induce the expression of DNA-PK and the subsequent phosphorylation of mutant p53. Evidence is provided that high stathmin and mutant p53 protect cells with damaged genomes against caspase-independent mitotic cell death. Finally, analysis of ovarian cancer patient data reveals a positive correlation between mutant p53, stathmin and DNA-PK, as well as with several other genes encoding proteins that are involved in M phase progression control.

The findings reported in this study are interesting and of potential clinical importance, albeit not as novel as suggested by the authors. At least some of the main conclusions have already been described for other types of cancer cell lines and tumors. For instance, Alli et al (Oncogene 2007, not cited by the authors) already reported that silencing of stathmin decreased cell proliferation and viability in cancer cells harboring mutant p53. Carney and Cassimeris (2010, not cited by the authors) also reported that stathmin depletion caused death of cells lacking wild type p53. Likewise, in page 7, the authors present as surprising their observation that stathmin depletion sensitizes mutant p53 tumor cells to carboplatin. However, Singer et al (2007) already reported the same for cisplatin: hence, this is not surprising at all. Likewise, positive correlation between stathmin and p53 mutation, as well as between high statmin and bad prognosis, was reported in several studies (e.g. Yuan et al, J. Pathol 2006; cited).

Hence, the most novel aspect of this study is the other side of the coin, namely the positive regulation of mutant p53 by stathmin. However, this aspect lacks sufficient mechanistic insights, and should be strengthened.

Major comments:

1. The authors should obtain mechanistic information on how stathmin induces DNA-PK.
2. Is stathmin reducing the extent of mutant p53 ubiquitination? Is its effect dependent on MDM2? Is the effect of stathmin silencing on mutant p53 levels reversed by treatment of the cells with proteasome inhibitors?
3. Page 9-10. It will be important to show that stathmin does not stabilize mutant p53 carrying additional mutations in S15 and S37.
4. Fig. 6B. The conclusion that depletion of stathmin reduces the binding of mutant p53 to DNA-PK is questionable, because it could simply be due to the reduction in the total amounts of mutant p53. The authors should show the input levels of each protein in the extracts, and normalize the signal to the input amount of p53. Alternatively, they should run separately a small fraction of the IP and blot it for p53, and normalize the DNA-PK signal to the signal of that p53 blot. In the IP shown in Fig. 6B the p53 lanes are heavily overloaded, and therefore they are probably not representing the true differences in p53 amounts in the 2 lanes. Only if this is done properly will the authors be able to support their claim, which is important for delineating the molecular mechanism. And, speaking of Fig. 6B: why was the IP done with DNA-PK antibodies in Fig. 6A but with p53 antibodies in Fig. 6B? This lack of consistency is not helpful.
5. Fig. 8. To support the claim that the effect of stathmin is mediated via Bub1, the authors should perform a rescue experiment and show that Bub1 overexpression can relieve, at least partially, the effects of stathmin silencing.

Minor comments:

1. In Fig. 3A,B, knockdown of stathmin seems to actually increase tumor number when p53 is wild type. Is that significant? If so, the authors should comment on it in the text.
2. The English grammar and syntax should be improved.
3. Page 7: Fig. 4A is discussed instead of 4B.
4. Fig. 6D. The upper panel is very "dirty" and not suitable for a high quality journal. The authors should attempt to obtain a more presentable result.

Referee #3 (Other Remarks):

This manuscript is focused on the role of stathmin in ovarian cancer, expressing mutant p53. They show in a vast analysis of data obtained from human ovarian cancer that high stathmin levels are associated with mutations in p53 and with poor prognosis. Furthermore, they showed that knocking down stathmin decreases the survival and resistance to chemotherapy of only mutant p53 expressing cells. Finally, they suggest that the mechanism underlying this phenotype is interfering with M phase by altering the expression of M phase genes.
These data suggest an important molecular link associated with poor prognosis of ovarian cancer, and it is supported by clinical data, mouse model and in vitro studies.
Nevertheless, the suggested mechanism underlying this important phenotype needs to be strengthen. Mainly, since the authors focused their conclusions on the p53-stathmin loop, and specified it to mutant p53, the authors should better examine and discuss the existence of mutant p53 and stathmin positive loop, and the role of wild-type p53 in this pathway. In general, the authors should moderate their conclusions, and to restrict them to the observed results. Altogether, I suggest to accept this manuscript for publication in EMBO Molecular Medicine following manuscript revision, relating to the following comments:

1) Figure 2A: Since the levels of the normalizing protein used, Vincullin, are not comparable, in order to compare stathmin expression in the various cell lines, the authors should quantify the western blot bands and present stathmin levels in respect to Vincullin.
2) The authors suggest a feedback loop between stathmin and p53. The evidences leading to this conclusion should be strengthen. For example, in Figure 2A it seems that there is not correlation between mutant p53 expression and higher stathmin levels. In addition, in Figure 4 stathmin levels do not seem higher in mutant p53 expressing MEFs, and mutant p53 levels are not reduced due to stathmin knock-down. The authors should consider the possibility that mutant p53 and stathmin are both important but independent events, and elaborate on these less consistent results in the discussion section.
3) Although the author claim that stathmin knock down does not effect the viability of wild-type p53 cells (page 6 line 14), figure S1F show a slight reduction in wild-type p53 cells viability (indeed, less than mutant p53 cells). Therefore, in order to fully cover the molecular mechanism underlying stathmin-p53 crosstalk, the authors should show more data regarding wild-type p53 and its role related to stathmin.
4) Figures 5D and 6D: DNA-PKcs seems to hardly bind to mutant p53 R273H (although mentioned differently in the text: page 9 line 23). This is in accordance to the higher viability of MDAH compare to TOV-112D following DNA-PKc knock-down. This raises the possibility that DNA-PKcs-mutant p53 binding is necessary for survival. The authors should relate to it in the discussion section.
5) The authors should better explain the lowering and double band of stathmin observed in the shake off (mitotic) cells.
6) The authors should explain the aim of nocodazole treatment and show the not treated cells.
7) Figure S6A: the authors should indicate which cells are displayed.
8) Writing:
- Page 7 line 3-5: from data relating to this conclusion, the authors can conclude that stathmin regulate tumor/cell growth. However they cannot conclude yet that stathmin is mutant p53 target.
- Page 7 line 20 and page 8 line 2, "Fig 4A" should be "Fig4B".
- Figure 4A (lower panel): x axis title is missing

Appeal 21 December 2012

Thank you for your e-mail of last June 6 on the manuscript EMM-2012-01504 entitled "Stathmin is necessary for the survival of ovarian cancer cells expressing p53MUT protein" by Maura Sonego, Monica Schiappacassi, Sara Lovisa, Alessandra Dall'Acqua, Marina Bagnoli, Francesca Lovat, Massimo Libra, Barbara Belletti, Ilenia Pellizzari, Vincenzo Canzonieri, Giorgio Giorda, Marco Napoli, Delia Mezzanzanica, Mattia Barbareschi, Barbara Valeri, Alfonso Colombatti, Silvana Canevari, Giannino Del Sal and Gustavo Baldassarre submitted for publication as a research article in EMBO Molecular Medicine.

We read with interest the Reviewers' comments and we think they helped us to significantly improve our manuscript.

As you will see from the attached point by point response to the Reviewers' comments we worked hard in these months to address their criticisms and we think that we responded in full to their criticisms.

Moreover, we better addressed the role of stathmin in primary epithelial ovarian cancer and we now also proved in vivo what we previously observed in vitro (i.e. that stathmin expression influences the binding of DNA-PK to p53MUT).

We therefore strongly believe that our work merits to be considered for a revision in EMBO Molecular Medicine, and, if you agree, we are willing to submit a revised version of the manuscript for Reviewers' evaluation

Point-by-Point Response:

Referee #1 (Comments on Novelty/Model System):

No model organism - just cultured cells (+ human tumor data)

We partially disagree with Reviewer 1 on this point since, although based on xenograft of human cell lines, four *in vivo* models have been used in our studies. In all these experiments we confirmed our observation.

However, in the revised version we strengthen the *in vivo* significance of our results both in mice and in primary EOCs confirming the clinical relevance of our results (new figure 8).

Referee #1 (Other Remarks):

In this paper, the authors report that stathmin expression is positively regulated by mutant p53 in type II ovarian cancer cells, that silencing of stathmin by siRNA inhibits the proliferation and promotes the death of those cells, and that this death is further increased by a variety of chemotherapy agents. They further report that stathmin expression positively regulates mutant p53 protein stability, and this is mediated via the ability of stathmin to induce the expression of DNA-PK and the subsequent phosphorylation of mutant p53. Evidence is provided that high stathmin and mutant p53 protect cells with damaged genomes against caspase-independent mitotic cell death. Finally, analysis of ovarian cancer patient data reveals a positive correlation between mutant p53, stathmin and DNA-PK, as well as with several other genes encoding proteins that are involved in M phase progression control.

The findings reported in this study are interesting and of potential clinical importance, albeit not as novel as suggested by the authors. At least some of the main conclusions have already been described for other types of cancer cell lines and tumors. For instance, Alli et al (Oncogene 2007, not cited by the authors) already reported that silencing of stathmin decreased cell proliferation and viability in cancer cells harboring mutant p53. Carney and Cassimeris (2010, not cited by the authors) also reported that stathmin depletion caused death of cells lacking wild type p53. Likewise, in page 7, the authors present as surprising their observation that stathmin depletion sensitizes mutant p53 tumor cells to carboplatin. However, Singer et al (2007) already reported the same for cisplatin: hence, this is not surprising at all. Likewise, positive correlation between stathmin and

p53 mutation, as well as between high statmin and bad prognosis, was reported in several studies (e.g. Yuan et al, J. Pathol 2006; cited).

Hence, the most novel aspect of this study is the other side of the coin, namely the positive regulation of mutant p53 by stathmin. However, this aspect lacks sufficient mechanistic insights, and should be strengthened.

We thanks this Reviewer for finding "*interesting and of potential clinical importance"* our data. We agree with this Reviewer that others reported some of our observations, although in different and unrelated models. However, accordingly with His/Her suggestion we significantly improved the mechanistic part of the work and we are confident that in the present for He/She will find our work suitable for publication in EMM.

Major comments:

1. The authors should obtain mechanistic information on how stathmin induces DNA-PK.

1. We investigated this point and we discovered that stathmin regulates DNA-PK expression at transcriptional level, likely due to its activity on MT stability, as evidenced by the use of low doses of Taxol or Nocodazole. We will provide these information as confidential data to the reviewers but we prefer not to include them in the present manuscript since they need a more detailed study.

2. *Is stathmin reducing the extent of mutant p53 ubiquitination? Is its effect dependent on MDM2? Is the effect of stathmin silencing on mutant p53 levels reversed by treatment of the cells with proteasome inhibitors?*

2. We more deeply investigated this point showing that stathmin knock-down favors the binding between p53MUT and MDM2 in a phosphorylation-dependent manner (new Figure 6).

3. *Page 9-10. It will be important to show that stathmin does not stabilize mutant p53 carrying additional mutations in S15 and S37.*

3. We generated the p53R175H and the p53R273H mutants carrying also the S15A and the S37A mutations and verified that stathmin knock-down had no effect on their stability and on their binding to MDM2 (new figure 6).

4. Fig. 6B. The conclusion that depletion of stathmin reduces the binding of mutant p53 to DNA-PK is questionable, because it could simply be due to the reduction in the total amounts of mutant p53. The authors should show the input levels of each protein in the extracts, and normalize the signal to the input amount of p53. Alternatively, they should run separately a small fraction of the IP and blot it for p53, and normalize the DNA-PK signal to the signal of that p53 blot. In the IP shown in Fig. 6B the p53 lanes are heavily overloaded, and therefore they are probably not representing the true differences in p53 amounts in the 2 lanes. Only if this is done properly will the authors be able to support their claim, which is important for delineating the molecular mechanism. And, speaking of Fig. 6B: why was the IP done with DNA-PK antibodies in Fig. 6A but with p53 antibodies in Fig. 6B? This lack of consistency is not helpful.

4. Our hypothesis was that stathmin influenced p53MUT stability by decreasing DNA-PK expression. We think that our experiments proved this point. In the new figure 5A we demonstrated that the binding between DNA-PK and p53MUT endogenous proteins is readily observable by immunoprecipitating (IP) either p53 or DNA-PK. Then, we alternatively used the IP for DNA-PK or for p53 not for inconsistency but, conversely, to strengthen our point. We have now better explained this point in the manuscript in order to more precisely clarify the significance of our results. Importantly, we proved (new figure 8F) that the binding between p53MUT and stathmin is observable also in primary EOC and correlates with stathmin expression, confirming *in vivo* what discovered *in vitro*.

5. Fig. 8. To support the claim that the effect of stathmin is mediated via Bub1, the authors should perform a rescue experiment and show that Bub1 overexpression can relieve, at least partially, the effects of stathmin silencing.

5. We probably did not explain clearly enough our data. We did not claim (and do not believe) that Bub1 alone could mediate the effects of stathmin. Conversely, we think that is the whole transcriptional program induced by p53MUT necessary for the survival of mitotic cells. We better clarified this point in the new version of the manuscript.

Minor comments:

1. In Fig. 3A,B, knockdown of stathmin seems to actually increase tumor number when p53 is wild type. Is that significant? If so, the authors should comment on it in the text.

1. The differences in tumor growth between control- and stathmin-silenced cells observed in p53 null cells (both OVCAR5 and SKOV3) were not significant. Regarding p53WT cells (TOV21G) we could not test the effects of stathmin *in vivo* since these cells grew at very little extent in nude mice.

2. The English grammar and syntax should be improved.

2. We now carefully edited the text with the help of an English mother-tongue editor.

3. Page 7: Fig. 4A is discussed instead of 4B.

3. We apologize for this inaccuracy. We now corrected the mistake.

4. Fig. 6D. The upper panel is very "dirty" and not suitable for a high quality journal. The authors should attempt to obtain a more presentable result.

4. We tried to obtain a better image of this IP (new figure 5D), but it has to be considered that a high molecular weight protein, such as DNA-PK (more than 300KDa), is often difficult to resolve in SDS-PAGE and transfer to the nitrocellulose.

Referee #3 (Other Remarks):

This manuscript is focused on the role of stathmin in ovarian cancer, expressing mutant p53. They show in a vast analysis of data obtained from human ovarian cancer that high stathmin levels are associated with mutations in p53 and with poor prognosis. Furthermore, they showed that knocking down stathmin decreases the survival and resistance to chemotherapy of only mutant p53 expressing cells. Finally, they suggest that the mechanism underlying this phenotype is interfering with M phase by altering the expression of M phase genes.

These data suggest an important molecular link associated with poor prognosis of ovarian cancer, and it is supported by clinical data, mouse model and in vitro studies.

Nevertheless, the suggested mechanism underlying this important phenotype needs to be strengthened. Mainly, since the authors focused their conclusions on the p53-stathmin loop, and specified it to mutant p53, the authors should better examine and discuss the existence of mutant p53 and stathmin positive loop, and the role of wild-type p53 in this pathway. In general, the authors should moderate their conclusions, and to restrict them to the observed results. Altogether, I suggest to accept this manuscript for publication in EMBO Molecular Medicine following manuscript revision, relating to the following comments:

We thank Reviewer #3 for finding of interest our manuscript and for His/Her suggestion to "*to accept this manuscript for publication in EMBO Molecular Medicine following manuscript revision*". We believe that in this revised version we strengthen our results according to Reviewers' comments. We are confident that in the present form He/She will find our work suitable for publication in EMM.

1) *Figure 2A: Since the levels of the normalizing protein used, Vincullin, are not comparable, in order to compare stathmin expression in the various cell lines, the authors should quantify the western blot bands and present stathmin levels in respect to Vincullin.*

1) We provided the requested normalization under the blot in the new figure 1A.

2) The authors suggest a feedback loop between stathmin and p53. The evidences leading to this conclusion should be strengthened. For example, in Figure 2A it seems that there is not correlation between mutant p53 expression and higher stathmin levels. In addition, in Figure 4 stathmin levels do not seem higher in mutant p53 expressing MEFs, and mutant p53 levels are not reduced due to stathmin knock-down. The authors should consider the possibility that mutant p53 and stathmin are both important but independent events, and elaborate on these less consistent results in the discussion section.

2) We agreed with the Reviewer' comments on this point. To better clarify our results we focused our attention on the role of stathmin and p53MUT in ovarian cancer cells and, more specifically, on the role of stathmin in the regulation of p53 stability (that represents the major novelty of our work).

In the present version of the manuscript we do not discuss any more the role of p53 in the control of stathmin expression (already published by others) and the role of stathmin and p53MUT in primary MEF.

To strengthen our results on the relationship between stathmin and p53MUT stability we provided a clearer blot of mutant p53 proteins in SKOV3 transfected cells (new figure 3B old figure 4). Moreover we generated the p53R175H and p53R273H mutants carrying the S15A and S37A substitution (new figure 6). Using these mutants we confirmed that stathmin regulates p53MUT stability in a S15/S37-phosphorylation dependent manner (new Figure 6).

Finally, according to Reviewer's suggestion, we better elaborated the discussion section.

3*) Although the author claim that stathmin knock down does not effect the viability of wild-type p53 cells (page 6 line 14), figure S1F show a slight reduction in wild-type p53 cells viability (indeed, less than mutant p53 cells). Therefore, in order to fully cover the molecular mechanism underlying stathmin-p53 crosstalk, the authors should show more data regarding wild-type p53 and its role related to stathmin.*

3. The results on TOV21G cells reported in the new figure S1G demonstrated that the differences in cell survival between control- and stathmin-silenced cells were not statistically significant. We thus preferred to concentrate our efforts on the relationship between stathmin and p53MUT proteins.

4) Figures 5D and 6D: DNA-PKcs seems to hardly bind to mutant p53 R273H (although mentioned differently in the text: page 9 line 23). This is in accordance to the higher viability of MDAH compare to TOV-112D following DNA-PKc knock-down. This raises the possibility that DNA-PKcs-mutant p53 binding is necessary for survival. The authors should relate to it in the discussion section.

4) We agree with the Reviewer and we better discussed this point in the text.

5) The authors should better explain the lowering and double band of stathmin observed in the shake off (mitotic) cells.

5) Stathmin is heavily phosphorylated during mitosis to allow spindle formation. The higher molecular weight band of the blot represents the tetra-phosphorylated (pS16, pS25, pS38 and pS63) form of the protein. Thus, the appearance of higher molecular weight bands was expected and confirmed that we were analyzing extracts from mitotic cells.

6) The authors should explain the aim of nocodazole treatment and show the not treated cells.

6) Nocodazole treatment was used to synchronize cells in M Phase, in order to obtain a substantial number of mitotic cells to be analyzed. Untreated cells were shown; in old figure 8A (new figure 2B) untreated cells were the lane marked as "async". (asynchronous). In old figure 8G (new Figure 7E) untreated cells were reported in the first lane.

7) Figure S6A: the authors should indicate which cells are displayed.

7) We corrected the figure legend accordingly.

8) Writing:

Page 7 line 3-5: from data relating to this conclusion, the authors can conclude that stathmin regulate tumor/cell growth. However they cannot conclude yet that stathmin is mutant p53 target.

Page 7 line 20 and page 8 line 2, "Fig 4A" should be "Fig4B".

Figure 4A (lower panel): x axis title is missing

8) We regret for the inaccuracies present in the old version of the manuscript. We have now carefully edited the text and corrected the mistakes, also according to the Reviewer's remarks.

2nd Editorial Decision 08 February 2013

Thank you for the submission of your revised manuscript to EMBO Molecular Medicine. We have now received the enclosed reports from the referees that were asked to re-assess it.

As you will see the reviewers are now supportive and I am pleased to inform you that we will be able to accept your manuscript if you can provide, and add to the manuscript, the data requested by Referee #1.

I look forward to seeing a revised form of your manuscript as soon as possible.

***** Reviewer's comments *****

Referee #1 (General Remarks):

Although not all my concerns have been fully addressed, the paper as a whole is now significantly improved. I have only one remaining request: in response to my major comment 1, the authors refer to confidential mechanistic data on the regulation of DNA-PK transcription by stathmin, which they did not include because they need a more detailed study. It is OK not to include the detailed mechanistic data, but it is essential to at least include a panel showing the effect of stathmin on DNA-PK mRNA levels. This should be done next to Fig. 5B, which shows the effect of stathmin on DNA-PK protein levels.
Minor comment: page 19, line 2: the present study (word missing).

Referee #3 (Comments on Novelty/Model System):

The authors have dealt adequately with my concerns. By focusing on the regulation of mutant p53 stability by stathmin, and adding new experiments they improved the manuscript. Therefore, I find the manuscript suitable for publication.

1st Revision - authors' response 18 February 2013

Referee #1 (General Remarks):

Although not all my concerns have been fully addressed, the paper as a whole is now significantly improved.

We thank Referee 1 for finding our work significantly improved and for the suggestions He/She provided us to reach this objective.

I have only one remaining request: in response to my major comment 1, the authors refer to confidential mechanistic data on the regulation of DNA-PK transcription by stathmin, which they did not include because they need a more detailed study. It is OK not to include the detailed mechanistic data, but it is essential to at least include a panel showing the effect of stathmin on DNA-PK mRNA levels. This should be done next to Fig. 5B, which shows the effect of stathmin on DNA-PK protein levels.

As requested, in the new version of the manuscript (New Figure 4C) we added the qRT-PCR data showing that stathmin knockdown decreased DNA-PK mRNA levels in all EOC cells analyzed.

Minor comment: page 19, line 2: the present study (word missing).

We rephrased the sentence.

Referee #3 (Comments on Novelty/Model System):

The authors have dealt adequately with my concerns. By focusing on the regulation of mutant p53 stability by stathmin, and adding new experiments they improved the manuscript. Therefore, I find the manuscript suitable for publication.

We thank Referee 3 for finding our manuscript suitable for publication in EMBO Molecular Medicine and for helping us, with His/Her suggestions, to improve our work.
