# Supplementary figures and images for: Stathmin regulates mutant p53 stability and transcriptional activity in ovarian cancer
Source: EMBO Mol Med. 2013 Apr 22;5(5):707–22. doi: 10.1002/emmm.201201504 (PMC3662314; doi:10.1002/emmm.201201504)

Figure 1

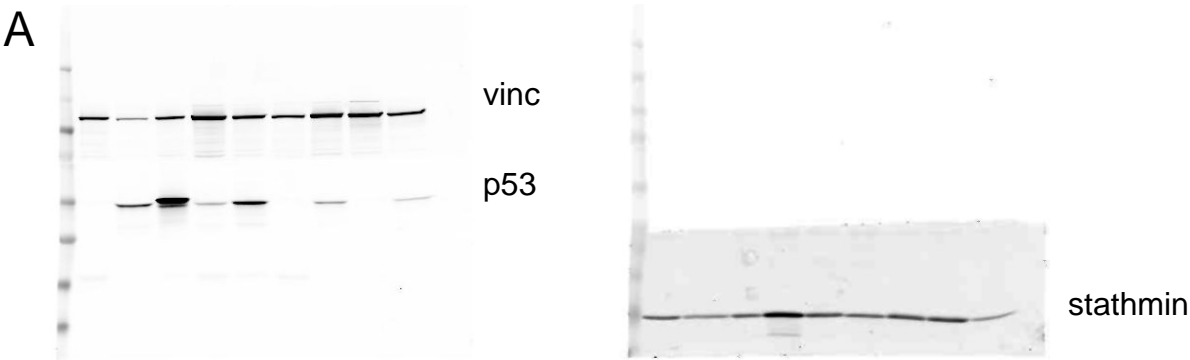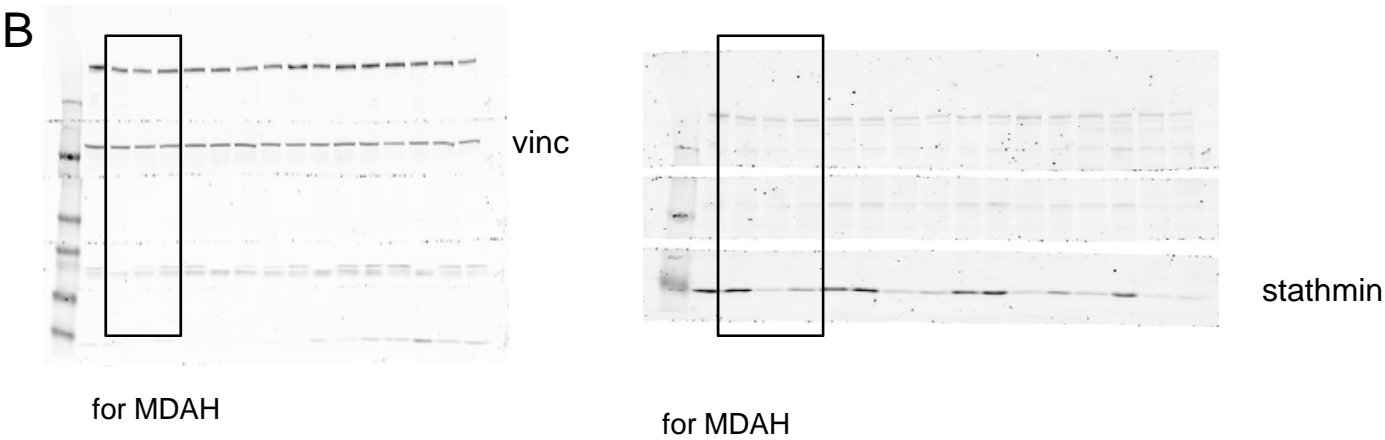

Figure 1

B

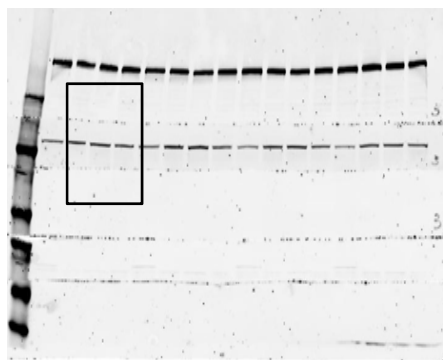

vinc

for OVCAR5

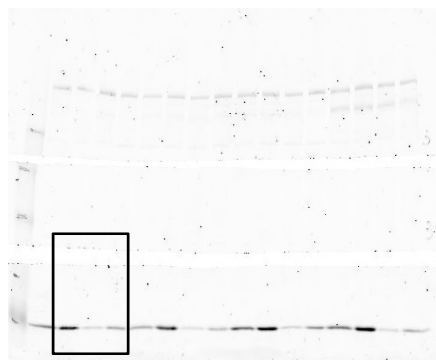

stathmin

for OVCAR5

B

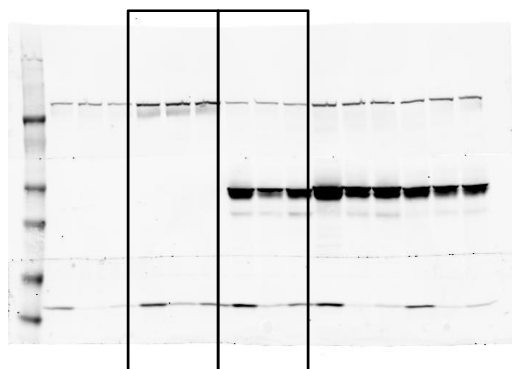

vinc

stathmin

for SKOV3 and TOV112D

Supplement: Supplementary file 2 [file emmm0005-0707-sd2.pdf]

Figure 2

A for MDAH

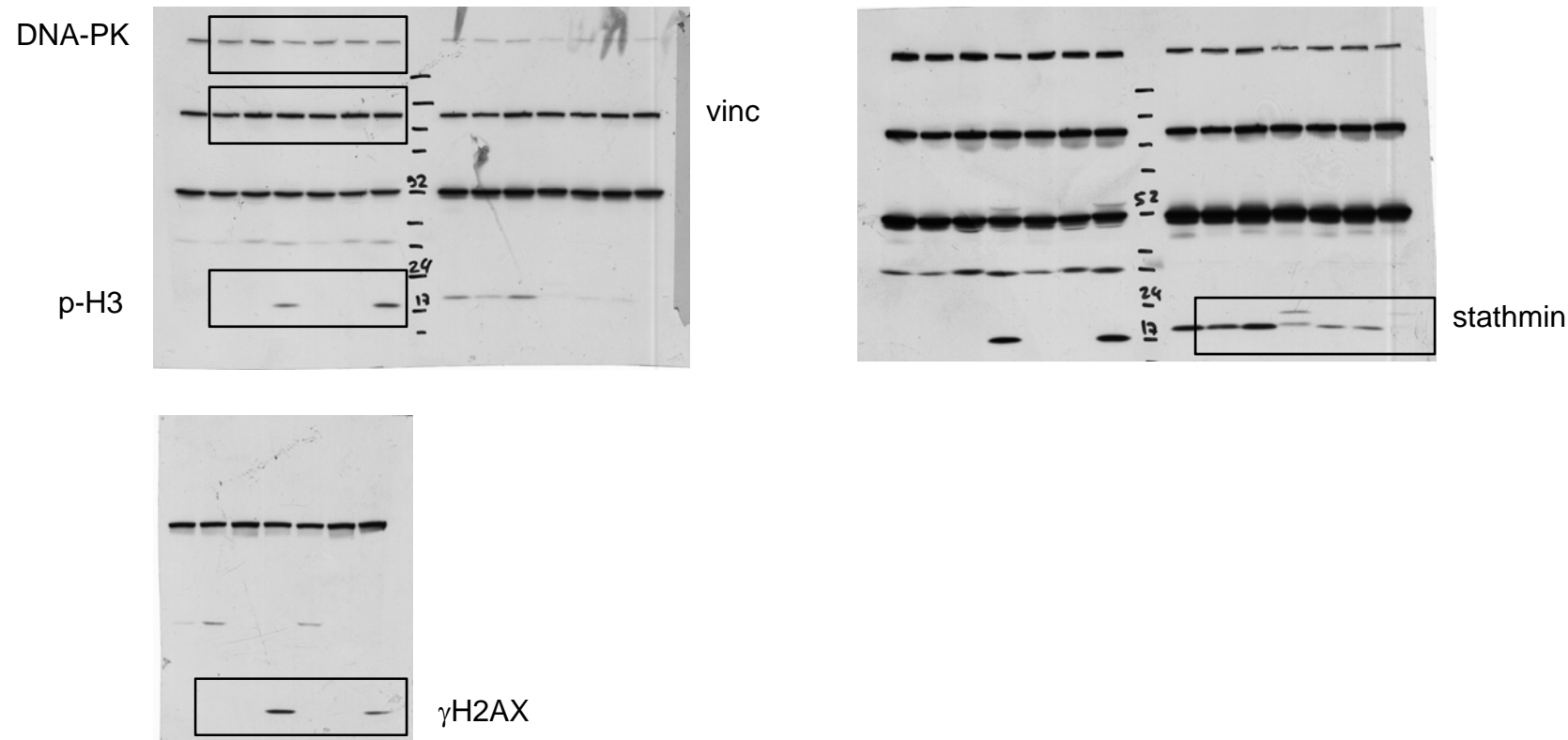

Figure 2

for SKOV3

A

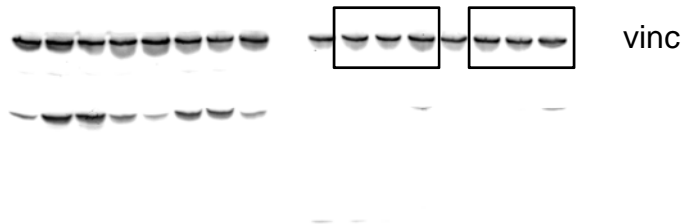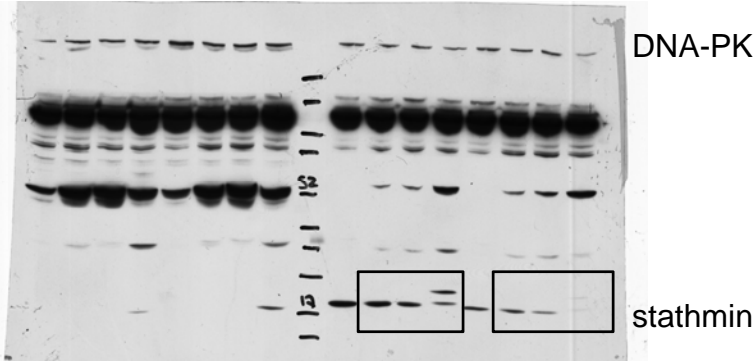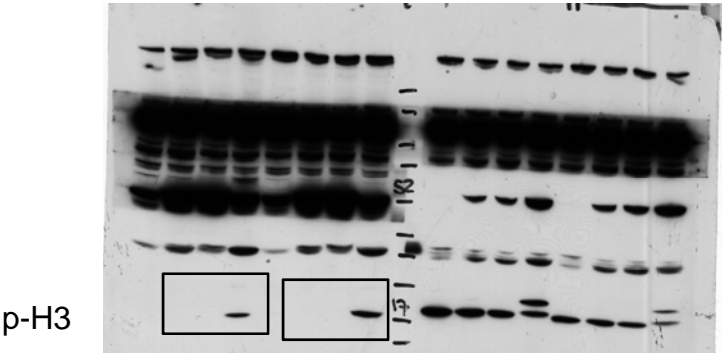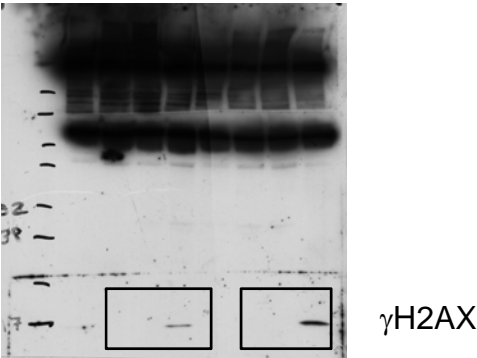

Supplement: Supplementary file 3 [file emmm0005-0707-sd3.pdf]

### Figure 3

A

**for MDAH and TOV112D**

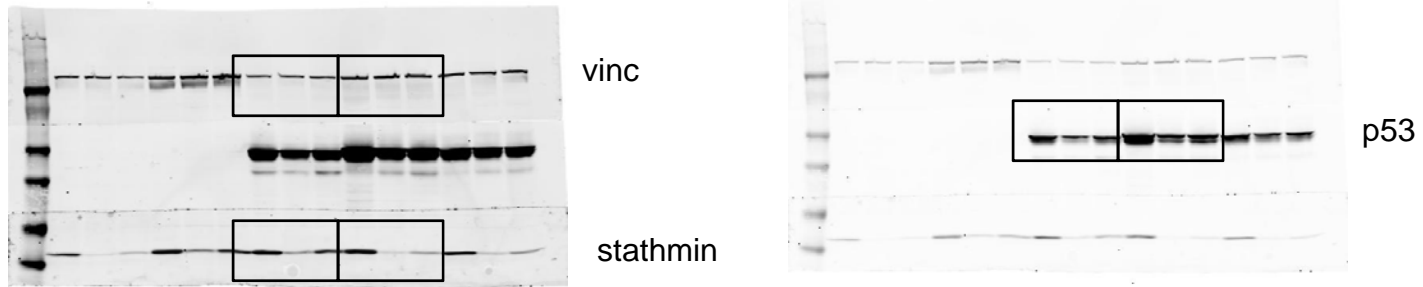

Figure 3

B

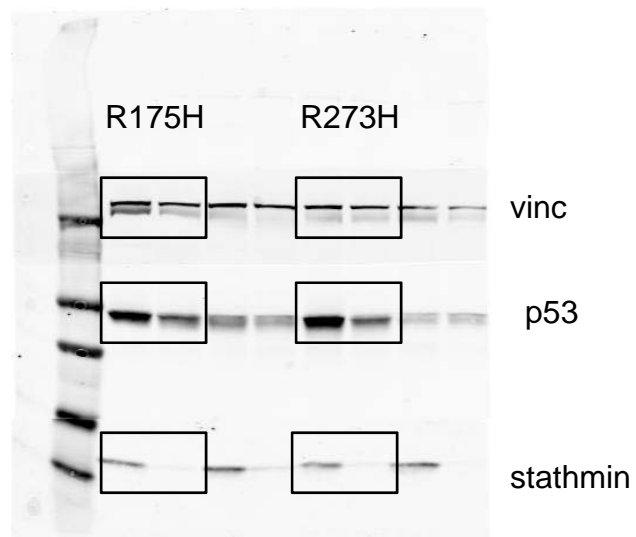

for TOV112D

C

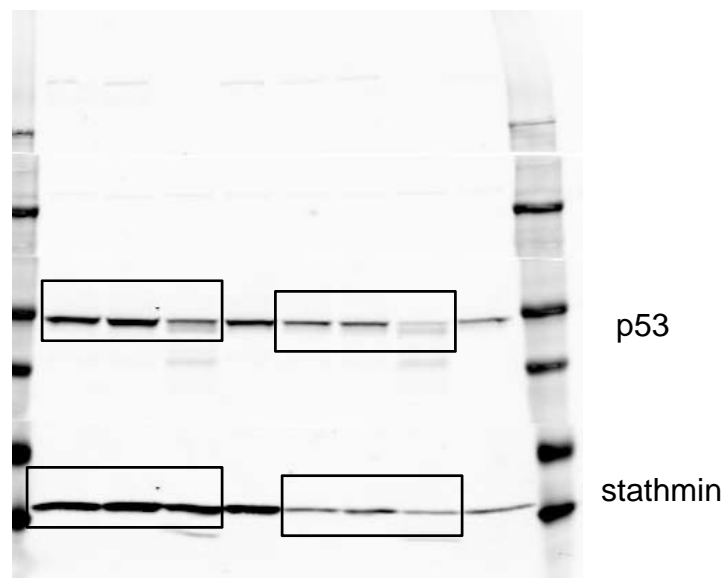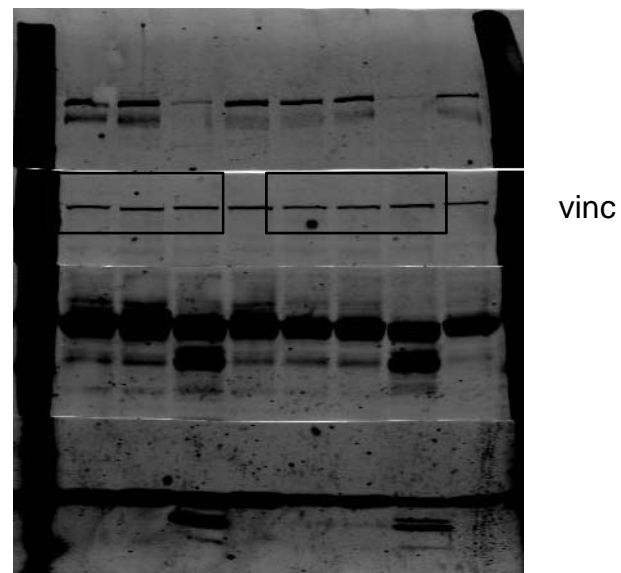

Figure 3

C

for MDAH

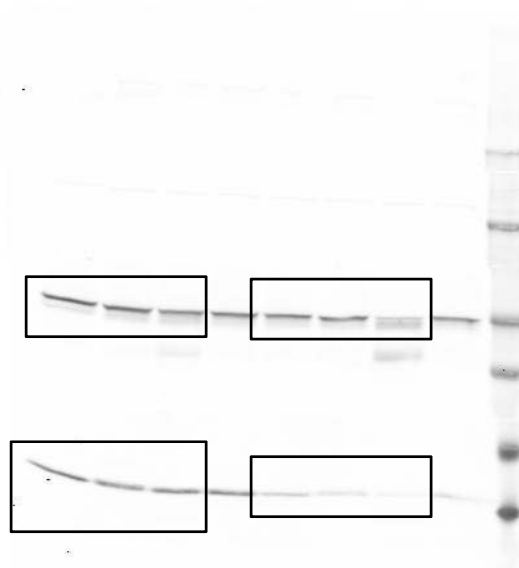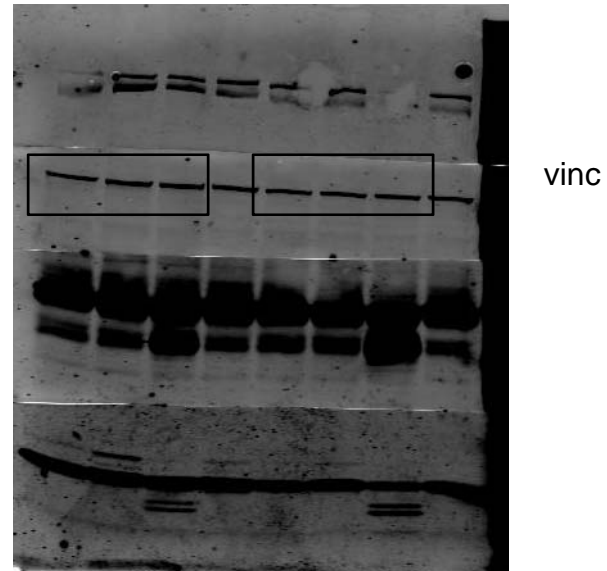

D

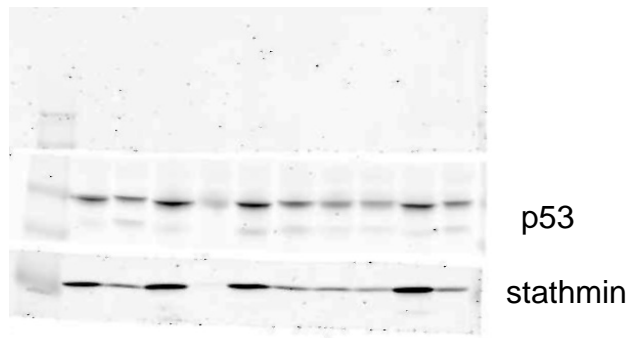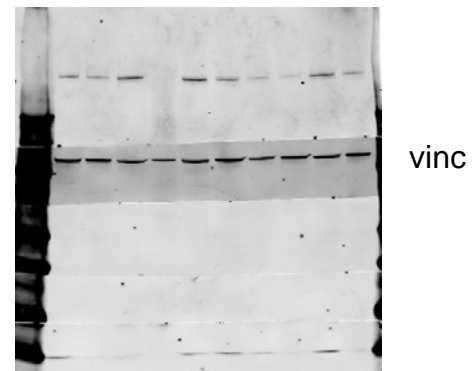

Figure 3

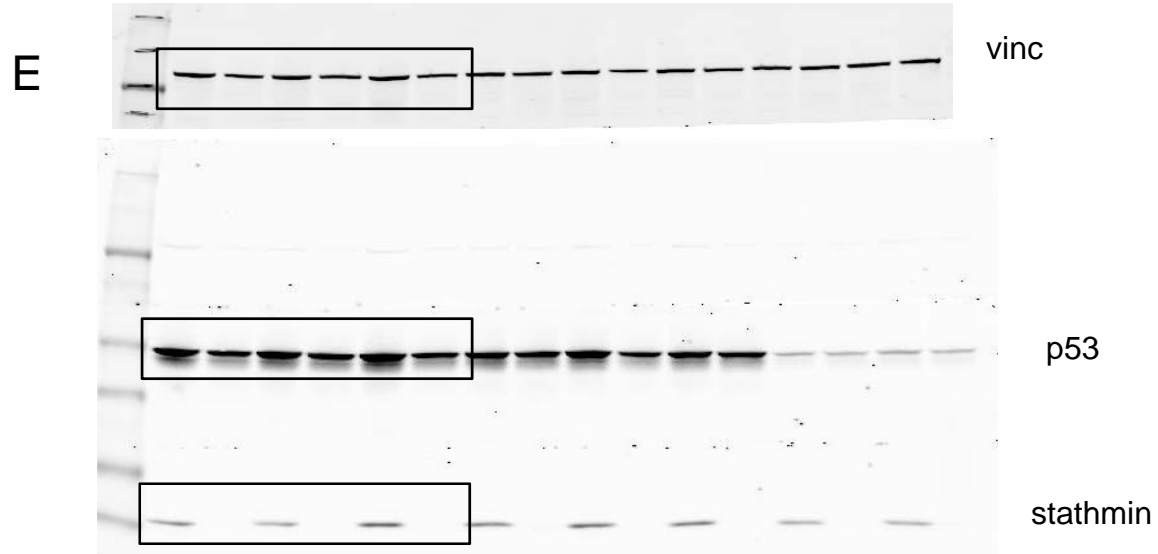

Figure 3

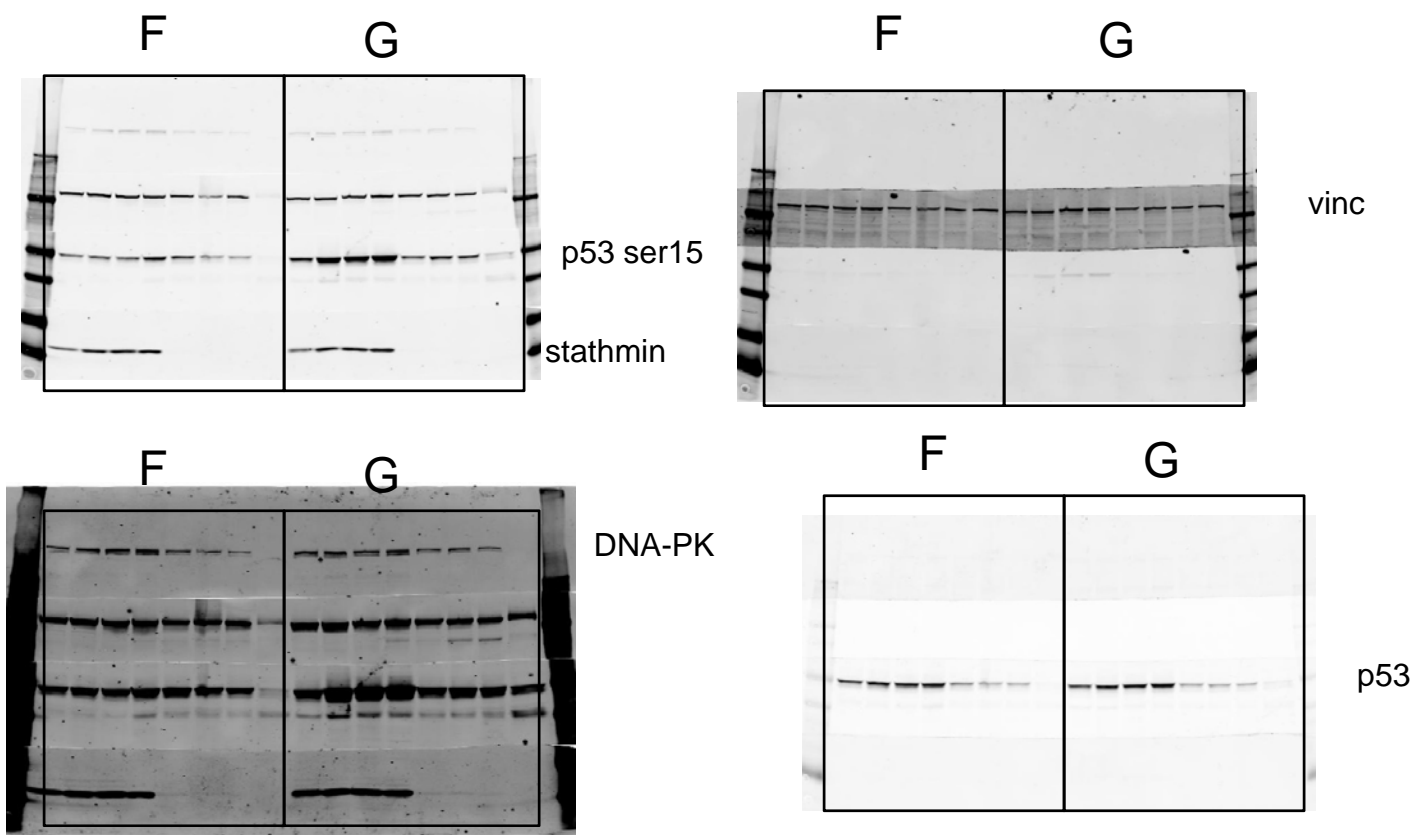

Supplement: Supplementary file 4 [file emmm0005-0707-sd4.pdf]

Figure 6

A

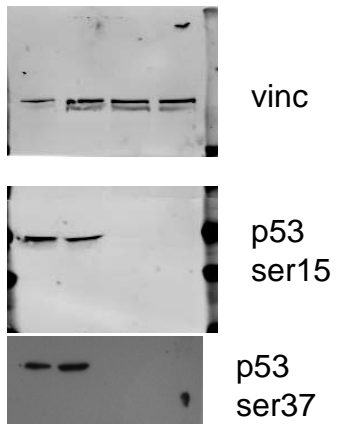

B

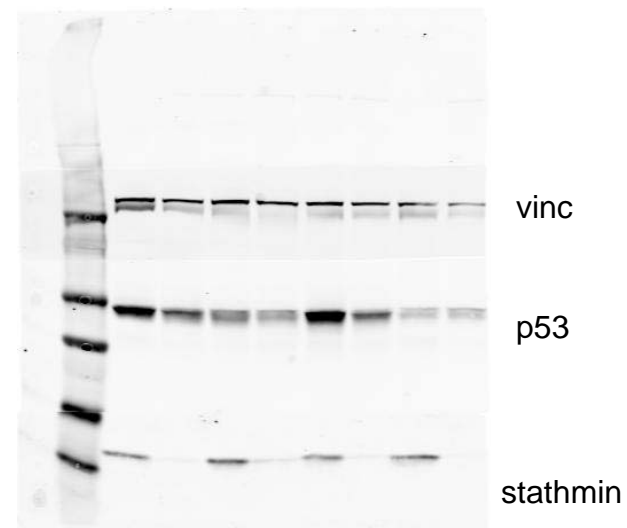

C

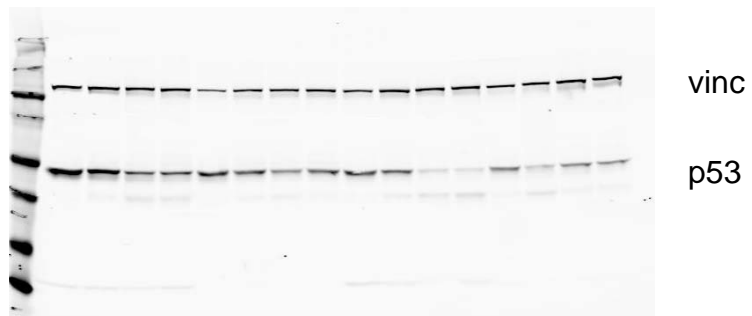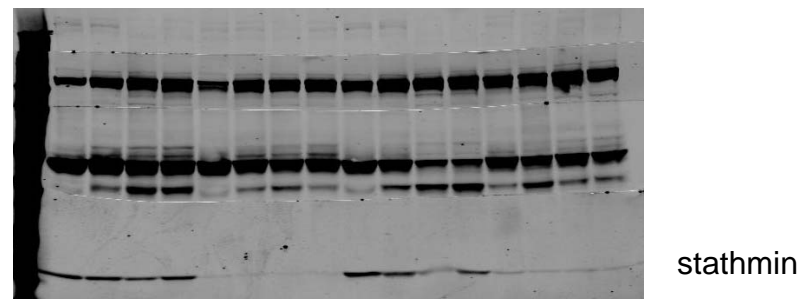

Figure 6

D

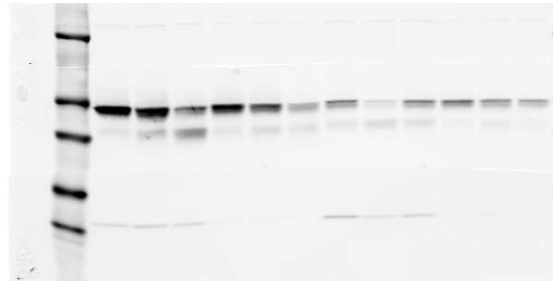

p53

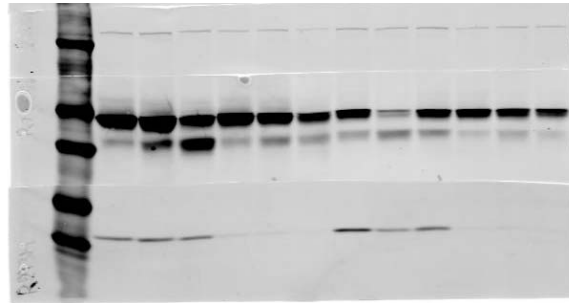

stathmin

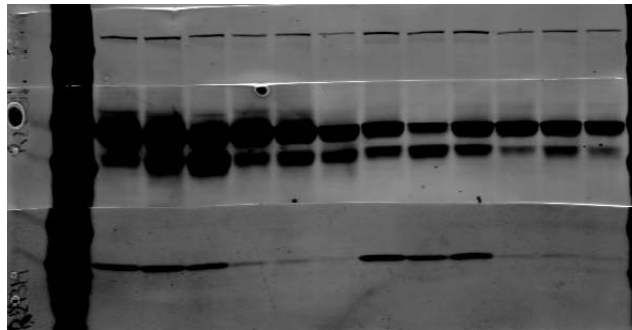

vinc

Supplement: Supplementary file 7 [file emmm0005-0707-sd7.pdf]

Figure 7

A

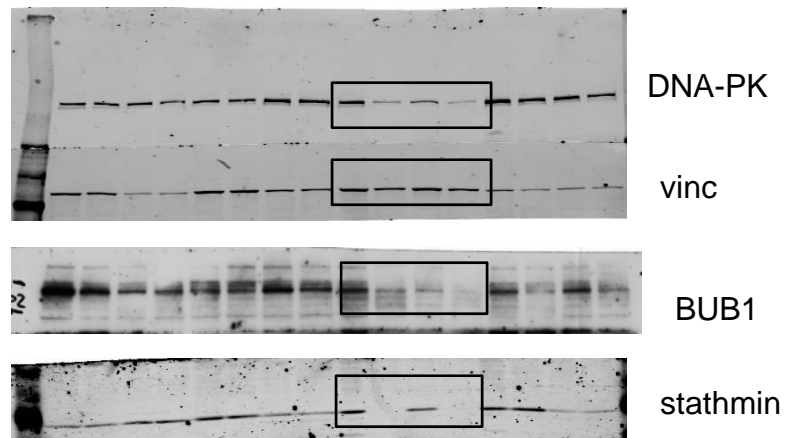

C

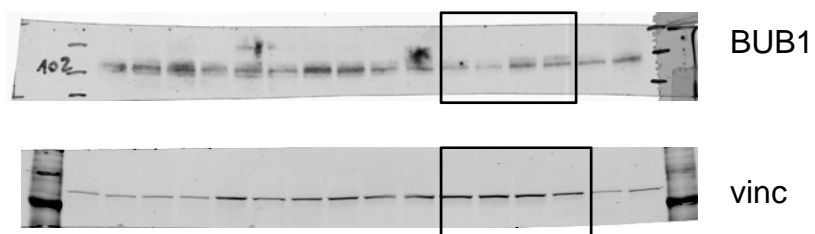

D

for MDAH

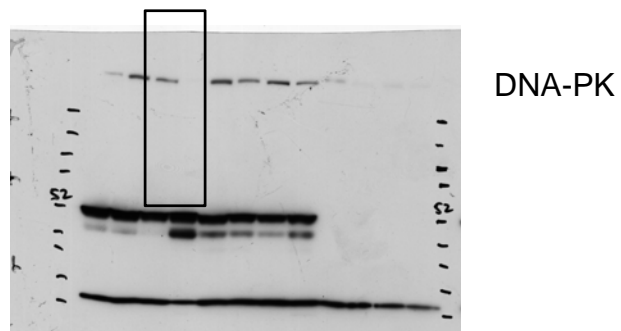

BUB1

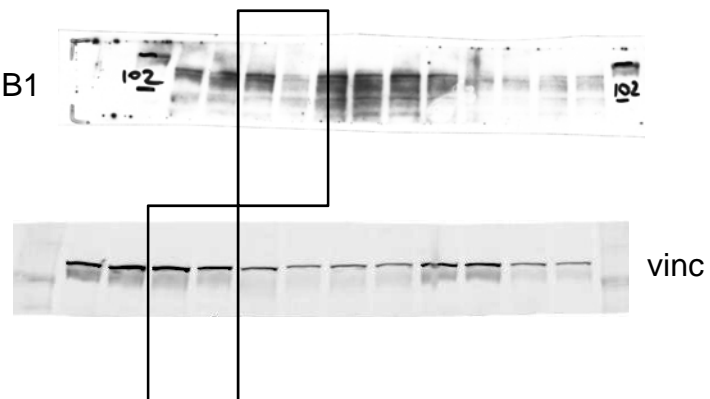

Figure 7

D

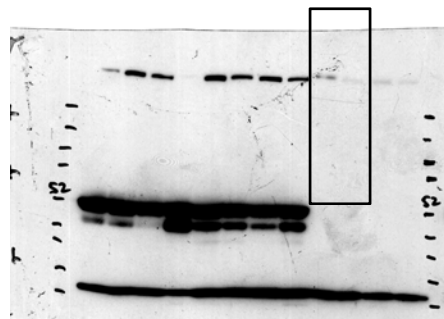

DNA-PK

for SKOV3

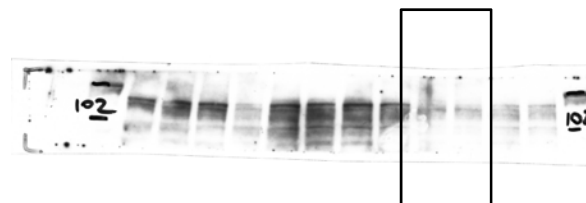

BUB1

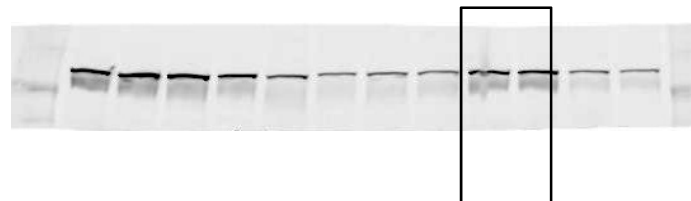

vinc

E

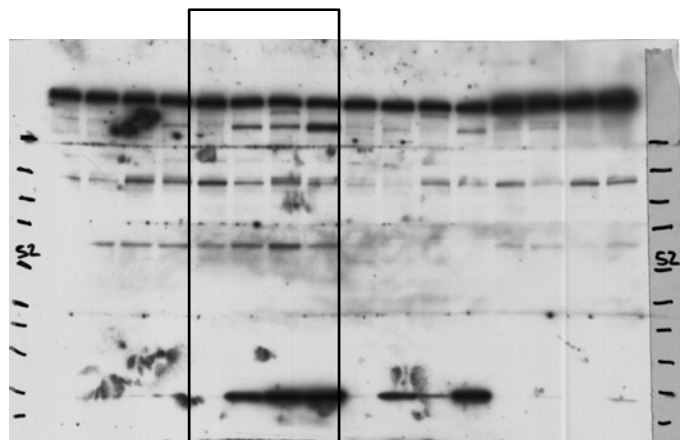

BUB1

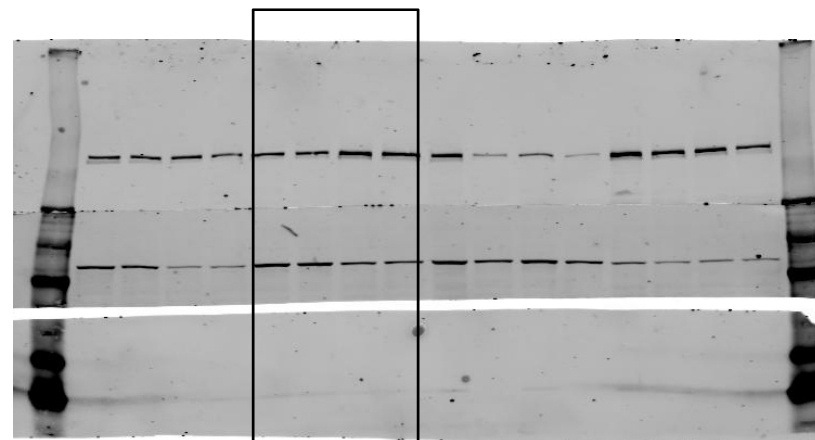

vinc

Supplement: Supplementary file 8 [file emmm0005-0707-sd8.pdf]

Figure 8

B

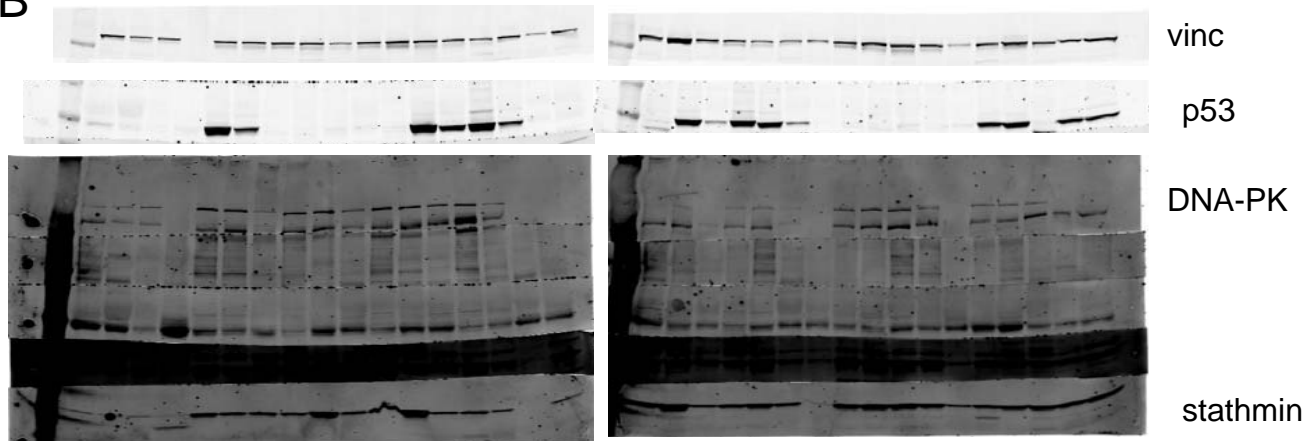

F

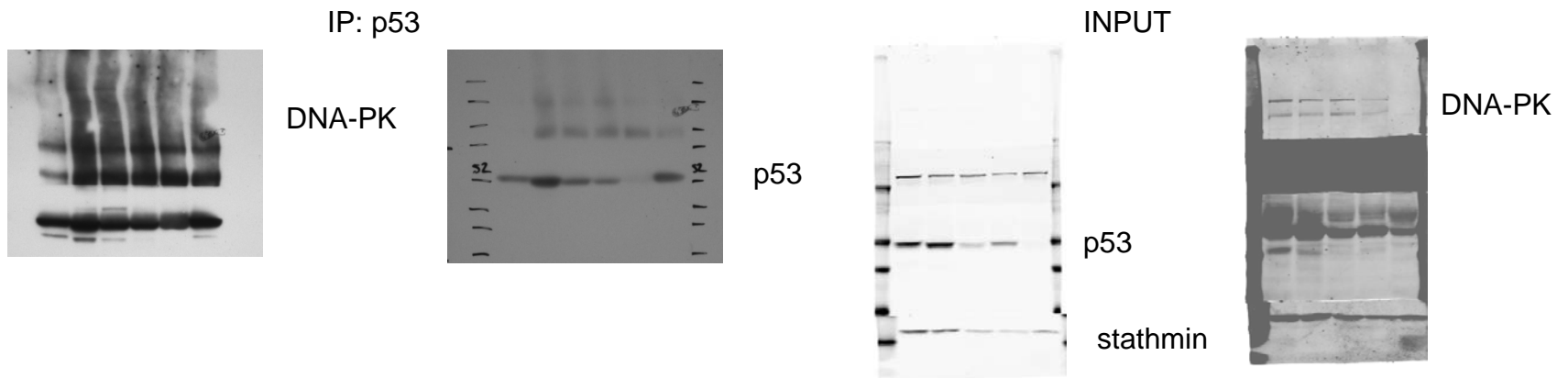

Supplement: Supplementary file 9 [file emmm0005-0707-sd9.pdf]
