# Supplementary material for: Stathmin regulates mutant p53 stability and transcriptional activity in ovarian cancer
Source: EMBO Mol Med. 2013 Apr 22;5(5):707–22. doi: 10.1002/emmm.201201504 (PMC3662314; doi:10.1002/emmm.201201504)

Figure 4

A

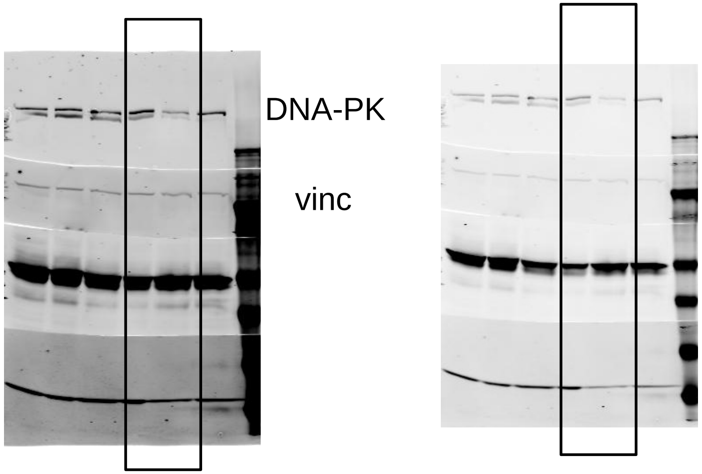

for MDAH

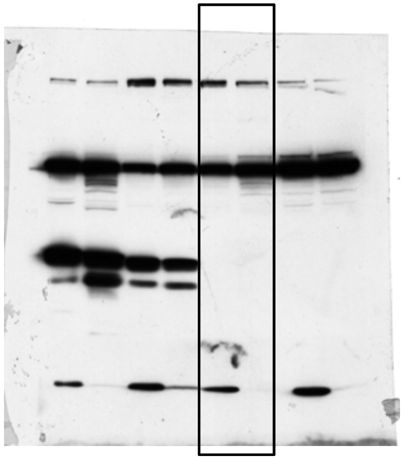

For OVCAR5

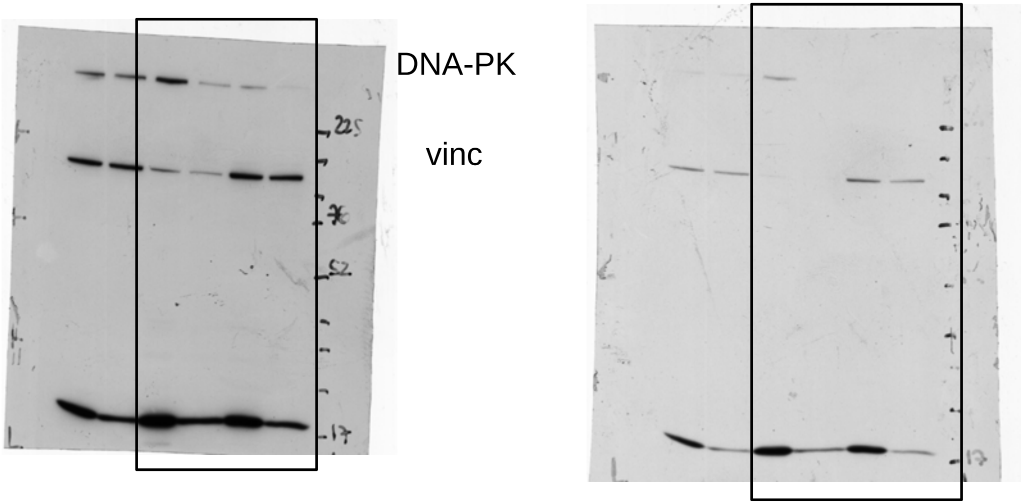

For TOV112D and SKOV3

Figure 4

B

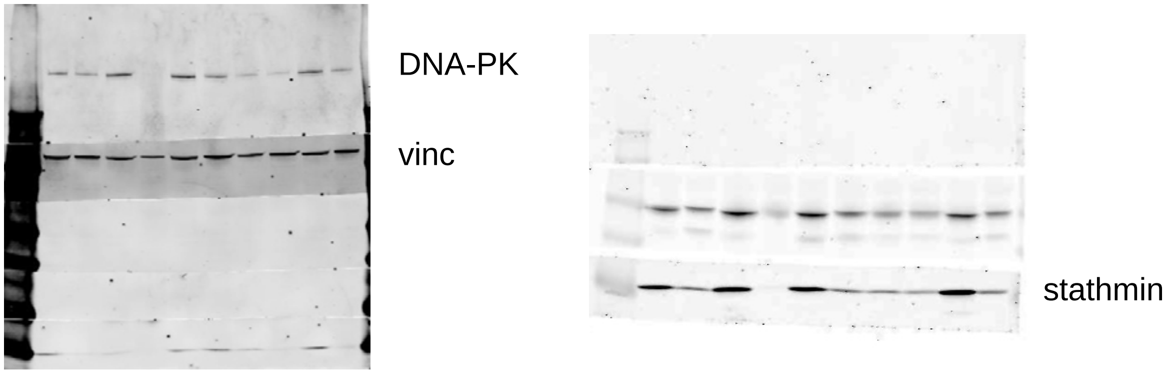

D

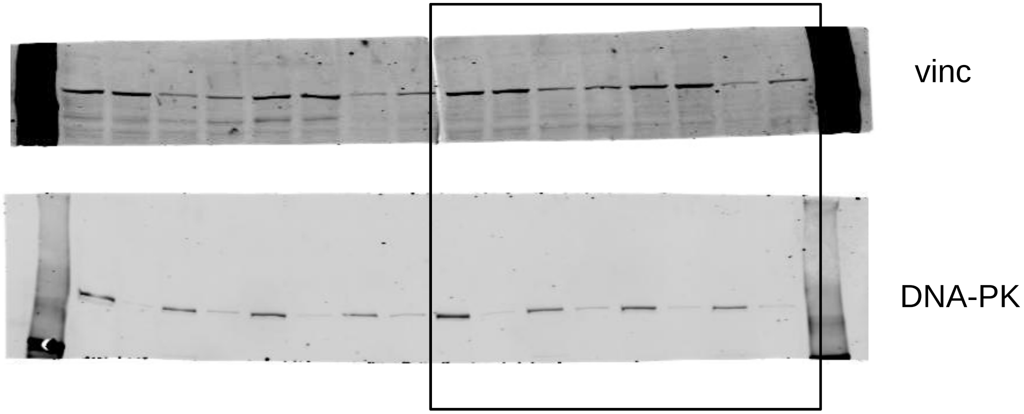

Supplement: Supplementary file 5 [file emmm0005-0707-sd5.pdf]
