# Supplementary material for: Stathmin regulates mutant p53 stability and transcriptional activity in ovarian cancer
Source: EMBO Mol Med. 2013 Apr 22;5(5):707–22. doi: 10.1002/emmm.201201504 (PMC3662314; doi:10.1002/emmm.201201504)
Supplement: Supplementary file 6 [file emmm0005-0707-sd6.pdf]

Figure 5

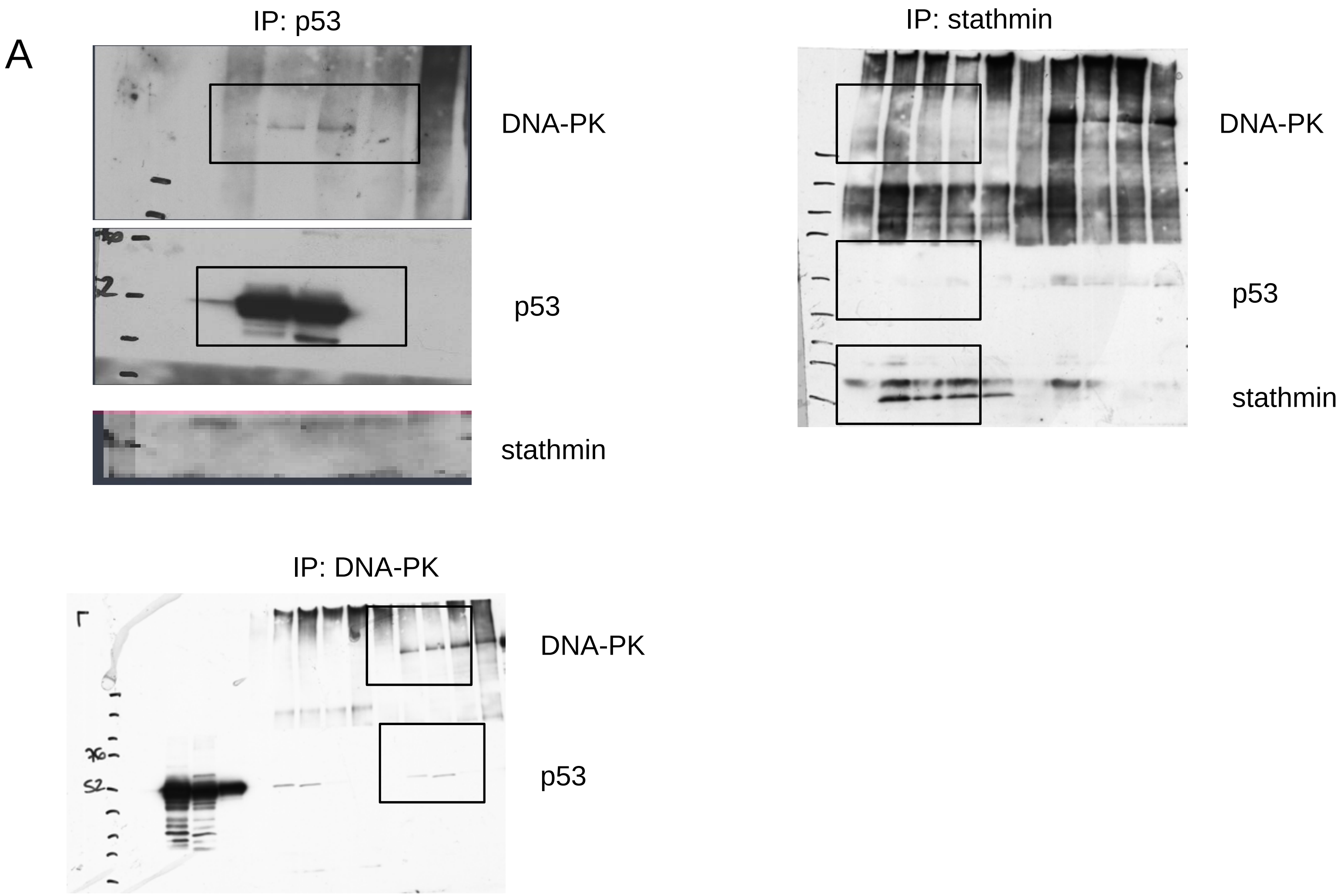
A
IP: p53
DNA-PK
p53
stathmin
IP: stathmin
DNA-PK
p53
stathmin
IP: DNA-PK
DNA-PK
p53

Figure 5

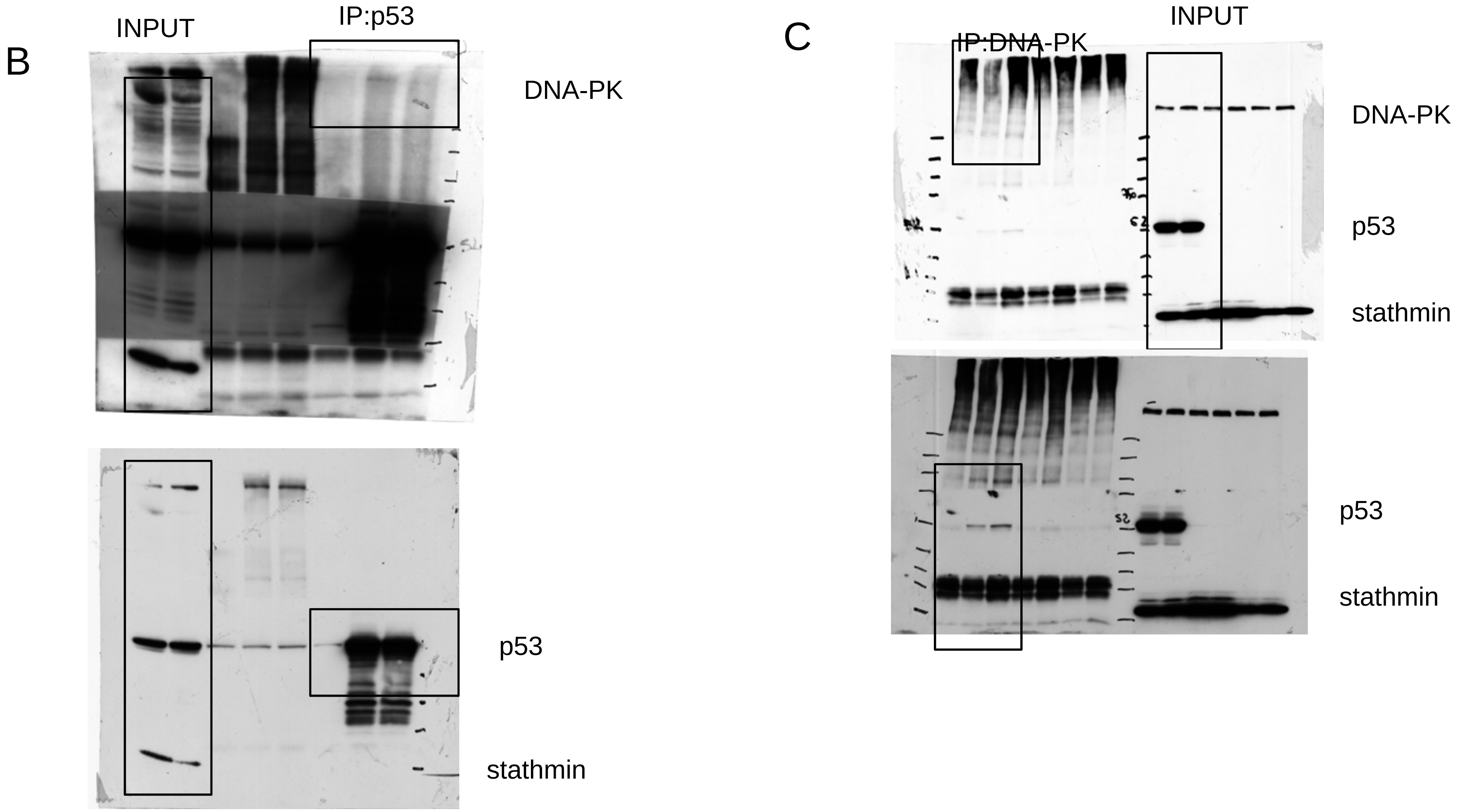
B
INPUT
IP:p53
DNA-PK
p53
stathmin
C
IP:DNA-PK
INPUT
DNA-PK
p53
stathmin
p53
stathmin

Figure 5

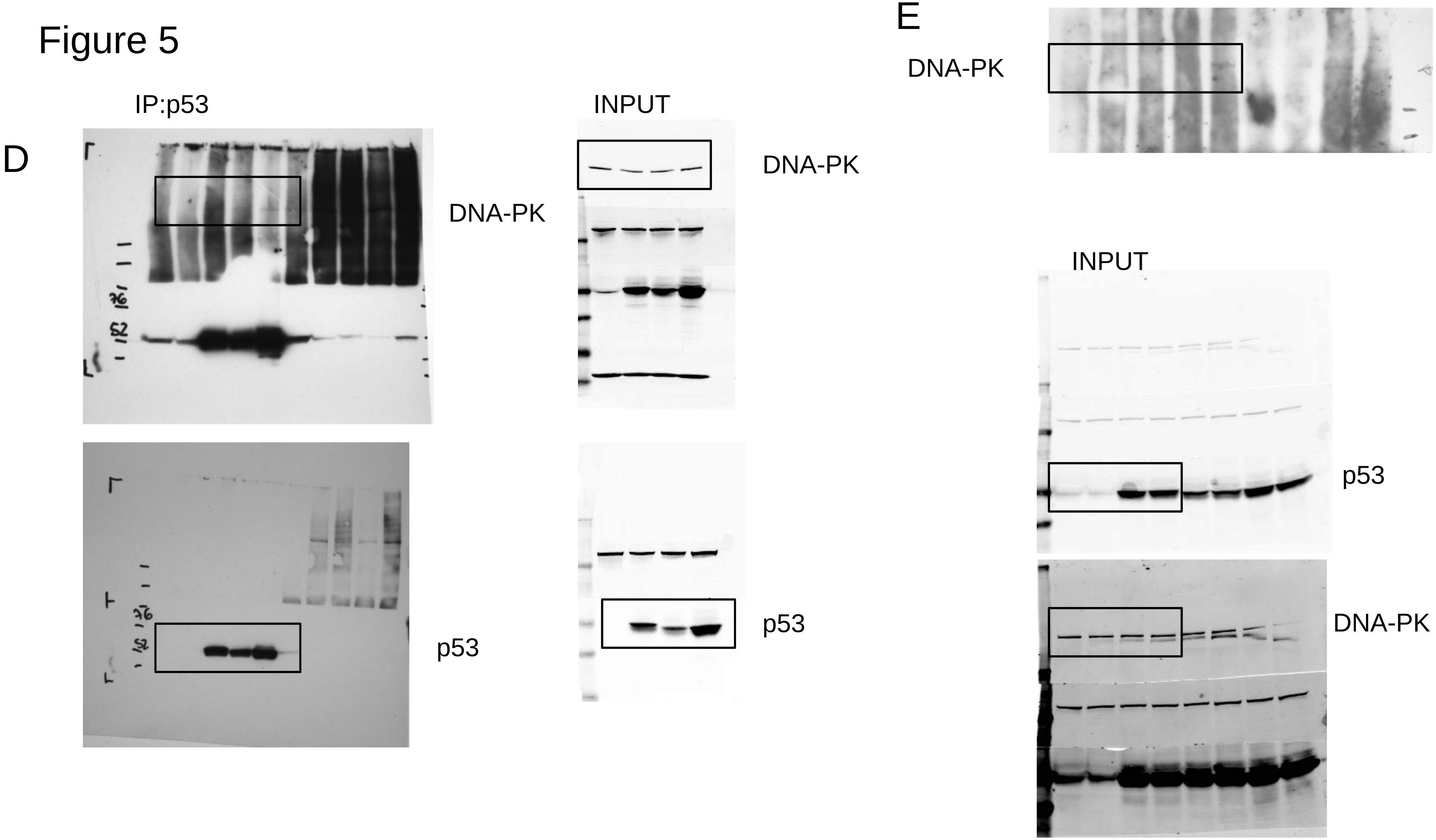

Figure 5

F

DNA-PK

p53 ser15

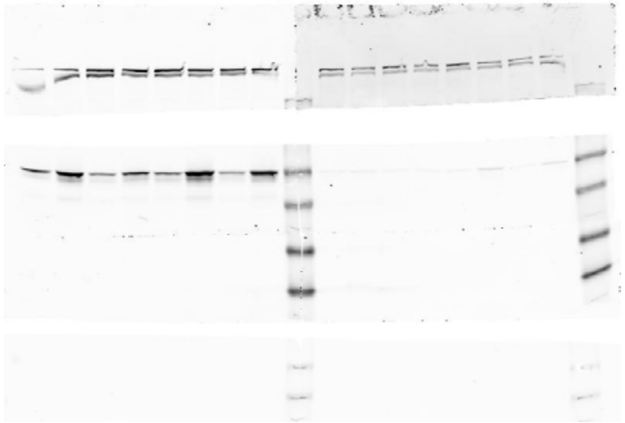

p-DNA-PK

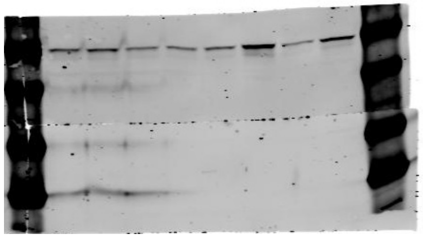

p53 ser37

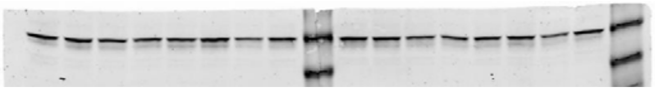

p53

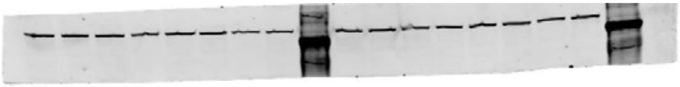

vinc

for MDAH

Figure 5

F

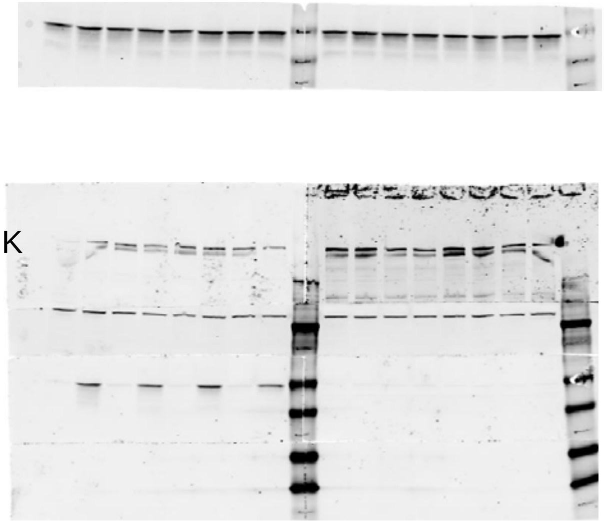

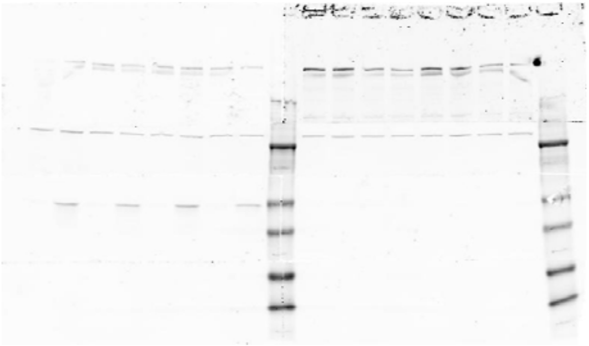

for TOV112D
