# Supplementary material for: Stathmin regulates mutant p53 stability and transcriptional activity in ovarian cancer
Source: EMBO Mol Med. 2013 Apr 22;5(5):707–22. doi: 10.1002/emmm.201201504 (PMC3662314; doi:10.1002/emmm.201201504)
Supplement: Supplementary file 10 [file emmm0005-0707-sd10.pdf]

Supporting Information

Stathmin regulates mutant p53 stability and transcriptional activity in ovarian cancer.

Maura Sonego, Monica Schiappacassi, Sara Lovisa, Alessandra Dall'Acqua, Marina Bagnoli, Francesca Lovat, Massimo Libra, Sara D'Andrea, Vincenzo Canzonieri, Loredana Militello, Marco Napoli, Giorgio Giorda, Barbara Pivetta, Delia Mezzanzanica, Mattia Barbareschi, Barbara Valeri, Silvana Canevari, Alfonso Colombatti, Barbara Belletti, Giannino Del Sal and Gustavo Baldassarre

<u>**Table of contents**</u>

Supporting data:

Table S1. p53 status in EOC cell lines and sensitivity to stathmin silencing. Related to Figure 1.

Figure S1. Effects of stathmin shRNAs and siRNA on EOC cells survival. Related to Figure 1.

Figure S2. Increased DNA damage in mitotic p53^{MUT}-expressing EOC cells. Related to Figure 2.

Figure S3. Stathmin-silencing alters the sensitivity to Taxol and CBDCA only in p53^{MUT} cells. Related to Figure 2.

Table S2. Results from Kinexus antibody array. Related to Figure 4.

Figure S4. Stathmin influences DNA-PKcs (but not ATM or ATR) expression in CBDCA treated cells. Related to Figure 4.

Figure S5. Stathmin silencing decreased BUB1 expression in a p53^{MUT}-dependent manner. Related to Figure 7.

Figure S6. The p53^{MUT}-depedent mitotic signature is overexpressed in Type II ovarian serous carcinomas. Related to Figure 8.

Figure S7. Co-expression between stathmin and the p53^{MUT}-depedent mitotic signature in Type II ovarian serous carcinomas. Related to Figure 8.

Table S3. Patient's clinical characteristics. Related to Figure 8.

Table S4. Association of stathmin expression with p53 status. Related to Figure 8.

Table S5. Association of stathmin expression with p53 overexpression. Related to Figure 8.

Table S6. Cox regression analysis of Case material A. Related to Figure 8.

Supporting Methods

Supporting References

Table S1: p53 status and sensitivity to stathmin silencing of EOC cell lines.

OVARIAN CELL LINES	HYSTOTYPE	p53 STATUS	MUTATION	SENSITIVITY TO sh-STM
TOV-21G	Clear cell	WILD TYPE		-
IGROV	Serous	HETERO	Y126C	-
**MDAH 2774**	**Endometrioid**	**MUT**	**R273H**	+
**TOV-112D**	**Endometrioid**	**MUT**	**R175H**	+
**SKOV3**	**Serous**	**NULL**		**-**
OVCAR8	Serous	MUT	del126-132	+
**OVCAR-5**	**Serous**	**NULL**		**-**
OV-90	Adenocarcinoma	MUT	S215R	N.D.
OVCAR 4	Serous	MUT	L130V	N.D.

All cell lines were authenticated by BMR Genomics srl Padova, Italia, on December 2011 according Cell ID ™ System (Promega) protocol and using Genemapper ID Ver 3.2.1 to identify DNA STR profiles.

Cell lines in which p53 mutation was confirmed by direct sequencing and used as a model of $p53^{MUT}$ or $p53^{null}$ HG-EOC are indicated in bold.

Sensitivity to stathmin silencing was evaluated by MTS assay and scored as follows; + = sensitive; - = not sensitive; N.D. = not determined.

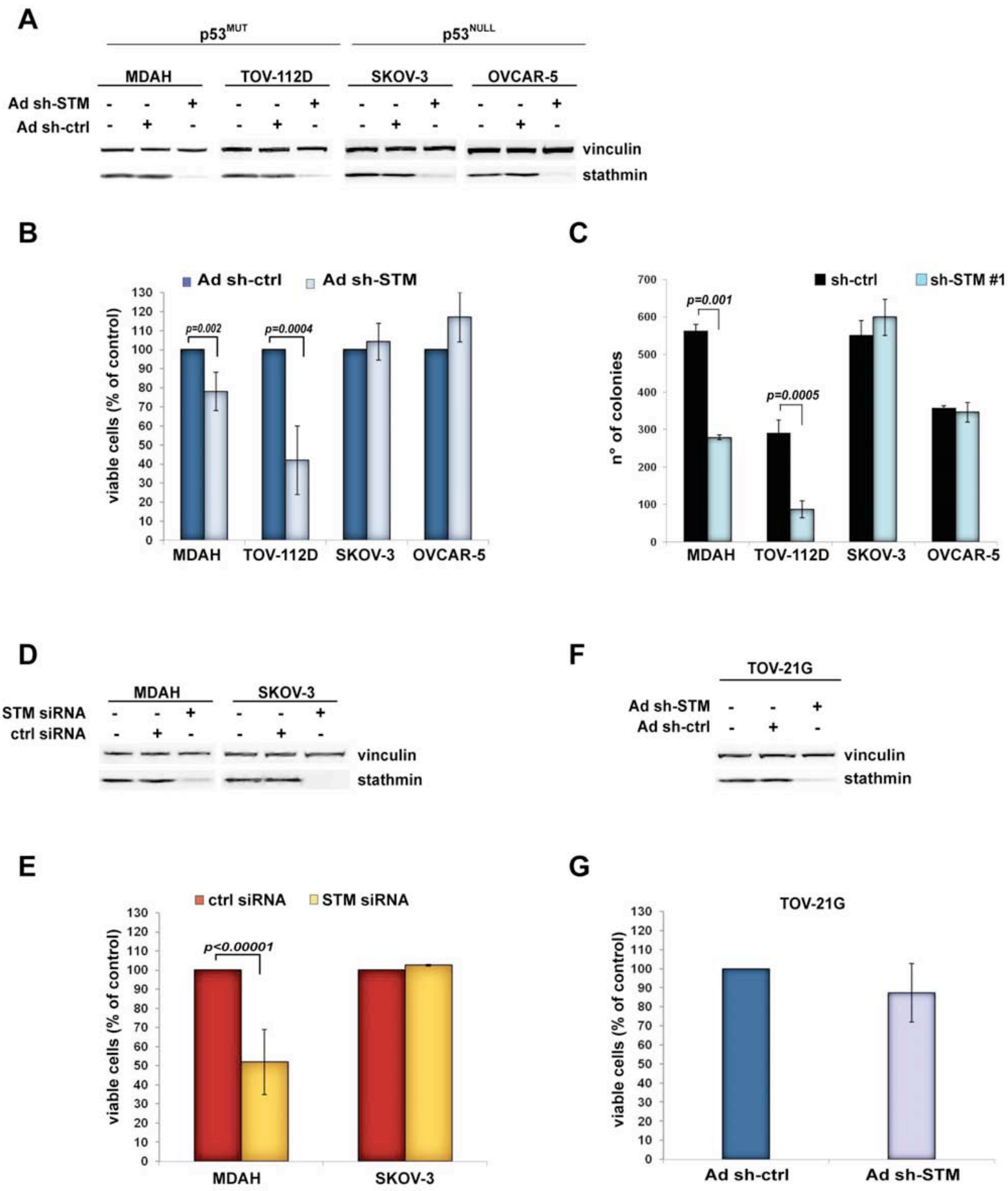
A
p53^MUT
p53^NULL
MDAH
TOV-112D
SKOV-3
OVCAR-5
Ad sh-STM
Ad sh-ctrl
vinculin
stathmin
B
Ad sh-ctrl
Ad sh-STM
viable cells (% of control)
p=0.002
p=0.0004
MDAH
TOV-112D
SKOV-3
OVCAR-5
C
sh-ctrl
sh-STM #1
n° of colonies
p=0.001
p=0.0005
MDAH
TOV-112D
SKOV-3
OVCAR-5
D
MDAH
SKOV-3
STM siRNA
ctrl siRNA
vinculin
stathmin
F
TOV-21G
Ad sh-STM
Ad sh-ctrl
vinculin
stathmin
E
ctrl siRNA
STM siRNA
viable cells (% of control)
p<0.00001
MDAH
SKOV-3
G
TOV-21G
viable cells (% of control)
Ad sh-ctrl
Ad sh-STM

Figure S1. Effects of stathmin silencing on HG-EOC cell survival.

(**A**) and (**D**) Western blot analyses evaluating stathmin expression in HG-EOC cell lines after transduction with control shRNA (Ad sh-ctrl) or stathmin shRNAs (Ad sh-STM) (**A**) or with siRNA oligos (ctrl siRNA or STM siRNA) (**D**). Vinculin was used as loading control.

(**B**) and (**E**) MTS assay comparing cell viability of the indicated HG-EOC cell lines transduced with Ad shRNAs (**B**) or transfected with siRNAs (**E**). Results are expressed as percentage of viable stathmin-silenced respect to control cells and represent the mean (± SD) of three independent experiments performed in quadruplicate.

(**C**) Colony assay of HG-EOC cells silenced or not for stathmin, as indicated. Quantitative data were generated from a minimum of 3 replicates and represent the mean (± SD) of three independent experiments.

(**F**) Expression of stathmin in TOV-21G cell line transduced with adenoviral control shRNA (Ad sh-ctrl) or stathmin shRNAs (Ad sh-STM). Vinculin was used as loading control.

(**G**) MTS assay comparing cell viability of TOV-21G after transduction with control shRNA (Ad sh-ctrl) or stathmin shRNAs (Ad sh-STM). Results are expressed as percentage of viable stathmin-silenced respect to control cells and represent the mean (± SD) of three independent experiments performed in quadruplicate.

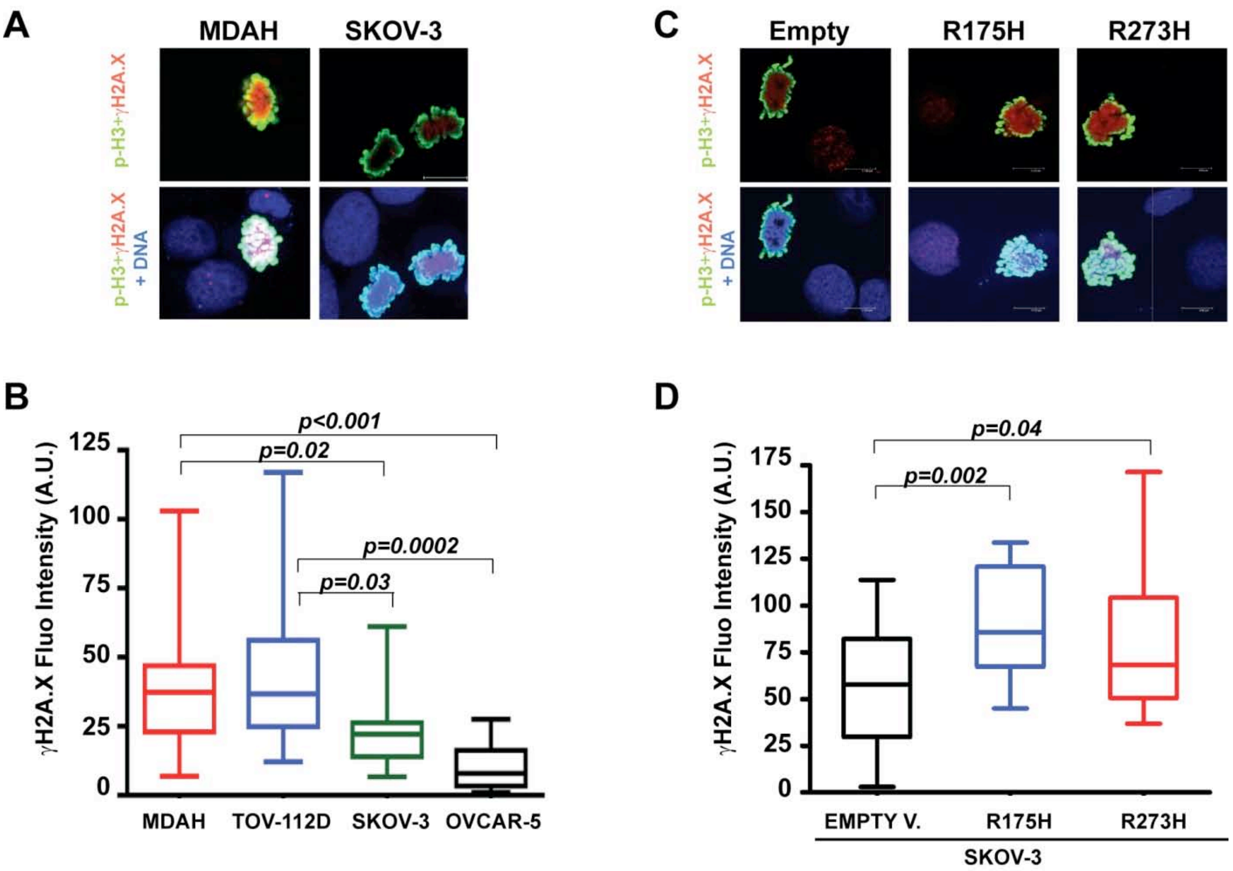

Figure S2. p53^{MUT}-expressing HG-EOC cells display increased DNA damage during mitotis.

(**A**) Immunofluorescence analyses evaluating the expression of γH2A.X (in red) marker of DNA damage, and pH3 (in green) marker of mitosis, in the indicated cells. DNA staining (propidium iodide) is pseudocolored in blue. A representative fluorescence image of each analyzed cell lines is reported. (**B**) Quantification of γH2A.X fluorescence intensity in 20 mitotic cells for each of the indicated HG-EOC cell lines, using the SP2 Leica software.

(**C**) γH2A.X (in red) and pH3 (in green) and DNA staining (in blue) in p53^{null} cells (SKOV-3) overexpressing the mutant forms of p53. A representative fluorescence image of each analyzed cell lines is reported. (**D**) Same as in (B), but regarding cells described in (C).

A

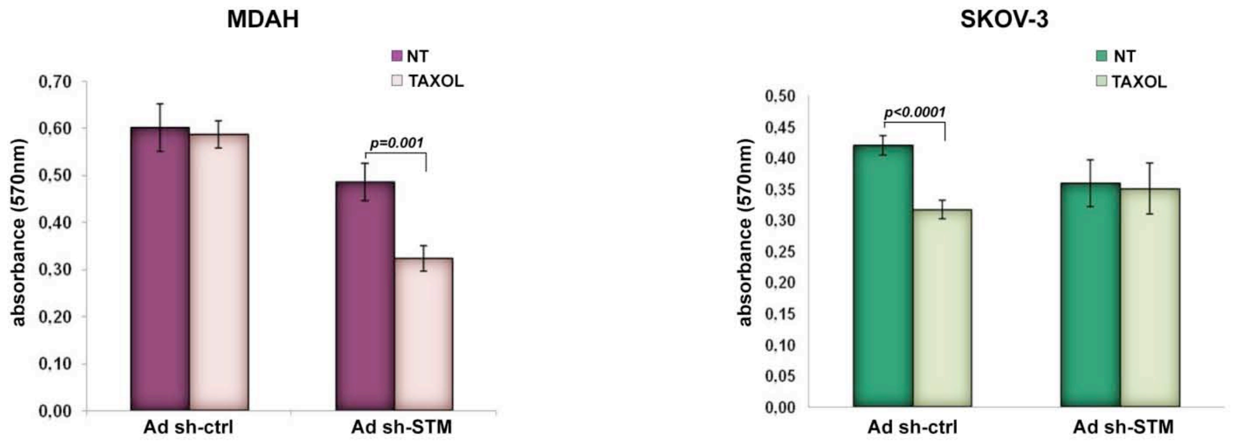

B

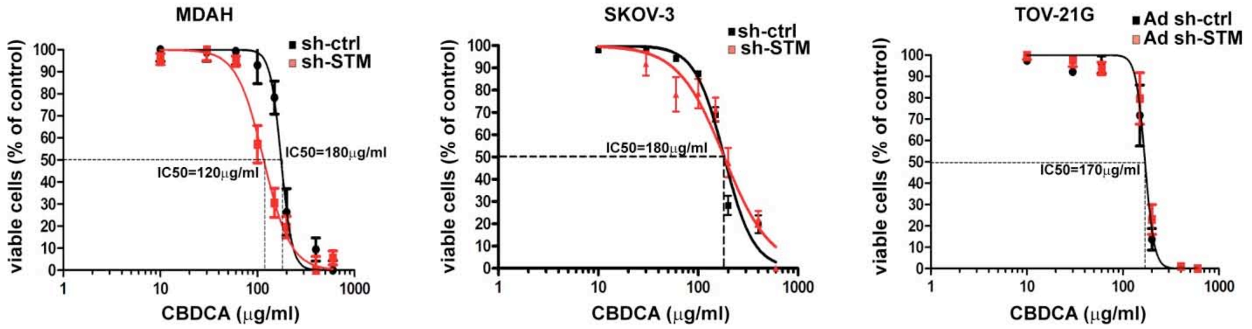

C

IP injected cells	Positive tumor growth *	N° of tumor mass/animal (range)
TOV-112D sh-ctrl	4/4	8 (6 - 10)
TOV-112D sh-STM	2/4	1

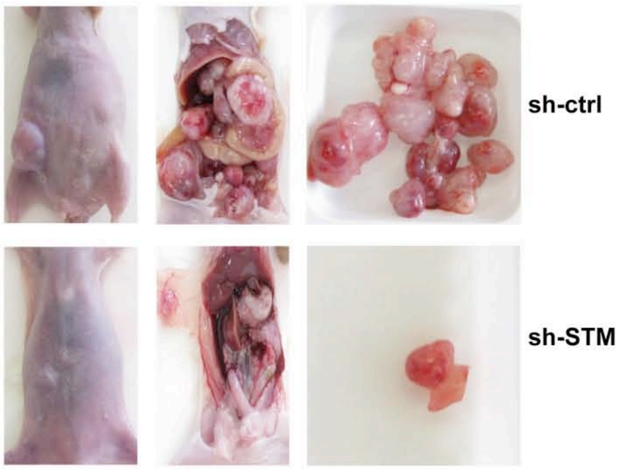

Figure S3. Stathmin-silencing alters the sensitivity to Taxol and CBDCA only in p53^{MUT} HG-EOC cells.

(**A**) MTS assay in MDAH and SKOV-3 stably transduced with lentiviral shRNAs and treated with Taxol (10 nM for MDAH and 100 nM for SKOV3), as indicated. Results are the mean (± SD) of three independent experiments performed in quadruplicate.

(**B**) Evaluation of CBDCA IC50 in MDAH, SKOV-3 and TOV-21G cells treated with increasing doses of CDBCA in the presence or not of stathmin shRNA. Curves were fitted by non-linear regression using GraphPad Prism. Results are expressed as percentage of viable cells and are the mean (± SD) of three independent experiments performed in quadruplicate.

(**C**) Representative images of nude mice intraperitoneally injected with TOV-112D cells stably transduced with sh-ctrl or sh-STM (right panels). Results of this experiment are summarized in the table on the left.

Table S2. **Results from Kinexus antibody array.**

The array was used to detect changes in cellular signaling pathways after treatment with CBDCA in MDAH cells transduced with Ad sh-STM or Ad sh-ctrl. The results are reported as fold change over control. Purple boxes, in Z-ratio column, indicate upregulated target genes, while blue boxes downregulated genes. The Z-Ratio is the result of a Z Test of statistical significance and was directly performed by the company in a blinded fashion.

Target Protein Name	Phospho Site (Human)	Full Target Protein Name	Z-ratio (2, 1)
PKCg	T655	Protein-serine kinase C gamma	2,83
Hsp27	S15	Heat shock 27 kDa protein beta 1 (HspB1)	2,64
IRS1	Y1179	Insulin receptor substrate 1	2,62
NME7	Pan-specific	Nucleotide diphosphate kinase 7 (nm23-H7)	2,42
Mcl1	Pan-specific	Myeloid cell leukemia differentiation protein 1	2,04
MST1	Pan-specific	Mammalian STE20-like protein-serine kinase 1 (KRS2)	2,03
MEF-2	Pan-specific	Myelin expression factor 2 (MYEF2)	1,86
PP6C	Pan-specific	Protein-serine phosphatase 6 - catalytic subunit (PPVC)	1,85
Erk3	Pan-specific	Extracellular regulated protein-serine kinase 3	1,82
PKM2	Pan-specific	Pyruvate kinase, isozymes M1/M2	1,77
CDK2	Pan-specific	Cyclin-dependent protein-serine kinase 2	1,71
IRS1	S312	Insulin receptor substrate 1	1,70
Raf1	Pan-specific	Raf1 proto-oncogene-encoded protein-serine kinase	1,70
PRK1 (PKN1)	Pan-specific	Protein kinase C-related protein-serine kinase 1	1,65
PP1/Cb	Pan-specific	Protein-serine phosphatase 1 - catalytic subunit - beta isoform	1,62
STK33	Pan-specific	FLJ35932 protein-serine kinase	1,56
Hsp90a	Pan-specific	Heat shock 90 kDa protein alpha	1,55
PRP4K	Pan-specific	Protein-serine kinase PRP4 homolog	1,55
Paxillin 1	Y118	Paxillin 1	1,51
PP4C	Pan-specific	Protein-serine phosphatase X - catalytic subunit (PPX/C)	1,49
GSK3ab	Pan-specific	Glycogen synthase-serine kinase 3 alpha/beta	-1,41
FGFR2	Pan-specific	Fibroblast growth factor receptor-tyrosine kinase 2 (BEK)	-1,41
PKA R2a	S98	cAMP-dependent protein-serine kinase regulatory type 2 subunit alpha	-1,43
JAK1	Pan-specific	Janus protein-tyrosine kinase 1	-1,48
Histone H1	phospho CDK1 sites	Histone H1 phosphorylated	-1,50
DAPK1	Pan-specific	Death-associated protein kinase 1	-1,55
DNAPK	Pan-specific	DNA-dependent protein kinase catalytic subunit	-1,57
GCK	Pan-specific	Germinal centre protein-serine kinase	-1,61
EGFR	Y1197	Epidermal growth factor receptor-tyrosine kinase	-1,62
GNB2L1	Pan-specific	Guanine nucleotide-binding protein beta (receptor for activated C kinase 1 (RACK1))	-1,69
Histone H3	T11	Histone H3.3	-1,69
Erk1/2	T202+Y204; T184/Y186	Extracellular regulated protein-serine kinase 1 (p44 MAP kinase), 2 (p42MAP kinase)	-1,73
DFF35, DFF45	Pan-specific	DNA fragmentation factor alpha (ICAD) 35-kDa, 45-kDa subunit	-1,78
Erk5	Pan-specific	Extracellular regulated protein-serine kinase 5 (Big MAP kinase 1 (BMK1))	-1,84
CASP7	Pan-specific	Pro-caspase 7 (ICE-like apoptotic protease 3 (ICE-LAP3), Mch3)	-1,87
GFAP	S8	Glial fibrillary acidic protein	-1,94
BLK	Pan-specific	B lymphoid tyrosine kinase	-2,02
Erk1/2	Pan-specific	Extracellular regulated protein-serine kinase 1 (p44 MAP kinase), 2 (p42MAP kinase)	-2,13
Histone H2A.X	S139	Histone H2A variant X	-2,15
EphA1	Pan-specific	Ephrin type-A receptor 1 protein-tyrosine kinase	-2,59
FLT4	Pan-specific	Vascular endothelial growth factor receptor-protein-tyrosine kinase 3 (VEGFR3)	-2,61
Erk1/2	Pan-specific	Extracellular regulated protein-serine kinase 1 (p44 MAP kinase), 2 (p42MAP kinase)	-3,11
Bax	Pan-specific	Apoptosis regulator Bcl2-associated X protein	-3,37
ErbB2 (HER2)	Pan-specific	ErbB2 (Neu) receptor-tyrosine kinase	-3,46
Erk1/2	T202+Y204; T184/Y186	Extracellular regulated protein-serine kinase 1 (p44 MAP kinase), 2 (p42MAP kinase)	-3,61
IkBb	Pan-specific	Inhibitor of NF-kappa-B beta (thyroid receptor interacting protein 9)	-3,62

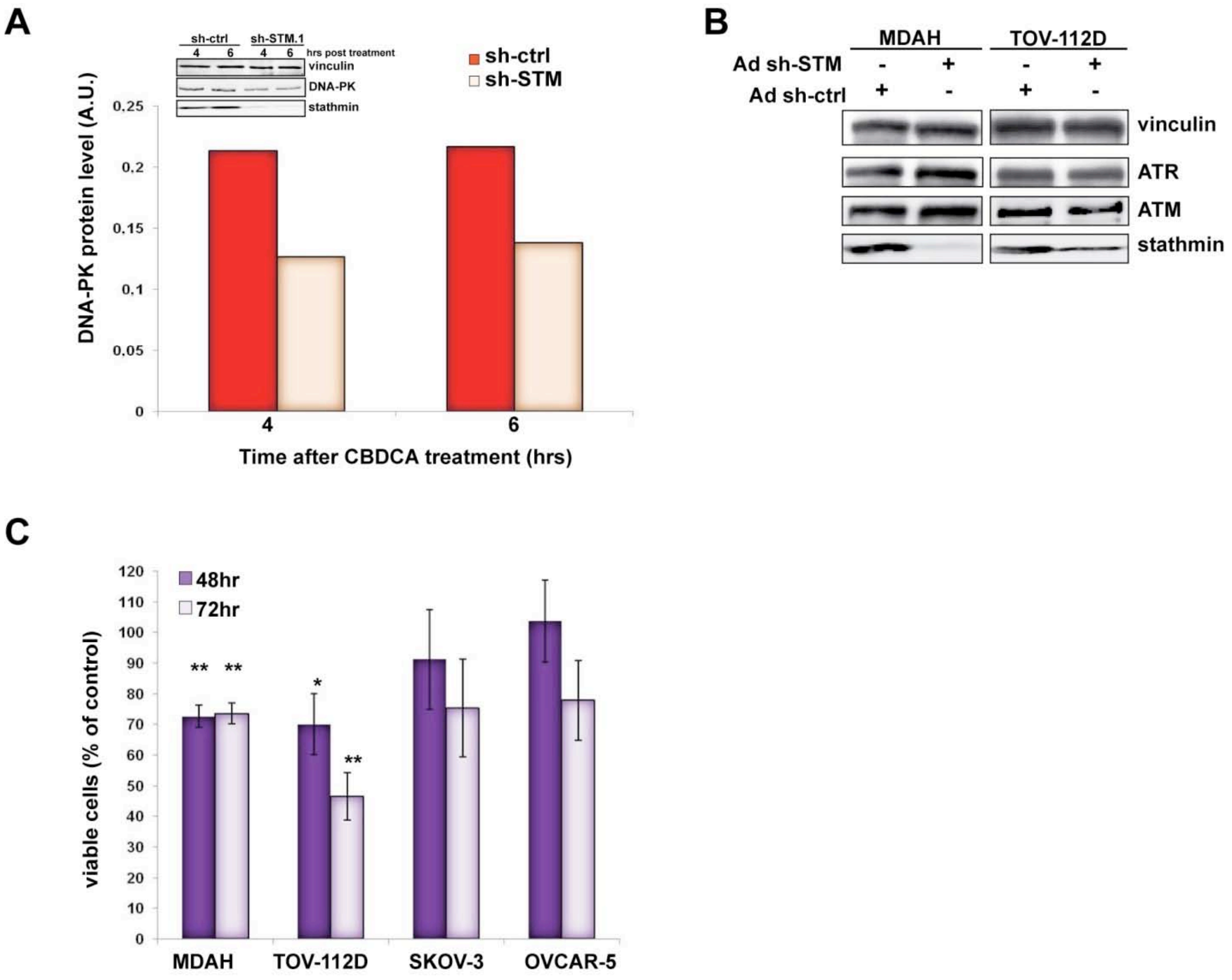

Figure S4. Stathmin influences DNA-PKcs (but not ATM or ATR) expression in CBDCA treated cells.

(**A**) Densitometric quantification of DNA-PK protein levels (normalized respect to vinculin) in MDAH cells stably expressing sh-STM or sh-ctrl, treated with CBDCA and analyzed 4 and 6 hours after treatment, as indicated. Data represent the mean of two independent experiments. A typical western blot is reported in the inset.

(**B**) ATM and ATR expression in MDAH and TOV-112D cells after transduction with control (Ad sh-ctrl) or stathmin shRNAs (Ad sh-STM). Vinculin was used as loading control.

(**C**) EOC cell lines viability after 48 or 72 hours of treatment with the specific DNA-PK inhibitor NU7441 (10 μM). Results are expressed as percentage of viable treated cells respect to untreated cells and represent the mean (± SD) of three independent experiments performed in quadruplicate. *$p< 0.05$ **$p\leq 0.001$

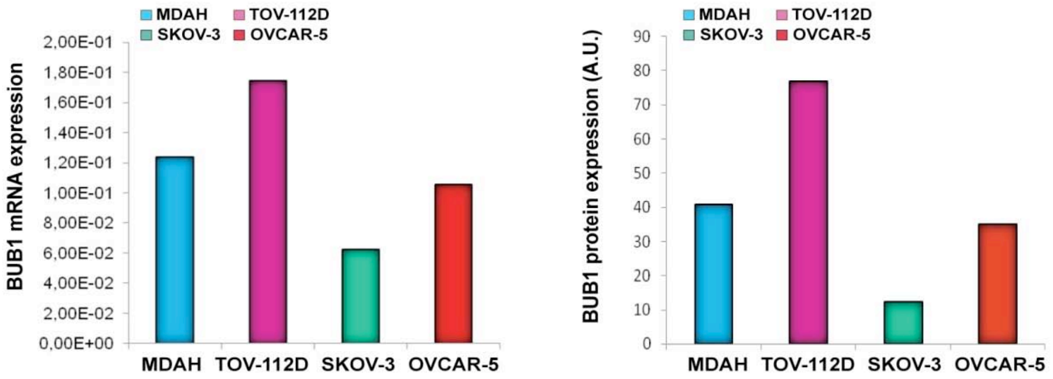

Figure S5. BUB1 protein expression in HG-EOC cells parallels its mRNA levles.

In the left graph BUB1 mRNA expression in the indicated HG-EOC cell lines, as determined by quantitative RT-PCR. BUB1 mRNA levels was analyzed in duplicate, normalized against GAPDH and SDHA housekeeping genes expression.

In the right graph Densitometric quantification of BUB1 protein levels (normalized respect to vinculin) as evaluated from western blot analysis, in the indicated cell lines.

Table S3. Patients' clinical characteristics.

	Case Material A n =128		Case Material B n=72		Case Material C n=51	
Characteristics	N°	%	N°	%	N°	%
Age, years						
	(mean, median 58; range 23-84)		(mean 55, median 56; range 25-75)		(mean 61, median 62; range 39-83)	
≤55			32	44	17	33
>55			40	56	34	77
Tumor histotype						
Serous	84	66	52	72	51	100
Undifferentiated	12	9	9	13	-	-
Clear cell	12	9	-	-	-	-
Endometrioid	12	9	11	15	-	-
Mucinous	6	5	-	-	-	-
Others + Mixed	2	2	-	-	-	-
Not available	-	-	-	-	-	-
Tumor stage (FIGO)						
I	24	18	-	-	-	-
II	15	12	-	-	3	6
III	64	50	57	79	17	33
IV	23	18	15	21	29	57
Not available	2	2	-	-	2	4
Tumor grade						
1, well differentiated	9	7	-	-	3	6
2, moderately differentiated	49	38	16	22	4	8
3, poorly differentiated	53	42	46	64	43	84
Undifferentiated	12	9	9	13		
Not available	5	4	1	1	1	2
Amount of residual disease						
NED	47	37	5	7		
<1cm, mRD	19	15	13	18		
>1cm, GRD	46	36	54	75		
Not available	16	12	-	-	51	100
Frontline treatment						
None	7	6	-	-		
Platinum without taxanes	81	63	5	7		
Platinum/paclitaxel	35	27	13	18		
Platinum/paclitaxel/topothecan	-	0	54	75		
Other or not available	5	4	-	-	51	100

Case Material A refers to EOC Samples collected at the S. Chiara Hospital in Trento
Case Material B refers to HG-EOC samples collected at the National Cancer Institute in Milan
Case Material C refers to EOC Samples collected at the National Cancer Institute in Aviano
Abbreviations: FIGO, International Federation of Gynecological and Obstetrics staging system. NED: not evident disease; mRD: minimal residual disease; GRD: gross residual disease.

Table S4. Association between stathmin expression and p53 status.

Total *(n=72)*	***p53 status***				
	wt *n=30*		***Mut**** *n=42*		*p-value*
	N°	%	N°	%	
Stathmin expression					*<0.0001*
Negative-moderate	12	*40*	5	*12*	
Positive	10	*33*	32	*76*	
NV	8	*27*	5	*12*	

DNA extracted from tumors included in Case material B was sequenced for p53 exons 5 to 8. Statistical analysis showed that stathmin overexpression and p53 mutation are highly significantly associated (p<0.0001).

Table S5. Association between stathmin expression and p53 nuclear accumulation.

Total *(n=128)*	***STATHMIN expression***					
	neg/mod *n=54*		***pos*** *n=58*		NV n=16	*p-value*
	N°	%	N°	%		
p53 overexpression						*<0.0001*
Negative	35	*65*	25	*43*	8	
Positive	18	*33*	33	*57*	5	
Missing	1	*2*	0	*0*	3	

Stathmin expression and p53 nuclear accumulation were concomitantly assessed in 111 cases from Case material A. Statistical analysis showed that stathmin overexpression and p53 nuclear accumulation are highly significantly associated (p<0.0001).

Table S6. Cox regression analysis on samples from Case material A (Table S3).

	HR	(95%CI)	P*
**Age at diagnosis**			
>55 versus ≤55	0.77	0.4-1.35	0.36
**Stage**			
III-IV vs I-II	7.4	2.45-22.4	0.0004
**Histotype**			
serous vs others	0.78	0.4-1.48	0.39
**Grade**			
3+undiff vs 1+2	1.16	0.67-1.98	0.59
**Surgical debulking**			
mRD vs NED	4.05	1.71-9.63	0.0014
GRD vs mRD	1.2	0.67-2.14	0.54
**Stathmin expression**			
pos vs neg/mod	1.82	1.05-3.16	0.033

Case material A, multivariate analysis (Cox regression) of progression free survival for clinical and biological variables stratified according to frontline therapy.

Abbreviations: *P value; HR: hazard ratio; CI: confidence interval; mRD: minimal residual disease; NED: not evident disease; GRD: gross residual disease.

A

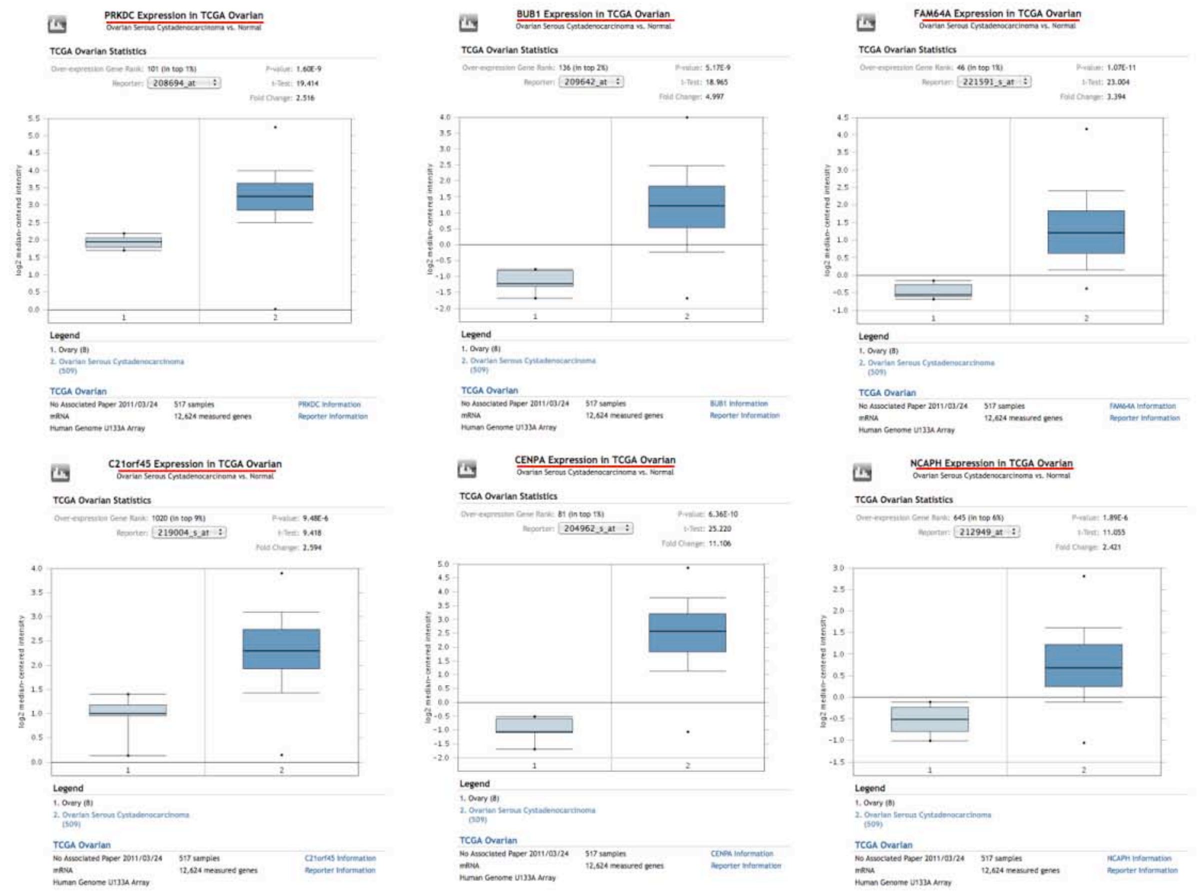

B

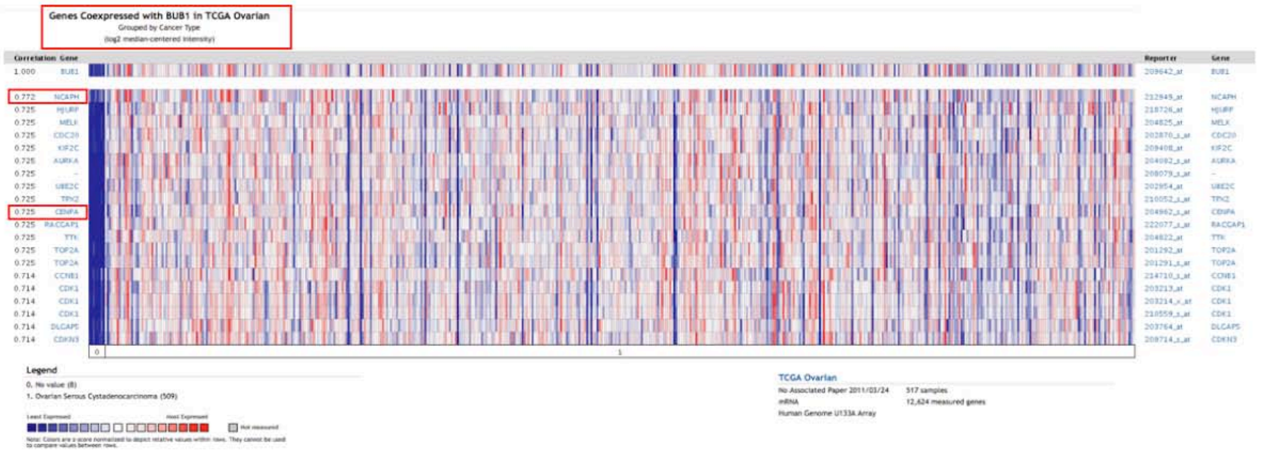

C

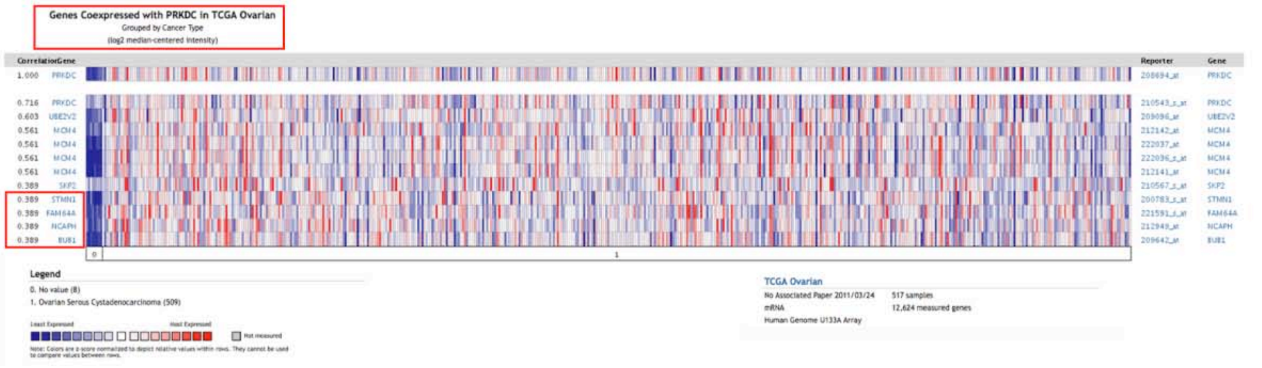

Figure S6. The p53^{MUT}-dependent mitotic signature is overexpressed in type II serous HG-EOC.

(A) DNA-PK, BUB1, FAM64, C21Orf45, CENPA and NACPH mRNA expression in the TCGA Dataset.

(**B)** Analysis of genes co-expressed with BUB1 in the TCGA dataset. Among the first 20 co-expressed genes, NCAPH and CENPA (red boxes) ranked 1 and 10, respectively.

(**C)** Analysis of genes co-expressed with DNA-PK in the TCGA dataset. Stathmin, NCAPH and FAM64 and BUB1 (red box) were found among the first 100 co-expressed genes. All analyses were performed using the Oncomine online resource (https://www.oncomine.org/resource/login.html).

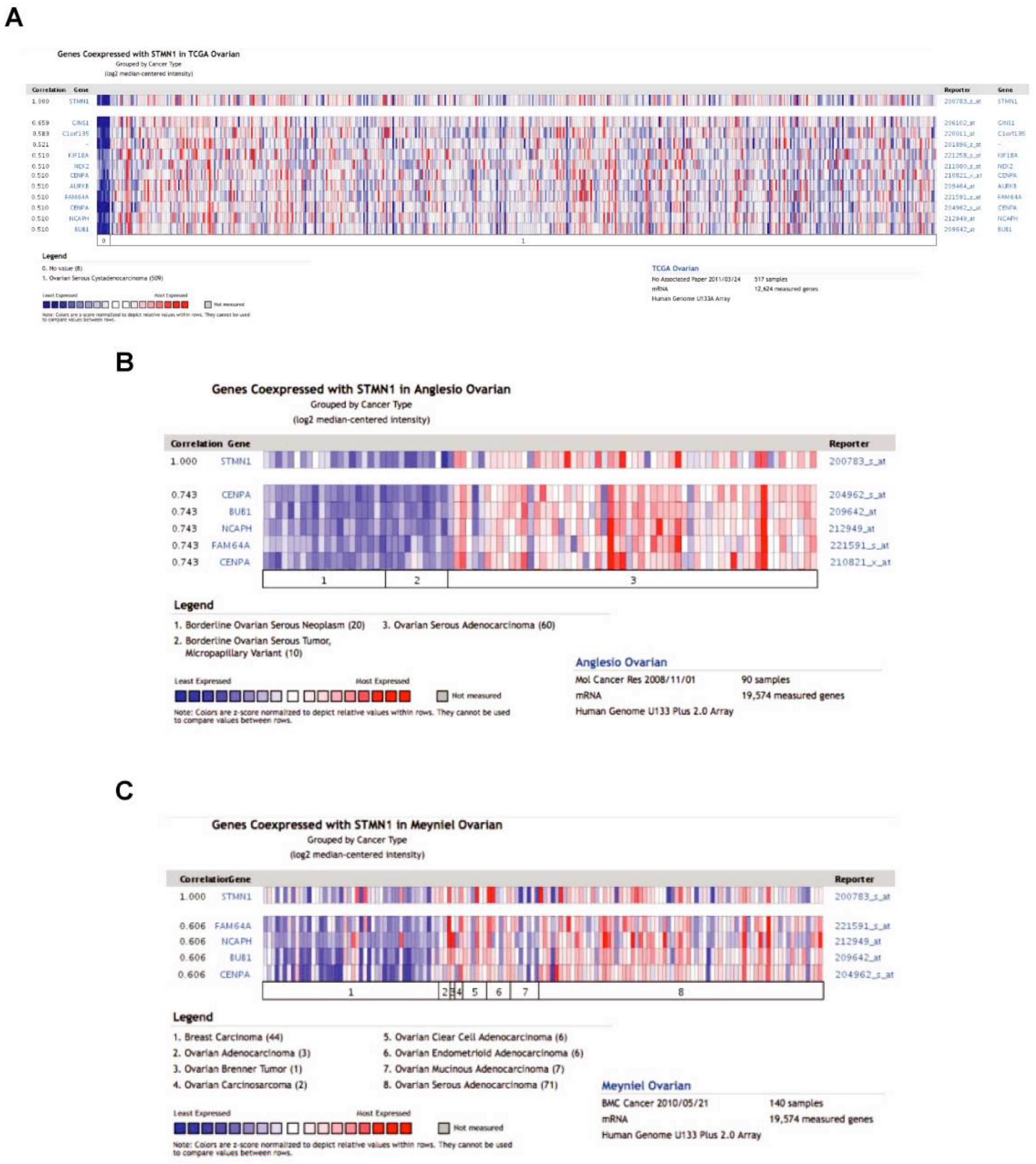

Figure S7. Co-expression of stathmin and the p53^{MUT}-dependent mitotic signature in ovarian carcinomas.

(**A-C**) Analysis of genes co-expressed with stathmin in the TCGA (**A**), the Anglesio (**B**) and the Meyniel (**C**) datasets. In all the datasets among the first 100 genes co-expressed with stathmin in ovarian cancer are present CENPA, NCAPH, BUB1 and FAM64A, as established using the Oncomine online resource.

SUPPORTING METHODS

Compounds and drug treatments

EOC cells were seeded in 96-well culture plates ($1x10^3$ cells/well) and transduced with lentiviral or adenoviral shRNAs. 72 hours after transduction, cells were treated with medium containing Paclitaxel (TAXOL®) or Carboplatin (CBDCA) (TEVA Italia) or vehicle for 14 hours at 37°C at the indicated concentrations. For treatment with the DNA-PK inhibitor, cells were treated with 10μM of NU7441 or DMSO (vehicle). For mitotic shake off, cells were treated with 1μM Nocodazole for 8 hours at 37°C and the last 2 hours treated or not with CBDCA (100 μg/ml for MDAH or 150μg/ml for SKOV-3). Then mitotic cells were divided in two aliquots one subjected to protein extraction and the other re-plated in 96-well culture plates ($1x10^3$ cells/well) and analyzed for their viability by MTS assay 48 hours later. Where indicated, cells were treated with 10μg/ml cycloheximide (Sigma-Aldrich) for 2 and 4 hours or for 6 and 8 hours, as indicated.

Vectors, transfections and viruses

Human stathmin shRNA sequences used in the lentiviral constructs were: 5'-GCGAGAGAAGGATAAGCACAT-3' (sh #1) and CTGGAGGAAATTCAGAAGAAA (sh #2). Human DNA-PK shRNA sequence was 5'- GCAGATAGAAAGCATTACATT-3' (targeting 3'UTR). The control (Ad sh-ctrl) and stathmin (Ad sh-STM) sh-RNA adenoviruses were generated using the BD Knockout Adenoviral RNAi System 2 (BD biosciences) following the manufacturer's procedures and inserting the appropriate target sequence: for human stathmin sh-RNA the target sequence was 5'-CGTTTGCGAGAGAAGGATA-3' (from base 445, Genbank X53305) and the scramble sh-RNA sequence, used as control, was 5' GTCCCATGAAGCTGAGGTC-3'. pCMV empty, pCMV/Neo-p53wt, pCMV/Neo-p53R175H or pCMV/Neo-p53R273H vectors were purchased from Addgene Inc. and transfected in SKOV-3 cells using FuGENE HD Transfection Reagent (Roche) following the manufacturer's procedures. The S15/S37 phosphorylation mutants

(ser→ala) of p53 were generated by PCR on $p53^{R175H}$ or $p53^{R273H}$ vectors using oligonucleotides carrying the indicated mutations.

Preparation of cell lysates, Immunoblotting, and Immunoprecipitation.

Cell lysates were prepared using cold NP-40 lysis buffer (0.5% NP-40, 50 mM HEPES [pH 7], 250 mM NaCl, 5 mM EDTA, 0.5 mM EGTA [pH 8]) plus a protease inhibitor cocktail (Complete, Roche), 1 mM sodium orthovanadate, and 1 mM dithiothreitol, as previously reported (Baldassarre et al., 2005). Protein concentrations were determined using the Bio-Rad protein assay (Bio-Rad). For immunoblotting, equal concentrations of protein samples were separated by 4–20% SDS-PAGE (Criterion precast gel; Bio-Rad) and transferred to nitrocellulose membranes (Hybond C; Amersham). Immunoprecipitations were performed using 700μg of cell lysate in HNTG buffer (20 mM HEPES, 150 mM NaCl, 10% glycerol, 0.1% Triton X-100) plus 1 μg of the indicated specific primary antibody and incubating overnight at 4°C. The immunocomplexes were precipitated by adding protein G agarose-conjugated for additional 2 hours at 4°C and finally separated on SDS-PAGE, for western blot analysis. Immunoblotting were performed using the following primary antibodies: mouse monoclonal anti-stathmin/metablastin (1:250) was from BD biosciences; rabbit polyclonal anti-phospho-p53 (S15, 1:500 and S37, 1:300), rabbit polyclonal anti-ATM (1:1000) and rabbit polyclonal anti-ATR (1:1000) were from Cell Signaling Technology; rabbit polyclonal anti-stathmin/OP18 (1:3000), rabbit polyclonal anti-phospho-DNA-PKcs (T2609, 1:1000) and mouse monoclonal anti BUB1 (1:300) were from Sigma-Aldrich; rabbit polyclonal anti-phospho-histone H3 (S10, 1:500) and mouse monoclonal anti-phospho-histone H2A.X (S139, 1:500) were from Millipore; goat polyclonal anti-vinculin (N-19, 1:1000); rabbit polyclonal anti-DNA-PKcs (H-163, 1:1000) and mouse monoclonal anti-p53 (DO-1, 1:1000) were from Santa Cruz Inc.. Antibodies were visualized with appropriate horseradish peroxidase-conjugated secondary antibodies (GE Healthcare) for chemiluminescent detection (Crescendo, Millipore) or with Alexa-conjugated

secondary antibodies (Invitrogen) for Odyssey infrared detection (LI-COR Biosciences). Quantification of the immunoblots was obtained using the QuantiONE software (Bio-Rad Laboratories) or the Odyssey infrared imaging system (LI-COR Biosciences).

Tumor xenograft

All animal experiments were performed in strict accordance with institutional guidelines on the handling and care of laboratory animals and were approved by CRO Ethical Committee for Animal Experimentation (CESA). MDAH and OVCAR-5 xenografts were established by subcutaneous injection of $2x10^6$ and $4x10^6$ cells, respectively, in 0.1 mL PBS in both flanks of female athymic nude mice (Harlan Laboratories, 8 weeks-old). When tumors were 40-60 mm^3 (about 15 days from injection) animals were randomly divided into groups (5 mice/group) according to experimental design and intratumoral injections were performed with $2x10^9$ total ifu (Ad sh-ctrl at the right flank and Ad sh-STM at the left flank) and repeated 8 times every 3 days. In some cases, mice were also treated with 3 intraperitoneal injections of carboplatin (20mg/Kg) performed every 3 days. Tumor size was measured 3 times a week and volume was calculated (0.5x length x $width^2$). Unless differently indicated, animals were sacrificed after 1 month of treatment and tumor analysis was performed.

TOV-112D and SKOV-3 xenografts were established by subcutaneous injection of cells stably transduced with sh-STM or sh-ctrl, in both flanks of female athymic nude mice ($3.5x10^6$ or $5x10^6$ cells respectively in 0.1 mL PBS) (8 mice/group of treatment). Animals were sacrificed after 1 month. $5x10^6$ of TOV-112D-sh-STM or sh-ctrl cells in 0.1 mL PBS were also intraperitoneally injected in female athymic nude mice (4 mice/group). Animals were sacrificed after 1 month of treatment and tumor analysis was performed.

Time-Lapse video microscopy

For the evaluation of mitosis length, cells were transduced with Ad sh-ctrl or Ad sh-STM (moi=200 for MDAH and moi=500 for SKOV-3 cells) and 72 hours later plated in a 12-well plate ($3.5x10^4$/well for MDAH or $4.5x10^4$/well for SKOV-3). Cells were then incubated at 37°C in 5% CO_2 atmosphere in the Leica Time Lapse AF6000LX workstation equipped with the Leica DMI 6000 motorized microscope and an environmental chamber for the proper setting of temperature humidity and CO_2 concentration. The microscope allows the acquisition of multiple fields on the X, Y and Z axis and is computer assisted. The AF6000 Software (Leica) allows the acquisition of the images on the desiderate frames and periods of time. Images were collected every 15 minutes for 48 hours.

Bioinformatic analysis.

Three independent datasets were used: the TGCA dataset, the Anglesio dataset and the Meyniel dataset. In all datasets analyses were performed using the ONCOMINE software. Statistical analysis was accomplished using the ONCOMINE algorithms.

Patients, tumor specimens and clinical data

The study of clinical samples was performed on three independent primary ovarian cancer specimens panels, collected at primary surgery before any chemotherapeutic treatment from 128 consecutive EOC cases (case material A; surgical resection at S. Chiara Hospital in Trento between 1992 and 1999) from 72 type II EOC cases (case material B; surgical resection at Fondazione IRCCS Istituto Nazionale dei Tumori in Milan between 1990 and 2001) and from 51 consecutive Serous EOC cases (case material B; surgical resection at Centro di Riferimento Oncologico Istituto Nazionale Tumori in Aviano between 2010 and 2012). Patients' baseline characteristics are summarized in Supplementary Table S3 and case material A and B have been previously described (Bagnoli et al., 2009). All histological sections and paraffin blocks were obtained from the Department of Pathology of Milan and Trento institutes. Three different pathologists with

specialized expertise in gynecological pathology reviewed all pathological data. All clinical data and follow-up information were available from the Units of Gynecologic Oncology of both Institutes, as dictated by current follow-up procedures in Milan and Trento (case material A and B). The Institutional Review Board (IRB) approved the use of tissue blocks and patient's record. Tumor staging was in accordance with International Federation of Gynecology and Obstetrics (FIGO) criteria. Primary treatment for all patients was surgery and based on the extent of residual disease patients were divided into three groups: no evident disease (NED); minimal residual disease (mRD; residual tumor smaller than 1cm) and gross residual disease (GRD, residual tumor equal/greater than 1cm). After surgery patients received front-line therapy with standard platinum-based therapeutic schedules (P: platinum without Taxanes; PT: platinum with taxanes; PTT: Platinum with Taxanes and Topotecan), according to the time of accrual and Institutional involvement in International Trials. Tumor grading was the nuclear grading according to the period of patient accrual.

Immunohistochemistry

Stathmin expression was examined by immunohistochemistry (IHC) on archival formalin-fixed-paraffin embedded (FFPE) sections and/or tissue micro array (TMA) using the UltraVision LP detection system HRP polymer (Lab Vision Corporation, Fremont, CA) according to manufacturer's instructions. Briefly, after xylene deparaffinization and alcohol rehydration, sections were subjected to antigen retrieval in Tris-EDTA-citrate buffer (pH7.8) at 96°C for 15 min in autoclave. Endogenous peroxidase was quenched by incubating the slide with 3% H_2O_2 for 10 min. After washing, slides were incubated in blocking solution (Ultra V Block - Lab Vision Corporation, Fremont, CA) for 10 min, followed by 1hour incubation at room temperature with rabbit anti-stathmin primary antibody (Cell Signaling Technology, Danvers, MA). After washing, slides were incubated for 30 min at room temperature with "Primary Antibody Enhancer" (Lab Vision

Corporation, Fremont, CA), washed and incubated with the HRP Polymer (Lab Vision Corporation, Fremont, CA) for 30 min. The peroxidase reaction was developed with 3,3-diaminobenzidine (Dako S.p.A, Milan, Italy) and sections were counterstained with hematoxylin. Slides incubated with "Primary Antibody Enhancer" alone provided negative controls. p53 expression data obtained by IHC using mouse anti-p53 monoclonal antibody DO7 (Novocastra Laboratories, Newcastle upon Tyne; UK) were already available (Bagnoli et al., 2009).

Staining was recorded considering both staining intensity and the percentage of positive cells. Briefly, each core biopsy was evaluated for: staining intensity (0=no staining, 1=faint staining, 2=moderate staining, 3=intense staining); percentage of positive cells (0=non reacting cells, 1=1-10% reacting cells, 2=11-25% reacting cells, 3=26-50% reacting cells, 4= > 50% reacting cells). A final IHC score was obtained by adding the value of the intensity to the percentage category. For case material A, arrayed on TMA, the four cores of each case were scored separately, and the value of the mean score was taken as representative of the case. Only cases with two or more assessable cores were included in the analyses. For statistical analysis, tumors were considered positive for p53-accumulation if they had a score ≥5 (Bagnoli et al., 2009). For stathmin expression they were considered negative, moderate or positive if they had a score ≤3, ≤6 and >6 respectively.

Slides were evaluated by two independent observers, blinded respect to patient characteristics and outcome. All cases with discrepant evaluations were discussed during observation with a double-headed microscope and a consensus was reached.

Immunofluorescence

For immunofluorescence staining, indicated cell lines plated on coverslips for the indicated time were fixed in PBS 4% paraformaldehyde (PFA) at room temperature, permeabilized in PBS 0.2% Triton X-100 and blocked in PBS 1% BSA, 10% normal goat serum. Incubation with primary antibodies (mouse monoclonal anti-phospho Histone H2A.X (S139) 1:500 or rabbit polyclonal anti-

phospho-Histone H3 (S10) 1:300) was performed for 3 hrs at RT (or ON at 4°C) in PBS 1% BSA and 1% normal goat serum. Then samples were washed in PBS and incubated with secondary antibodies for 1 hour at RT. Nuclear staining was achieved using 5 μg/ml Propidium Iodide in PBS supplemented with 100μg/ml of RNase A, for 30 minutes at RT. Stained cells were observed under a confocal laser-scanning microscope (TSP2 Leica) interfaced with a Leica DMIRE2 fluorescent microscope or using a Nikon Diaphot 200 epifluorescent microscope.

Statistical methods and prognostic relationships

For statistical analysis, patients were grouped based on similar clinico-pathological characteristics and age was used as categorical variable (see Tables S3, S4, S5, S6). χ^2 test was used to estimate the association between categorical variables.

In case material A, the effect of biological and clinical-pathological characteristics on progression free survival (PFS) and overall survival (OS) was investigated. OS was the time interval in months between the time of surgery and the date of death for non-censored events or until the date of last contact for censored events when the woman was still alive; PFS was the time in months from completion of chemotherapy until first evidence (clinical, instrumental or biological) of disease recurrence (if a complete response was achieved) or progression. Standard post-chemotherapy surveillance included serial physical examination, serum CA-125 level, and computed tomography scanning as clinically indicated. Follow-up time was the interval between diagnosis and date of death or the last information in the medical records; median follow-up time was 89 months.

OS and PFS curves for each variable were estimated by the Kaplan-Meier method. Differences between curves were assessed with the log-rank test for univariate analysis and the hazard ratio (HR) was estimated with Cox univariate model.

Multivariable Cox regression model was used to evaluate the prognostic impact of stathmin. A two level classification was used for stathmin expression (positive vs negative+moderate), other

covariates were coded as described (Bagnoli et al., 2009) and front-line therapy was used as stratifying factor to take into account its possible non-proportional effects.

The P values of all statistical tests were two-sided and P values ≤ 0.05 were considered significant. All analyses were carried out using R v2.12 statistical language (URL http://www.R-project.org).

Detection of TP53 Gene Mutation

Genomic DNA was extracted, as previously described (Birindelli et al., 2001) from case material B starting from frozen specimen, when available (12 cases), or methylene-blue stained sections from FFPE tissues subjected to a careful microdissection under the microscope to obtain malignant tissues. These cases were subjected to automated DNA sequencing (ABI prism 377, Applied Biosystem) of the most frequently affected exons (5 through 8) of the gene, according to current protocols in use, and each sequence reaction was performed at least twice in sense and antisense strands (Licitra et al., 2006).

Kinexus antibody array

Kinexus Antibody array (KAMTM) produces a pair of 16-bit images, which are captured with a Perkin-Elmer ScanArray Reader laser array scanner. Signal quantification is performed with ImaGene 8.0 from BioDiscovery (El Segundo, CA) with predetermined settings for spot segmentation and background correction. The background-corrected raw intensity data are logarithmically transformed with base 10. Z scores are calculated by subtracting the overall average intensity of all spots within a sample from the raw intensity for each spot, and dividing it by the standard deviations (SD) of all of the measured intensities within each sample. Z ratios are further calculated by taking the difference between the averages of the observed protein. A Z ratio of ± 1.4 is inferred as significant.

SUPPORTING REFERENCES

Bagnoli, M., Ambrogi, F., Pilotti, S., Alberti, P., Ditto, A., Barbareschi, M., Galligioni, E., Biganzoli, E., Canevari, S., Mezzanzanica, D. (2009). c-FLIPL expression defines two ovarian cancer patient subsets and is a prognostic factor of adverse outcome. Endocr. Relat. Cancer *16*: 443-453.

Baldassarre, G., Belletti, B., Nicoloso, M.S., Schiappacassi, M., Vecchione, A., Spessotto, P., Morrione, A., Canzonieri, V., Colombatti, A. (2005). p27Kip1-stathmin interaction influences sarcoma cell migration and invasion. Cancer Cell *7*: 51– 63.

Birindelli, S., Perrone, F., Oggionni, M., Lavarino, C., Pasini, B., Vergani, B., Ranzani, G.N., Pierotti, M.A., Pilotti, S. (2001). Rb and TP53 pathway alterations in sporadic and NF1-related malignant peripheral nerve sheath tumors. Lab. Invest. *81*: 833-844.

Licitra, L,. Perrone, F., Bossi, P., Suardi, S., Mariani, L., Artusi, R., Oggionni, M., Rossini, C., Cantù, G., Squadrelli, M., et al. (2006). High-risk human papillomavirus affects prognosis in patients with surgically treated oropharyngeal squamous cell carcinoma. J. Clin. Oncol. *24*: 5630-5636.
